# The impact of behavioral weight management interventions on eating behavior traits in adults with overweight or obesity: A systematic review and meta‐analysis

**DOI:** 10.1111/obr.13871

**Published:** 2024-11-21

**Authors:** Laura Kudlek, Patricia Eustachio Colombo, Amy Ahern, Struan Tait, Natasha Reid, Milindu Wickramarachchi, Aiswarya Lakshmi, Stephen J. Sharp, Marie Spreckley, Julia Mueller, Rebecca A. Jones

**Affiliations:** ^1^ MRC Epidemiology Unit, School of Clinical Medicine University of Cambridge Cambridge UK; ^2^ School of Clinical Medicine University of Cambridge Cambridge UK

**Keywords:** behavioral weight management, eating behavior traits, emotional eating, obesity treatment

## Abstract

Eating behavior traits (EBTs), defined as personal tendencies determining food intake, can be useful targets for behavioral weight management interventions. Previous reviews have examined the impact of specific intervention types on EBTs, not reflecting the breadth of interventions used in practice. This systematic review and meta‐analysis synthesized evidence on the impact of all types of behavioral weight management interventions on EBTs. We searched seven databases and included randomized controlled trials reporting EBT outcomes following behavioral weight management interventions delivered to adults with overweight or obesity. Using random‐effects meta‐analyses, we synthesized findings from 27 trials at the end of intervention and 12 months (±3 months) post intervention. We found evidence to suggest that interventions improved uncontrolled eating, external eating, susceptibility to hunger, restraint and intuitive eating at intervention end when compared with waitlist, minimal or usual care control. We found no evidence of effects on emotional eating, disinhibition, and hedonic hunger. At follow‐up, effects on restraint remained, but we found no evidence of an effect on emotional eating, hedonic hunger and uncontrolled eating. Findings were limited by low numbers of contributing trials. More high‐quality trials reporting EBTs are needed to better understand the impact of adult behavioral weight management interventions on EBTs.

**PROSPERO registration number:** CRD42022367505.

**Abbreviations**
BMI: body mass index
CI: confidence interval
DEBQ: Dutch Eating Behavior Questionnaire
EBTs: eating behavior traits
ecSI‐2: Satter Eating Competence Inventory
EDE‐R: Eating Disorder Examination Questionnaire Restraint Subscale
EES: Emotional Eating Scale
EOQ: Emotional Overeating Questionnaire
HQ‐EWB: Eating Habits Questionnaire for Patients with Overweight and Obesity ‐ Eating for Psychological Wellbeing subscale
HTAS: Health and Taste Attitude Scales
IES: Intuitive Eating Scale
MES: Mindful Eating Scale
PFS: Power of Food Scale
PI: prediction interval
RCTs: randomized controlled trials
SMD: standardized mean difference
TFEQ: Three Factor Eating Questionnaire

## 1 INTRODUCTION

More than one billion people worldwide are affected by obesity,^1^ a chronic relapsing condition^2^ that is associated with adverse physical and mental health risks, such as metabolic and cardiovascular disorders, some cancers and mood disorders.^3^, ^4^ Behavioral approaches can achieve weight loss, leading to meaningful improvements in health, such as a reduced likelihood of developing type 2 diabetes.^5^, ^6^, ^7^ These include a variety of interventions aiming to support weight loss through changes in diet, physical activity, cognitions, emotions, and other health promoting behaviors. However, there is considerable individual variability in responses to behavioral treatments for obesity^8^ and weight‐loss is often short‐term.^9^, ^10^ Identifying modifiable predictors of weight loss that can be targeted in interventions could improve treatment effectiveness.

Individuals' reactions to food, food‐related cues, and food intake play an important role in the development and maintenance of overweight and obesity. These behaviors are thought to underlie relatively stable tendencies, known as eating behavior traits (EBTs), which could be responsible for part of the variability in treatment responses.^11^ Behavioral Susceptibility Theory^11^, ^12^ proposes that EBTs interact with the obesogenic food environment, either increasing or decreasing an individual's susceptibility to overeat, by determining the type, amount and frequency of food consumed, as well as when to start and stop eating in response to food availability. For example, commonly assessed EBTs are restraint (the conscious effort to restrict food intake in order to control weight), uncontrolled eating (the tendency to overeat in response to external food cues and feelings of hunger), and emotional eating (the tendency to overeat in response to negative emotions). Previous studies found that changes in EBTs during weight loss treatment were associated with greater weight loss and weight loss maintenance.^13^, ^14^, ^15^, ^16^, ^17^, ^18^, ^19^, ^20^, ^21^, ^22^ This suggests that addressing EBTs in weight management interventions could improve their long‐term effectiveness.

Several reviews have synthesized evidence on the impact of specific types of behavioral interventions on different psychological and dysregulated eating outcomes, including a variety of EBTs. Findings often showed small to medium changes, such as reductions in uncontrolled^23^, ^24^ and emotional eating^23^, ^24^, ^25^, ^26^, ^27^ and increases in restraint.^24^, ^25^ However, these reviews investigated the effects of specific types of interventions on EBTs. For example, Jacob et al.^25^ focused on second wave cognitive behavioral interventions, Lawlor et al.^23^ on third wave cognitive behavioral interventions, and Chew et al.^24^ and Smith et al.^27^ on interventions with content that specifically targets emotional eating. Even more specific intervention types were studied by Chew et al.,^28^ DiSante et al.^26^ and Iturbe et al.^29^ who examined the effects of acceptance and commitment therapy, and by O'Reilly et al.^30^ and Carrière et al.^31^ who examined effects of mindfulness‐based interventions. These interventions do not reflect the range of behavioral weight management interventions used in practice, where many do not target specific EBTs and provide little to no psychological support. Thus, it is currently unknown whether behavioral interventions more generally impact EBTs.

The aim of this review was to assess the impact of all types of behavioral weight management interventions compared with minimal, inactive or “usual care” control groups on EBTs in adults living with overweight and obesity. We also explored whether intervention, comparator and participant characteristics influence the impact of interventions on EBTs.

## 2 METHODS

This systematic review and meta‐analysis followed guidance from the Preferred Reporting Items for Systematic Reviews and Meta‐Analyses (PRISMA) statement.^32^

### 2.1 Study identification

#### 2.1.1 Eligibility criteria

Trials were considered eligible for inclusion if they met the following criteria:

- **Study designs:** Original peer‐reviewed primary research articles reporting randomized controlled trials (RCTs) or cluster RCTs were included, with no restrictions on year of publication.
- **Population:** Adults aged ≥18 years living with overweight or obesity (body mass index [BMI] ≥ 25 kg/m^2^) at baseline were included. Trials that included people with comorbidities were eligible, but we excluded trials that focused exclusively on populations with a specific physical or mental comorbidity, specific EBT profiles, or on populations that were pregnant/postnatal. Thus, it was possible that some, but not all participants within any given trial, had additional conditions such as type 2 diabetes or binge eating disorder.
- **Interventions:** Trials were included if they evaluated any type of behavioral weight management intervention, defined as a structured program designed to help individuals achieve a healthy weight without surgical or pharmacological intervention. We included interventions if they aimed to support weight management through mechanisms such as changes in diet, physical activity, sleep, cognitions or emotion regulation. No restrictions were placed on intervention delivery and duration. Interventions where participants resided on the treatment site (e.g., inpatient interventions, interventions at army camps) were excluded.
- **Comparators:** Trials with an inactive/waitlist/minimal/usual care control group were included.
- **Outcomes:** Trials were required to have reported one or more EBT outcome at intervention end or follow‐up (12 months [±3 months] from intervention end). EBTs had to be reported either as post intervention observed mean scores (“post intervention outcomes”) or as mean change (“change outcomes”). Alternatively, trials were also eligible if they reported a standardized mean difference (SMD) calculated using Hedges' g and standard error of the intervention effect on change in EBTs in the intervention group compared with the control group. EBTs were defined as behavioral tendencies or characteristics of an individual that are relatively consistent across time and situations and determine the individual's food intake (e.g., type, amount, frequency, initiation and cessation of food consumption). Outcomes describing traits relating to eating attitudes and eating disorder symptoms, such as food preoccupation, bulimia and oral control, were excluded, as these have been covered in another recent review.^33^
- **Language:** Only trials published in English language were included.

#### 2.1.2 Information sources and search strategy

Seven databases were searched from inception to 29th September 2022: AMED, ASSIA, CINAHL, Cochrane database (CENTRAL), Embase, MEDLINE, PsycINFO. Detailed search strategies were developed by RAJ, with input from ALA, LK, ST, and a librarian (IK). The search strategy contained relevant keywords and headings based on previous review articles and was based on the concepts: (1) people living with overweight/obesity AND (2) weight management interventions AND (3) EBT outcomes AND (4) study designs (Supporting Information). The search was restricted to English language papers. Reference lists of previous relevant reviews were also searched.^24^, ^27^, ^34^

#### 2.1.3 Study selection

Two authors independently conducted title and abstract and full text screening. LK screened each record and duplicate screening was conducted by PEC, ST, NR, MW, and AL. Discrepancies were resolved through discussion with a third reviewer (RAJ). Where results from the same trial were reported in several publications, records were combined. No automation tools were used in this process.

### 2.2 Data collection

Records were extracted by one author (PEC, ST, NR, MW, AL, JM, and MS) and checked by LK. Discrepancies were resolved through discussion with a third reviewer (RAJ). If publications described measuring eligible EBTs but did not report results in the format required or only provided partial data, we requested data via email (*N* = 11). Authors were sent one reminder and were given a minimum of 2 months to respond before the trial was excluded. Four authors provided data; three did not respond, and four were unable to share data.

#### 2.2.1 Data items

Outcomes were extracted at the end of intervention and at 12 months (±3 months) from intervention end in the form of post intervention or change outcomes. For cluster RCTs, we extracted the SMD (Hedges' g) and standard error adjusted for the cluster randomized design. If the intervention effect did not account for clustering or it was not provided in the required format, we requested authors to provide an intervention effect and standard error that did account for clustering. One author provided data from cluster RCTs in the required format.

We also extracted information on the trial (setting, country), participants (*N* randomized, *n* per intervention arm, sex, age, ethnicity, income, education, baseline BMI) and interventions and comparisons (target behavior, delivery mode, delivery format, duration, frequency, intensity, intervention components).

### 2.3 Risk of bias assessment

Risk of bias was assessed by two independent reviewers using the Cochrane risk‐of‐bias tool for randomized trials version 2 (RoB‐2).^35^ LK assessed each record and duplicate assessments were performed by ST, MS, JM, NR, PEC, MW, or RAJ. Discrepancies were resolved through discussion with a third reviewer (RAJ). Ratings from domain 4 (“Risk of bias in measurement of the outcome”) were excluded from the overall assessment as all eligible EBT measures are self‐reported and all behavioral trials are non‐blinded to the participants (the outcome assessors). Additionally, domain 1 (“Randomisation process”) was rated as high risk of bias if baseline differences in EBTs between intervention and control groups were deemed at risk of impacting effect estimates.

### 2.4 Data analysis

Data analyses followed a pre‐specified analysis plan (PROSPERO CRD42022367505).

#### 2.4.1 Data preparation

Standard errors and confidence intervals were transformed into standard deviations using recommended approaches from the Cochrane handbook.^36^ In trials with multiple eligible intervention arms and one shared comparison arm, we either combined intervention arms according to recommendations from the Cochrane handbook^36^ or divided the number of participants in the shared comparison arm by the number of intervention arms. Whether intervention arms were combined or kept separate was determined based on whether or not the additional intervention arm would contribute toward the exploration of heterogeneity in subgroup analyses. This approach was chosen to retain sufficient detail to investigate heterogeneity while ensuring that no group contributed more than once to the analyses (i.e., preventing the risk of a unit‐of‐analysis error).

#### 2.4.2 Synthesis of results

SMDs were calculated using Hedge's g or provided directly by authors from cluster RCTs. Intervention effects were combined across trials using random‐effects meta‐analysis at (a) end of intervention and (b) at 12 months (±3 months) from the end of intervention to investigate immediate and long‐term effects on EBTs. Heterogeneity was assessed by calculating I‐squared and prediction intervals. A prediction interval describes the range within which 95% of the true effects are expected to lie. Stata v.17 was used for all analyses.^37^

#### 2.4.3 Additional analyses

Random‐effects meta‐analyses were conducted in the subgroups listed below to explore the influence of intervention, comparator, or participant characteristics on effect sizes. Interventions were classified into subgroups by the lead author based on duplicate extracted study characteristics data.

- **Intervention type:** Interventions with the majority of content being considered (a) standard behavioral (e.g., exercise, education, nutrition counseling, behavior change techniques) (b) psychological second wave (Cognitive Behavioral Therapy) or (c) psychological third wave (e.g., Acceptance and Commitment Therapy [ACT], Mindfulness).
- **Intervention duration:** (a) 12 weeks and less (b) more than 12 weeks.
- **Intervention delivery:** (a) Face‐to‐face (b) remote (c) both.
- **Intervention format:** (a) Individual (b) group (c) both.
- **Comparator intensity**: (a) No intervention (up to one standardized contact) (b) intervention (repeated contacts or personalized content).
- **Baseline BMI**: (a) Average BMI of 25–35 kg/m^2^ (b) Average BMI of >35 kg/m^2^.

Sensitivity analyses were conducted to investigate (a) the potential impact of risk of bias on effect estimates; trials judged to be high risk of bias were removed from pooled estimates, and (b) the potential impact of cluster RCTs on effect estimates; cluster RCTs were removed from pooled estimates.

Contour‐enhanced funnel plots of individual trial effect sizes were produced for all outcomes to assess the potential for publication bias.

## 3 RESULTS

### 3.1 Study selection

**FIGURE 1:**
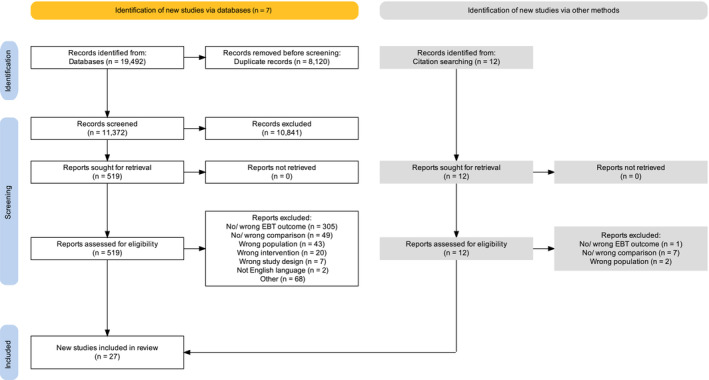
Preferred Reporting Items for Systematic Reviews and Meta‐analysis (PRISMA) flowchart for the inclusion of trials.

A total of 11,384 records were screened and 27 trials examining 30 interventions were included^21^, ^38^, ^39^, ^40^, ^41^, ^42^, ^43^, ^44^, ^45^, ^46^, ^47^, ^48^, ^49^, ^50^, ^51^, ^52^, ^53^, ^54^, ^55^, ^56^, ^57^, ^58^, ^59^, ^60^, ^61^, ^62^, ^63^ (Figure 1).

### 3.2 Study characteristics

Study characteristics are summarized in Table 1, with further detail in Table S1. Of the 27 included trials, 26 were RCTs^21^, ^38^, ^39^, ^40^, ^41^, ^42^, ^43^, ^44^, ^45^, ^46^, ^47^, ^48^, ^49^, ^50^, ^51^, ^52^, ^53^, ^54^, ^55^, ^56^, ^57^, ^58^, ^59^, ^60^, ^61^, ^62^ and one was a cluster RCT.^63^ Sample sizes ranged from 30^52^ to 1267^61^ participants, amounting to a total of 6684 participants. Most trials were conducted in the United States of America (USA) (*N* = 7)^38^, ^41^, ^43^, ^51^, ^53^, ^56^, ^63^ or in the United Kingdom (UK) (*N* = 4).^39^, ^40^, ^50^, ^61^ Eleven trials were conducted in European high‐income countries (Finland, Germany, Portugal, Switzerland, Netherlands, Poland, Spain)^21^, ^44^, ^45^, ^46^, ^47^, ^49^, ^52^, ^54^, ^55^, ^57^, ^59^ and one in Canada.^58^ Three trials were conducted in middle‐income countries (Iran, Brazil).^42^, ^48^, ^60^ One trial was conducted across three different high‐income countries (UK, Germany, Australia).^62^

**TABLE 1:** Overview of characteristics of included trials.

Study characteristics	*N* trials	Citations
Study design		
RCT	26	^38^, ^39^, ^40^, ^41^, ^42^, ^43^, ^44^, ^45^, ^46^, ^47^, ^48^, ^49^, ^50^, ^51^, ^52^, ^53^, ^54^, ^55^, ^56^, ^57^, ^58^, ^59^, ^60^, ^61^, ^62^
Cluster RCT	1	^63^
Overall risk of bias rating		
Low	18	^39^, ^40^, ^41^, ^42^, ^44^, ^45^, ^46^, ^47^, ^48^, ^50^, ^52^, ^53^, ^54^, ^55^, ^58^, ^61^, ^62^, ^63^
Some concerns	5	^43^, ^49^, ^56^, ^57^, ^59^
High	4	^38^, ^51^, ^60^
Trial location^a^		
United States of America	7	^38^, ^41^, ^43^, ^51^, ^53^, ^56^, ^63^
United Kingdom	5	^39^, ^40^, ^50^, ^61^, ^62^
Finland	3	^46^, ^54^
Brazil	2	^42^, ^60^
Germany	3	^52^, ^55^, ^62^
Portugal	2	^47^, ^59^
Australia	1	^62^
Canada	1	^58^
Iran	1	^48^
Netherlands	1	^44^
Switzerland	1	^49^
Poland	1	^57^
Spain	1	^45^
Sample size (Total *N* randomized)		
<100	12	^38^, ^41^, ^43^, ^47^, ^48^, ^52^, ^53^, ^56^, ^58^, ^59^, ^60^
100–300	8	^40^, ^42^, ^44^, ^46^, ^49^, ^54^, ^55^, ^57^
300–500	4	^39^, ^45^, ^50^, ^51^
>500	3	^61^, ^62^, ^63^
Trials reporting EBT outcomes at intervention end		
Contextual skills	1	^54^
Disinhibition	4	^41^, ^49^, ^52^, ^63^
Emotional eating	17	^38^, ^39^, ^40^, ^43^, ^44^, ^46^, ^47^, ^48^, ^50^, ^51^, ^53^, ^54^, ^55^, ^56^, ^57^, ^60^, ^62^
Emotional eating, anger	1	^58^
Emotional eating, anxiety	1	^58^
Emotional eating, depression	1	^58^
External eating	3	^44^, ^55^, ^56^
Hunger, hedonic	2	^59^, ^61^
Hunger, susceptibility	3	^41^, ^49^, ^52^
Internal regulation	1	^54^
Intuitive/mindful eating	3	^42^, ^54^, ^57^
Pleasure	1	^54^
Restraint	19	^38^, ^39^, ^40^, ^41^, ^44^, ^46^, ^48^, ^49^, ^50^, ^51^, ^52^, ^53^, ^54^, ^55^, ^56^, ^60^, ^61^, ^62^, ^63^
Reward	1	^54^
Uncontrolled eating	12	^39^, ^40^, ^46^, ^47^, ^48^, ^50^, ^51^, ^53^, ^54^, ^57^, ^60^, ^62^
Trials reporting EBT outcomes at 12‐month follow‐up		
Disinhibition	1	^49^
Emotional eating	4	^39^, ^45^, ^55^
External eating	1	^55^
Hunger, hedonic	1	^61^
Hunger, susceptibility	1	^49^
Restraint	5	^39^, ^49^, ^55^, ^61^
Uncontrolled eating	2	^39^
Questionnaires used		
TFEQ		
TFEQ‐51	5	^41^, ^49^, ^52^, ^61^, ^63^
TFEQ‐R18 or TFEQ‐R21	13	^39^, ^40^, ^46^, ^47^, ^48^, ^50^, ^51^, ^53^, ^54^, ^57^, ^60^, ^62^
DEBQ	4	^43^, ^44^, ^55^, ^56^
MES	2	^42^, ^57^
IES	1	^54^
PFS	2	^59^, ^61^
EDE‐R	1	^38^
EOQ	1	^38^
EES	1	^58^
HQ‐EWB	1	^45^
ecSI‐2	1	^54^
HTAS	1	^54^

Intervention characteristics	*N* interventions [*N* trials]	Citations
Intervention type		
Standard behavioral	13 [12]	^38^, ^39^, ^41^, ^45^, ^46^, ^51^, ^52^, ^59^, ^61^, ^62^, ^63^
Second wave psychological	3 [3]	^44^, ^49^, ^58^
Third wave psychological	11 [10]	^40^, ^42^, ^43^, ^47^, ^50^, ^53^, ^54^, ^56^, ^57^, ^60^
Other	3 [3]	^38^, ^48^, ^55^
Delivery mode		
Face to face	18 [17]	^39^, ^42^, ^44^, ^45^, ^46^, ^47^, ^48^, ^49^, ^51^, ^54^, ^55^, ^56^, ^59^, ^60^, ^61^, ^62^
Remote	8 [8]	^40^, ^41^, ^43^, ^50^, ^53^, ^54^, ^57^, ^58^
Mixed	4 [3]	^38^, ^52^, ^63^
Delivery format		
Individual	12 [12]	^40^, ^41^, ^43^, ^44^, ^46^, ^50^, ^53^, ^54^, ^57^, ^58^, ^59^, ^63^
Group	14 [13]	^39^, ^42^, ^45^, ^47^, ^48^, ^49^, ^52^, ^54^, ^55^, ^56^, ^60^, ^61^, ^62^
Mixed	4 [3]	^38^, ^51^
Type of health professional delivering the intervention		
Trained non‐specialists	7 [6]	^39^, ^49^, ^50^, ^61^, ^62^, ^63^
Trained healthcare providers^b^	5 [4]	^38^, ^45^, ^46^, ^58^
Clinical nutritionists or dieticians	7 [7]	^45^, ^46^, ^51^, ^52^, ^59^, ^60^
(Student) psychologists	8 [7]	^43^, ^47^, ^52^, ^54^, ^55^, ^60^
Mixed	4 [4]	^45^, ^46^, ^52^, ^60^
Other or unknown	6 [6]	^40^, ^41^, ^42^, ^44^, ^48^, ^56^
Intervention duration^c^		
≤12 weeks	20 [18]	^38^, ^39^, ^40^, ^42^, ^43^, ^44^, ^45^, ^47^, ^48^, ^50^, ^52^, ^53^, ^54^, ^55^, ^56^, ^57^, ^58^, ^61^
12–26 weeks	4 [4]	^41^, ^49^, ^59^
≥26 weeks	5 [5]	^46^, ^51^, ^61^, ^62^, ^63^
Comparison type		
Waitlist	10 [9]	^41^, ^43^, ^50^, ^51^, ^52^, ^53^, ^54^, ^56^, ^57^
Minimal	4 [4]	^40^, ^46^, ^59^
Usual care	16 [14]	^38^, ^39^, ^42^, ^44^, ^45^, ^47^, ^48^, ^49^, ^55^, ^58^, ^60^, ^61^, ^62^, ^63^
Comparison intensity		
No intervention^d^	19 [16]	^38^, ^41^, ^43^, ^44^, ^46^, ^48^, ^49^, ^50^, ^51^, ^52^, ^53^, ^54^, ^56^, ^57^, ^61^, ^62^
Intervention^e^	11 [11]	^39^, ^40^, ^42^, ^45^, ^47^, ^55^, ^58^, ^59^, ^60^, ^63^

*Abbreviations:* DEBQ, Dutch Eating Behavior Questionnaire; EBT, eating behavior trait; ecSI‐2, Satter Eating Competence Inventory; EDE‐R, Eating Disorder Examination Questionnaire Restraint Subscale; EES, Emotional Eating Scale; EOQ, Emotional Overeating Questionnaire; HQ‐EWB, eating habits questionnaire for patients with overweight and obesity eating for psychological wellbeing subscale; HTAS, Health and Taste Attitude Scales; IES, Intuitive Eating Scale; MES, Mindful Eating Scale; *N*, number of; PFS, Power of Food Scale; RCT, randomized controlled trial; TFEQ, Three Factor Eating Questionnaire.

^a^ Jebb et al.^62^ was conducted across three countries.

^b^ Trained healthcare providers including nurses, psychometrists, and medical assistants.

^c^ Campos et al.^60^ had an unknown intervention duration.

^d^ No intervention defined as including one standardized email or one standardized provision of material.

^e^ Intervention defined as including at least one contact with some degree of personalization or several contacts.

Thirty intervention arms were included from the 27 included trials. Interventions had a mean duration of 17.42 weeks (SD 19.29) with the shortest intervention being two weeks^57^ and the longest intervention 104 weeks.^63^ Intervention types included 13 standard behavioral interventions (e.g., education, nutrition counseling, behavior change techniques, and physical activity guidance),^21^, ^38^, ^39^, ^41^, ^45^, ^46^, ^51^, ^52^, ^59^, ^61^, ^62^, ^63^ three second wave Cognitive Behavioral Therapy based interventions,^44^, ^49^, ^58^, and 11 third wave cognitive behavioral interventions (e.g., Acceptance and Commitment Therapy, Mindfulness Based Interventions, Intuitive Eating, Attentive Eating, or Emotional Schema Therapy).^40^, ^42^, ^43^, ^47^, ^50^, ^53^, ^54^, ^56^, ^57^, ^60^ Three additional interventions were based on Motivational Interviewing^38^, ^48^ and Cognitive Remediation Therapy.^55^ Most interventions were delivered in person (*N* = 18)^21^, ^39^, ^42^, ^44^, ^45^, ^46^, ^47^, ^48^, ^49^, ^51^, ^54^, ^55^, ^56^, ^59^, ^60^, ^61^, ^62^ and in a group‐based format (*N* = 14).^39^, ^42^, ^45^, ^47^, ^48^, ^49^, ^52^, ^54^, ^55^, ^56^, ^60^, ^61^, ^62^ Remote interventions (*N* = 8) were exclusively delivered in an individual‐based format.^40^, ^41^, ^43^, ^50^, ^53^, ^54^, ^57^, ^58^ Few interventions combined in person and remote approaches (*N* = 4),^38^, ^52^, ^63^ or individual‐ and group‐based formats (*N* = 4).^21^, ^38^, ^51^

Twelve eligible EBTs were identified in included trials (restraint [*N* = 19],^38^, ^39^, ^40^, ^41^, ^44^, ^46^, ^48^, ^49^, ^50^, ^51^, ^52^, ^53^, ^54^, ^55^, ^56^, ^60^, ^61^, ^62^, ^63^ emotional eating [*N* = 18],^38^, ^39^, ^40^, ^43^, ^44^, ^46^, ^47^, ^48^, ^50^, ^51^, ^53^, ^54^, ^55^, ^56^, ^57^, ^60^, ^62^ uncontrolled eating [*N* = 12],^39^, ^40^, ^46^, ^47^, ^48^, ^50^, ^51^, ^53^, ^54^, ^57^, ^60^, ^62^ disinhibition [*N* = 4],^41^, ^49^, ^52^, ^63^ intuitive/mindful eating [*N* = 3],^42^, ^54^, ^57^ external eating [*N* = 3],^44^, ^55^, ^56^ susceptibility to hunger [*N* = 3],^41^, ^49^, ^52^ hedonic hunger [*N* = 2],^59^, ^61^ contextual skills [*N* = 1],^54^ internal regulation [*N* = 1],^54^ reward [*N* = 1],^54^ and pleasure [*N* = 1]).^54^ Most commonly used questionnaires to assess EBTs included the 18‐item Three Factor Eating Questionnaire (TFEQ) (*N* = 13),^21^, ^39^, ^40^, ^46^, ^47^, ^48^, ^50^, ^51^, ^53^, ^54^, ^57^, ^60^, ^62^ the 51‐item TFEQ (*N* = 5),^41^, ^49^, ^52^, ^61^, ^63^ and the Dutch Eating Behavior Questionnaire (DEBQ) (*N* = 4).^43^, ^44^, ^55^, ^56^

### 3.3 Risk of bias

Most trials were deemed low risk of bias (66.66%; *N* = 18).^39^, ^40^, ^41^, ^42^, ^44^, ^45^, ^46^, ^47^, ^48^, ^50^, ^52^, ^53^, ^54^, ^55^, ^58^, ^61^, ^62^, ^63^ Five trials (18.51%) were classified at some risk of bias.^43^, ^49^, ^56^, ^57^, ^59^ Four trials were deemed high risk of bias (14.81%).^21^, ^38^, ^51^, ^60^ Figure S1 presents risk of bias ratings across trials.

### 3.4 Intervention effects on eating behavior traits

#### 3.4.1 Restraint

**FIGURE 2:**
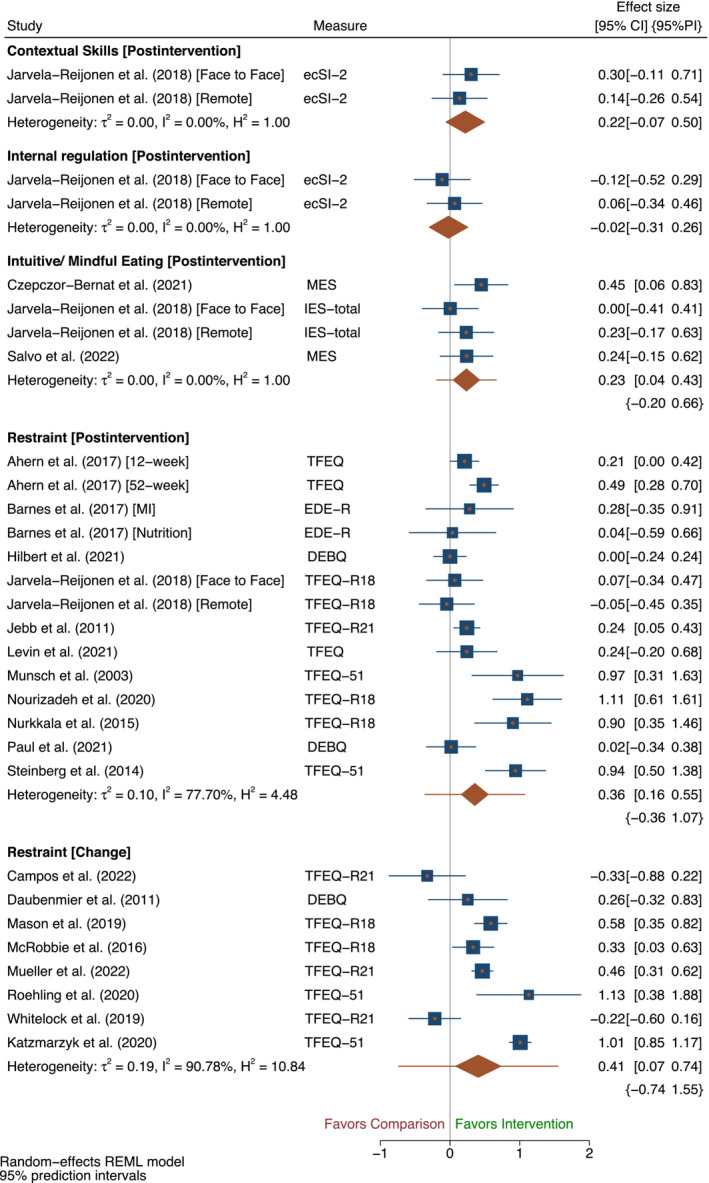
Outcomes where the desired intervention effect is an increase in the trait at intervention end. *Note*: The size of blue squares representing each trial are proportional to the study weight. The width of the orange diamond representing overall effect size corresponds to the length of the confidence interval (CI), and its orange whiskers correspond to the length of the prediction interval (PI). The height of the diamond is irrelevant. *Abbreviations*: DEBQ, Dutch Eating Behaviour Questionnaire; ecSI‐2, Satter Eating Competence Inventory; EDE‐R, Eating Disorder Examination Questionnaire Restraint Subscale; IES, Intuitive Eating Scale; MES, Mindful Eating Scale; TFEQ‐51, Three Factor Eating Questionnaire original 51 item version; TFEQ‐R18, Three Factor Eating Questionnaire revised 18 item version; TFEQ‐R21, Three Factor Eating Questionnaire revised 21 item version.

Eleven trials with 14 intervention arms reported post intervention outcomes, and eight intervention arms reported change outcomes for restraint at intervention end. There was evidence of an effect in favor of interventions for restraint at intervention end (post intervention: SMD 0.36 [95% CI 0.16, 0.55]; change: SMD 0.41 [95% CI 0.07, 0.74]). Considerable heterogeneity was present for both post intervention outcomes (*I* ^2^ = 77.7%) and change outcomes (*I* ^2^ = 90.78%), and wide prediction intervals indicated a possibility of different effects being found in future studies for both outcomes (post intervention: [95% PI −0.36, 1.07]; change: [95% PI −0.74, 1.55]) (Figure 2).

There was evidence of an effect on restraint at follow‐up for post intervention scores (post intervention: SMD 0.18 [95% CI 0.05, 0.30], *N* = 4, *I* ^2^ = 0.00%), however the prediction interval was wide and indicated inconclusive effects for future studies ([95% PI −0.09, 0.44]). We found no evidence of an effect at follow‐up for change scores (change: SMD 0.19 [95% CI −0.05, 0.43], *N* = 2, *I* ^2^ = 0.00%) (Figure S2).

Findings remained similar after removing intervention arms from trials deemed at high risk of bias (*N* = 4) (post intervention: SMD 0.38 [95% CI 0.16, 0.61], [95% PI −0.42, 1.18], *N* = 12, *I* ^2^ = 82.21%; change: SMD 0.48 [95% CI 0.09, 0.86], [95% PI −0.85, 1.80], *N* = 6, *I* ^2^ = 91.11%) (Table S3) (Figure S4), and after removing cluster RCTs at the end of intervention (*N* = 1) (change: SMD 0.30 [95% CI 0.00, 0.61], [95% PI −0.69, 1.29], *N* = 7, *I* ^2^ = 82.49%) (Table S6) (Figure S8). No cluster RCTs were included at follow‐up. After removing trials at high risk of bias at follow‐up (*N* = 1), only one intervention arm remained for change outcomes that found no evidence of an effect on restraint (change: SMD 0.17 [95% CI −0.10, 0.44) (Figure S6). No trials reporting post intervention outcomes at follow‐up were deemed at high risk of bias.

#### 3.4.2 Emotional eating

**FIGURE 3:**
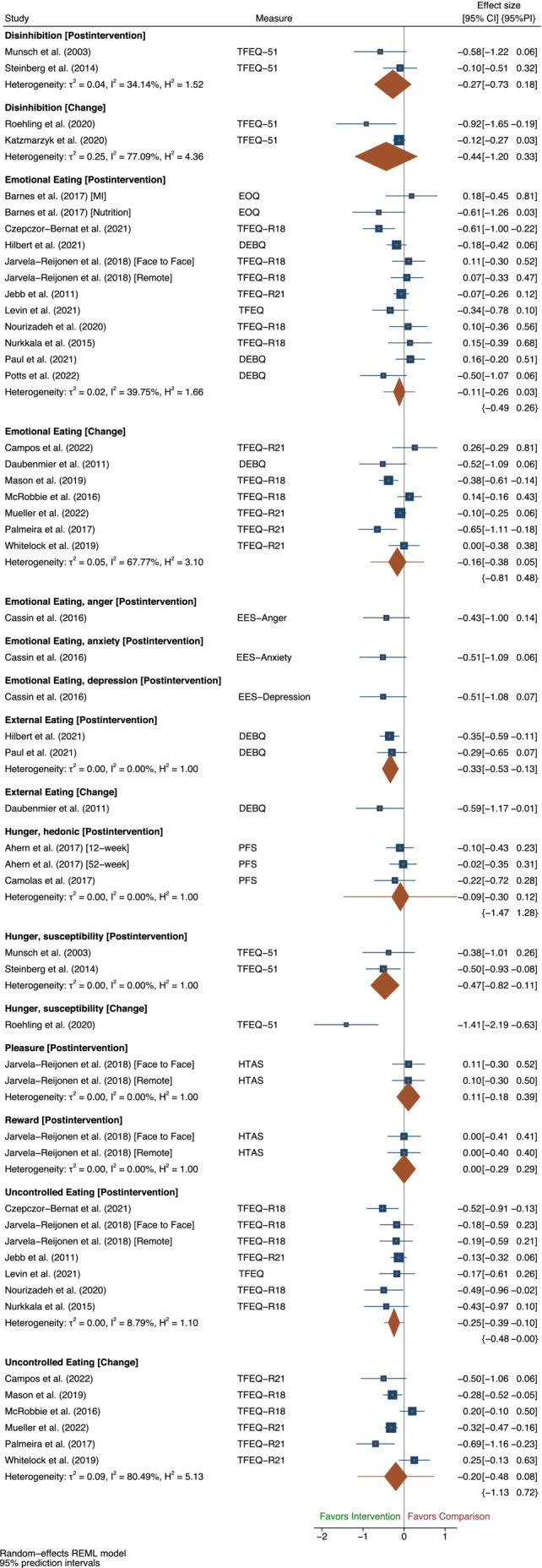
Outcomes where the desired intervention effect is a decrease in the trait at intervention end. *Note*: The size of blue squares representing each trial are proportional to the study weight. The width of the orange diamond representing overall effect size corresponds to the length of the confidence interval (CI), and its orange whiskers correspond to the length of the prediction interval (PI). The height of the diamond is irrelevant. *Abbreviations*: DEBQ, Dutch Eating Behaviour Questionnaire; EES, Emotional Eating Scale; EOQ, Emotional Overeating Questionnaire; HTAS, Health and Taste Attitude Scales; PFS, Power of Food Scale; TFEQ‐51, Three Factor Eating Questionnaire original 51 item version; TFEQ‐R18, Three Factor Eating Questionnaire revised 18 item version; TFEQ‐R21, Three Factor Eating Questionnaire revised 21 item version.

There was no evidence of an effect on emotional eating at intervention end (post intervention: SMD −0.11 [95% CI −0.26, 0.03], [95% PI −0.49, 0.26], *N* = 12, *I* ^2^ = 39.75%; change: SMD −0.16 [95% CI −0.38, 0.05], [95% PI −0.81, 0.48], *N* = 7, *I* ^2^ = 67.77%) (Figure 3). There was no evidence of an effect on emotional eating at follow‐up (post intervention: SMD 0.10 [95% CI −0.43, 0.64], *N* = 2, *I* ^2^ = 89.28%; change: SMD 0.10 [95% CI −0.14, 0.34], *N* = 2, *I* ^2^ = 0.00%) (Figure S3).

Findings remained similar after removing trials at high risk of bias at intervention end (*N* = 4) (post intervention: SMD −0.11 [95% CI −0.25, 0.04], [95% PI −0.48, 0.27], *N* = 10, *I* ^2^ = 40.45%; change: SMD −0.17 [95% CI −0.43, 0.10], [95% PI −1.03, 0.70], *N* = 5, *I* ^2^ = 67.85%) (Table S2) (Figure S5). Removing trials at high risk of bias at follow‐up (*N* = 1) resulted in just one remaining trial for change outcomes, finding no evidence of an effect on emotional eating (change: SMD 0.14 [95% CI −0.13, 0.41]) (Figure S7), and no trials were judged to be at high risk of bias for post intervention outcomes. No cluster RCTs reported emotional eating at either time point.

One trial used the Emotional Eating Scale (EES) to assess emotional eating in response to depression, anger, and anxiety. These outcomes were considered separate traits and were not combined in the meta‐analysis of overall emotional eating. This trial did not find evidence for an effect on any of the emotional eating outcomes (EES‐Anger: SMD −0.43 [95% CI −1.00, 0.14]; EES‐Anxiety: SMD −0.51 [95% CI −1.09, 0.06]; EES‐Depression: SMD −0.51 [95% CI −1.08, 0.07]) (Figure 3).

#### 3.4.3 Uncontrolled eating

Six trials contributing seven intervention arms reported post intervention outcomes for uncontrolled eating at intervention end, with evidence of a decrease in uncontrolled eating in favor of the intervention (SMD −0.25, [95% CI −0.39, −0.10]). Heterogeneity for this outcome was low (*I* ^2^ = 8.79%) and the prediction interval indicated effects from future studies to lie in the same direction [95% PI −0.48, −0.00]). Six further trials reported change outcomes for uncontrolled eating at end of intervention, with no evidence of an effect (change: SMD −0.20 [95% CI −0.48, 0.08]; [95% PI −1.13, 0.72]; *I* ^2^ = 80.49%) (Figure 3). Two trials reported change in uncontrolled eating at follow‐up, finding no evidence of an effect on uncontrolled eating (change: SMD 0.14 [95% CI −0.10, 0.39], *I* ^2^ = 0.00%) (Figure S3). No trials reported post intervention outcomes at follow‐up.

No trials reporting post intervention outcomes at intervention end were deemed at high risk of bias. Two trials deemed at high risk of bias were removed for sensitivity analyses of change outcomes at intervention end without changing conclusions (change: SMD −0.13 [95% CI −0.54, 0.28]; [95% PI −2.01, 1.75]; *I* ^2^ = 86.58%) (Table S2) (Figure S5). After removing trials at high risk of bias at follow‐up (*N* = 1), only one trial remained finding no evidence of an effect on uncontrolled eating (change: SMD 0.17 [95% CI −0.10, 0.43]) (Figure S7). No cluster RCTs reported data on uncontrolled eating.

#### 3.4.4 Disinhibition

There was no evidence of an effect of the intervention on disinhibition at intervention end (post intervention: SMD −0.27 [95% CI −0.73, 0.18], *N* = 2, *I* ^2^ = 34.14%; change: SMD −0.44 [95% CI −1.20, 0.33], *N* = 2, *I* ^2^ = 77.09%) (Figure 3). Only one trial assessed disinhibition at follow‐up, without finding evidence of an effect (post intervention: SMD −0.39 [95% CI −1.14, 0.36]) (Figure S3).

No contributing trials were deemed at high risk of bias. After removing cluster RCTs (*N* = 1) at intervention end, only one trial remained for change outcomes finding evidence of a decrease in disinhibition in favor of the intervention (change: SMD −0.92 [95% CI −1.65, −0.19]) (Figure S9). No cluster RCTs reported post intervention outcomes or outcomes at follow‐up.

#### 3.4.5 Intuitive/mindful eating

Three trials contributing four intervention arms reported post intervention outcomes for intuitive/mindful eating at intervention end, providing evidence of an effect in favor of the intervention (post intervention: SMD 0.23 [95% CI 0.04, 0.43], *N* = 4, *I* ^2^ = 0.00%). However, prediction intervals indicated a possibility of different effects being found in future studies ([95% PI −0.20, 0.66]) (Figure 2). No trials assessed intuitive eating at follow‐up.

No trials were cluster RCTs or deemed at high risk of bias.

#### 3.4.6 External eating

Two trials reported external eating at intervention end, providing evidence for a decrease in external eating in favor of the intervention (post intervention: SMD −0.33 [95% CI −0.53, −0.13]; *I* ^2^ = 0.00%). Only one trial reported change in external eating at the end of the intervention, so no meta‐analyses were performed. This trial also found evidence for a decrease in external eating (SMD −0.59 [95% CI −1.17, −0.01]) (Figure 3). Only one trial reported external eating scores at follow‐up, finding some evidence to suggest a decrease in external eating in favor of the intervention (SMD: −0.22 [95% CI −0.46, 0.02]) (Figure S3).

No contributing trials were cluster RCTs or deemed high risk of bias.

#### 3.4.7 Susceptibility to hunger

There was evidence from two trials of an intervention effect on susceptibility to hunger at intervention end (post intervention: SMD −0.47 [95% CI −0.82, −0.11], *N* = 2, *I* ^2^ = 0.00%). One trial reported change outcomes in susceptibility to hunger, finding effects in favor of the intervention (change: SMD −1.41 [95% CI −2.19. −0.63]) (Figure 3). One trial reported an effect on susceptibility to hunger at follow‐up and found no evidence of an effect (post intervention: SMD −0.39 [95% CI −1.14, 0.36]) (Figure S3).

No trials were cluster RCTs or deemed at high risk of bias.

#### 3.4.8 Hedonic hunger

Hedonic hunger was reported by two trials contributing three intervention arms at end of intervention, with no evidence of an effect of the intervention (post intervention: SMD −0.09 [95% CI −0.30, 0.12], [95% PI −1.47, 1.28]; *I* ^2^ = 00.00%) (Figure 3). Two intervention arms from one trial remained at follow‐up, with no evidence of an effect of the intervention on hedonic hunger (SMD −0.02 [95% CI −0.25, 0.22], *I* ^2^ = 0.00%) (Figure S3).

No trials were cluster RCTs or deemed at high risk of bias.

#### 3.4.9 Other EBTs

One trial with two intervention arms assessed contextual skills, internal regulation, using food as a reward, and pleasure.^54^ No evidence of an effect of the intervention was found for either of these outcomes at intervention end (contextual skills: post intervention: SMD 0.22 [95% CI −0.07, 0.50], internal regulation: post intervention: SMD −0.02 [95% CI −0.31. 0.26]; reward: post intervention: SMD 0.00 [95% CI −0.29, 0.29], pleasure: post intervention: SMD 0.11 [95% CI −0.18, 0.39]) (Figures 2 and 3). These outcomes were not reported at follow‐up, and the trial was neither deemed at high risk of bias nor was it cluster randomized.

### 3.5 Additional analyses

Subgroup analyses are presented in Figures S12–S59. Subgroup analyses were performed for the outcomes of emotional eating, intuitive/mindful eating (for some subgroups), restraint and uncontrolled eating. There was an insufficient number of trials to conduct subgroup analyses for contextual skills, disinhibition, external eating, hedonic hunger, susceptibility to hunger, internal regulation, using food as a reward, and pleasure of eating. Given the small number of trials contributing to each subgroup, no meta‐regression analyses were conducted.

Subgroup analyses by intervention type showed that while there was evidence of an effect for restraint in favor of the intervention in standard behavioral intervention arms [end of intervention: post intervention, change; follow‐up: post intervention], there was no evidence of an effect on restraint in third wave intervention arms [end of intervention: post intervention, change] (Figures S30 and S31 and Figure S42). There was evidence of an effect on uncontrolled eating in favor of the intervention in third wave intervention arms [end of intervention: post intervention] (Figures S48 and S49). All trials reporting intuitive/mindful eating outcomes were third wave behavioral interventions. Few second wave cognitive behavioral therapy‐based interventions were included across all outcomes, often preventing inclusion in subgroup analyses. Additionally, some interventions could not be allocated to the pre‐specified intervention type subgroups (i.e., motivational interviewing, cognitive remediation therapy).

Subgroup analyses by average baseline BMI showed that there was evidence of an effect in favor of the intervention on restraint [end of intervention: post intervention, change; follow‐up: post intervention] (Figures S40, S41, and S47), intuitive eating [end of intervention: post intervention] (Figure S29), emotional eating [end of intervention: change] (Figure S23) and uncontrolled eating [end of intervention: post intervention, change] (Figures S58 and S59) in interventions where the average baseline BMI was below 35. There was no evidence of an effect on restraint [end of intervention: post intervention, change; follow‐up: post intervention] (Figures S40, S41, and S47), emotional eating [end of intervention: post intervention, change] (Figures S22 and S23) and uncontrolled eating [end of intervention: change] (Figure S59) in interventions where the average baseline BMI was above 35. For intuitive/mindful eating and post intervention uncontrolled eating outcomes, all included trials had average BMIs below 35.

There was no clear pattern of evidence that intervention duration, delivery format, delivery mode, or comparison intensity explained heterogeneity for the outcomes of emotional eating, intuitive/mindful eating, restraint, or uncontrolled eating. For intuitive/mindful eating it seemed that there was evidence of an effect in favor of the intervention in individual and remote interventions whereas there was no evidence of an effect in group‐based or in person interventions.

Contour enhanced funnel plots are depicted in Figures S60–S68 and indicate a potential risk of publication bias for emotional eating, and restraint. The outcomes of disinhibition (*N* = 2), external eating (*N* = 2), hedonic hunger (*N* = 3), susceptibility to hunger (*N* = 2), intuitive eating (*N* = 4), and uncontrolled eating (*N* = 6) included less than 10 intervention arms, limiting the interpretation of funnel plots.^64^

## 4 DISCUSSION

This systematic review and meta‐analysis investigated the impact of behavioral weight management interventions on EBTs compared with no/minimal interventions or usual care. We found evidence of decreases in uncontrolled eating and increases in restraint following behavioral weight management interventions. Effects remained similar for restraint at the 12‐month follow‐up, with no evidence of an effect for uncontrolled eating. We found no evidence of an impact on emotional eating at intervention end or 12‐month follow‐up. We were unable to draw reliable conclusions for a variety of EBTs due to a small number of trials reporting data; these outcomes were disinhibition, external eating, hedonic hunger, susceptibility to hunger, intuitive/mindful eating, contextual skills, pleasure, reward, and internal regulation. Results from all outcomes should be interpreted with caution due to the low number of contributing trials, high heterogeneity, wide prediction intervals, and the risk of publication bias.

Findings on restraint and uncontrolled eating largely align with those from previous systematic reviews and meta‐analyses. For example, Chew et al.^24^ also found evidence of improvements in uncontrolled eating and restraint, and Jacob et al.^25^ also reported evidence for an increase in restraint. Only Lawlor et al.^23^ and Chew et al.^28^ did not find evidence of an effect on restraint. This could be explained by the different intervention types under study; Lawlor et al.^23^ focused specifically on third wave cognitive behavioral interventions, and Chew et al.^28^ on acceptance and commitment therapy‐based interventions, while this review took a broader approach to intervention type. This is supported by results from our subgroup analyses, where we found evidence of an effect of standard behavioral interventions for restraint, but we did not find evidence of an effect for restraint in third wave cognitive behavioral interventions. In contrast, for uncontrolled eating, we found evidence to suggest that third wave cognitive behavioral interventions reduced uncontrolled eating, while we did not find evidence of such an effect in standard behavioral interventions. This corresponds with Lawlor et al.'s^23^ review of third wave cognitive behavioral interventions, who also found evidence for a decrease in uncontrolled eating. Thus, while behavioral weight management interventions overall seemed to increase restriction of food intake and decrease overeating in response to external stimuli and feelings of hunger, the impact on those EBTs may depend on the type of intervention administered. More research is needed to continue to explore whether different intervention types have a greater impact on certain EBTs, in particular, whether restraint is best addressed in standard behavioral interventions and uncontrolled eating is best addressed in psychological interventions. This will aid the targeting of specific EBTs in future interventions, and could help match intervention types to individual's needs to maximize net benefit.

Findings on emotional eating were less conclusive compared with previous reviews. Although we found the direction of the effect for emotional eating to favor interventions, there was no evidence to conclude there was an effect. This is in line with Lawlor et al.^23^ and Chew et al.^28^ who also reported non‐significant reductions in emotional eating. Other reviews, including Jacob et al.,^25^ Chew et al.,^24^ Smith et al.,^27^ and DiSante et al.,^26^ found evidence of a small to medium effect on emotional eating in favor of the interventions. Again, the differences across review findings may be explained by the inclusion of different intervention types. For example, Jacob et al.^25^ focused on cognitive behavioral therapy based interventions, Chew et al.^24^ and Smith et al.^27^ on interventions specifically targeting emotional eating, and DiSante et al.^26^ on acceptance and commitment therapy based interventions. Thus, although behavioral weight management interventions in our review did not seem to significantly reduce overeating in response to negative emotions, this may differ according to intervention type and the intensity of included psychological or emotional eating specific content. Smith et al.^27^ explored the influence of intervention type on emotional eating and found that cognitive behavioral therapy‐based interventions were most effective. However, to our knowledge, no other review formally compared intervention types. Given that our findings suggest there is no evidence of improvements in emotional eating in behavioral weight management interventions more generally, future research is needed to explore how emotional eating can be better supported, and what kind of intervention content is required to change eating patterns in response to negative emotions.

In addition to intervention type, future research may explore other conditions impacting the effect of interventions on EBT outcomes. In subgroup analyses, we found that there was evidence of an effect on emotional eating, uncontrolled eating and restraint in trials where participants began with a BMI less than 35 kg/m^2^, but not in those with a baseline BMI greater than 35 kg/m^2^. Similarly, Chew et al.^24^ performed meta‐regression and concluded that baseline BMI was a significant moderator in study effects on uncontrolled eating. Thus, interventions may be more effective at addressing EBTs in people with lower baseline BMIs. However, more research is needed to strengthen our understanding in this area. Importantly, future research may wish to explore ways to ensure adequate support for all participants of weight management interventions, particularly those with higher baseline BMIs, to prevent exacerbating inequalities.

### 4.1 Strengths and limitations

To our knowledge, this is the first systematic review and meta‐analysis including (cluster) RCTs that synthesized a broad variety of EBTs and behavioral weight loss interventions that did not necessarily target specific EBTs. As such, this review offers the most comprehensive findings on the impact of adult behavioral weight management interventions on EBTs to date.

Our conclusions are impacted by limitations of the review and of included trials. The majority of included trials (*N* = 24/27; 88.88%)^21^, ^38^, ^39^, ^40^, ^41^, ^43^, ^44^, ^45^, ^46^, ^47^, ^49^, ^50^, ^51^, ^52^, ^53^, ^54^, ^55^, ^56^, ^57^, ^58^, ^59^, ^61^, ^62^, ^63^ were conducted in high income countries, and most samples were over 70% female (*N* = 19/27; 70.37%)^21^, ^38^, ^39^, ^40^, ^41^, ^43^, ^44^, ^45^, ^46^, ^49^, ^50^, ^52^, ^53^, ^54^, ^58^, ^59^, ^60^, ^62^, ^63^ or were female only samples (*N* = 6/27; 22.22%).^42^, ^47^, ^48^, ^51^, ^56^, ^57^ This limits the generalisability of our findings to other contexts. Additionally, funnel plots indicated a risk of publication bias. Because EBTs were often secondary outcomes of included trials, the power to estimate their effect may have been limited, potentially increasing the risk of a type‐I error.

While this review included 27 trials of adult behavioral weight management interventions, EBT outcomes were inconsistently measured and reported. Trials used a range of different EBT questionnaires and scoring approaches and reported the outcomes in different ways; this made it impossible to combine post intervention and change outcomes of the same trait in one meta‐analysis.^36^ As a result, a low number of trials contributed data to individual EBT outcomes, limiting our ability to draw conclusions, interpret findings, and compare outcomes. The low number of contributing trials also impacted our ability to examine heterogeneity, and we were unable to explore potential effect modifiers using meta‐regression. Additionally, the overlap of intervention characteristics (e.g., interventions of longer duration were more likely to be in‐person and in a group‐based format) made it challenging to unpick the effects of separate intervention characteristics on EBT outcomes. There may also be additional characteristics affecting EBT outcomes that were not explored in this review. For example, the level of expertise of those delivering the intervention was found to impact weight loss outcomes in an acceptance and commitment therapy based intervention, which could also have an impact on EBTs.^65^

### 4.2 Directions for future research

More research is needed to provide higher quality evidence on the impact of behavioral weight management interventions on EBTs across various contexts and populations. Future trials should aim to increase the assessment of EBTs and consistently report both post intervention and change outcomes to enable their integration in future reviews or make their anonymised raw data available in public data sharing repositories to be used for individual participant data meta‐analyses. This way we can produce higher quality evidence and examine potential sources of heterogeneity and effect modifiers, such as different intervention types and participants' baseline BMI.

## 5 CONCLUSION

This comprehensive systematic review and meta‐analysis of RCTs found that behavioral weight management interventions have small to moderate improvements for numerous EBTs at intervention end, including restraint, uncontrolled eating, intuitive eating, external eating and susceptibility to hunger. Evidence supports sustained improvements at 12 months for restraint. However, for many EBTs, the number of included trials was too low to form conclusions with confidence. Future research would benefit from more trials measuring and consistently reporting EBTs to strengthen our understanding of the impact of behavioral interventions on EBTs. This includes exploring which intervention types work best for targeting specific EBTs to enhance intervention effectiveness and allow for matching interventions to individuals' requirements. Additionally, research may explore ways to better address emotional eating in behavioral weight management interventions.

## AUTHOR CONTRIBUTIONS

Rebecca A. Jones developed the protocol and search strategy. Laura Kudlek led screening, data extraction, and risk of bias assessments, with duplicates performed by Struan Tait, Patricia Eustachio Colombo, Natasha Reid, Milindu Wickramarachchi, Aiswarya Lakshmi, Marie Spreckley, Julia Mueller, or Rebecca A. Jones. Laura Kudlek developed the data analysis plan with input from Rebecca A. Jones and Stephen J. Sharp and conducted analyses. Laura Kudlek led the interpretation of results and write‐up of the manuscript. All authors reviewed and approved the manuscript.

## CONFLICT OF INTEREST STATEMENT

AA is a member of the WW Scientific Advisory Board, for which payment is made to her institution, and is the Principal Investigator of two publicly funded trials (both included in this review) where the intervention was provided by WW at no cost. JM is a Trustee for the Association for the Study of Obesity (unpaid role).

## Supporting information


**Table S1.** Detailed characteristics of included trials.
**Figure S1.** Risk of Bias of included trials.
**Figure S2.** Outcomes where the desired intervention effect is an increase in the trait at follow‐up.
**Figure S3.** Outcomes where the desired intervention effect is a decrease in the trait at follow‐up.
**Table S2.** Prediction intervals for outcomes in which a decrease in the trait is the desired intervention effect at intervention end.
**Table S3.** Prediction intervals for outcomes in which an increase in the trait is the desired intervention effect at intervention end.
**Figure S4.** Outcomes where the desired intervention effect is an increase in the trait in trials with the risk of bias rated as “low” or “some concerns” at intervention end.
**Figure S5.** Outcomes where the desired intervention effect is a decrease in the trait in trials with the risk of bias rated as “low” or “some concerns” at intervention end.
**Table S4.** Prediction intervals for outcomes in which an increase in the trait is the desired intervention effect at 12 months follow‐up.
**Figure S6.** Outcomes where the desired intervention effect is an increase in the trait in trials with the risk of bias rated as “low” or “some concerns” at follow‐up.
**Figure S7.** Outcomes where the desired intervention effect is a decrease in the trait in trials with the risk of bias rated as “low” or “some concerns” at follow‐up.
**Table S5.** Prediction intervals for outcomes in which a decrease in the trait is the desired intervention effect at intervention end.
**Table S6.** Prediction intervals for outcomes in which an increase in the trait is the desired intervention effect at intervention end.
**Figure S8.** Outcomes where the desired intervention effect is an increase in the trait in randomised controlled trials at intervention end.
**Figure S9.** Outcomes where the desired intervention effect is a decrease in the trait in in randomised controlled trials at intervention end.
**Table S7.** Prediction intervals for outcomes in which an increase in the trait is the desired intervention effect at 12 months follow‐up.
**Figure S10.** Outcomes where the desired intervention effect is an increase in the trait in in randomised controlled trials at follow‐up.
**Figure S11.** Outcomes where the desired intervention effect is a decrease in the trait in in randomised controlled trials at follow‐up.
**Figure S12.** Post intervention emotional eating outcomes by intervention type at intervention end.
**Figure S13.** Change in emotional eating outcomes by intervention type at intervention end.
**Figure S14.** Post intervention emotional eating outcomes by intervention duration at intervention end.
**Figure S15.** Change in emotional eating outcomes by intervention duration at intervention end.
**Figure S16.** Post intervention emotional eating outcomes by intervention delivery format at intervention end.
**Figure S17.** Change in emotional eating outcomes by intervention delivery format at intervention end.
**Figure S18.** Post intervention emotional eating outcomes by intervention delivery mode at intervention end.
**Figure S19.** Change in emotional eating outcomes by intervention delivery mode at intervention end.
**Figure S20.** Post intervention emotional eating outcomes by comparison intensity at intervention end.
**Figure S21.** Change in emotional eating outcomes by comparison intensity at intervention end.
**Figure S22.** Post intervention emotional eating outcomes by baseline BMI at intervention end.
**Figure S23.** Change in emotional eating outcomes by baseline BMI at intervention end.
**Figure S24.** Post intervention intuitive/ mindful eating outcomes by intervention type at intervention end.
**Figure S25.** Post intervention intuitive/ mindful eating outcomes by intervention duration at intervention end.
**Figure S26.** Post intervention intuitive/ mindful eating outcomes by intervention delivery format at intervention end.
**Figure S27.** Post intervention intuitive/ mindful eating outcomes by intervention delivery mode at intervention end.
**Figure S28.** Post intervention intuitive/mindful eating outcomes by comparison intensity at intervention end.
**Figure S29.** Post intervention intuitive/ mindful eating outcomes by baseline BMI at intervention end.
**Figure S30.** Post intervention restraint outcomes by intervention type at intervention end.
**Figure S31.** Change in restraint outcomes by intervention type at intervention end.
**Figure S32.** Post intervention restraint outcomes by intervention duration at intervention end.
**Figure S33.** Change in restraint outcomes by intervention duration at intervention end.
**Figure S34.** Post intervention restraint outcomes by intervention delivery format at intervention end.
**Figure S35.** Change in restraint outcomes by intervention delivery format at intervention end.
**Figure S36.** Post intervention restraint outcomes by intervention delivery mode at intervention end.
**Figure S37.** Change in restraint outcomes by intervention delivery mode at intervention end.
**Figure S38.** Post intervention restraint outcomes by comparison intensity at intervention end.
**Figure S39.** Change in restraint outcomes by comparison intensity at intervention end.
**Figure S40.** Post intervention restraint outcomes by baseline BMI at intervention end.
**Figure S41.** Change in restraint outcomes by baseline BMI at intervention end.
**Figure S42.** Post intervention restraint outcomes by intervention type at follow‐up.
**Figure S43.** Post intervention restraint outcomes by intervention duration at follow‐up.
**Figure S44.** Post intervention restraint outcomes by intervention delivery format at follow‐up.
**Figure S45.** Post intervention restraint outcomes by intervention delivery mode at follow‐up.
**Figure S46.** Post intervention restraint outcomes by comparison intensity at follow‐up.
**Figure S47.** Post intervention restraint outcomes by baseline BMI at follow‐up.
**Figure S48.** Post intervention uncontrolled eating outcomes by intervention type at intervention end.
**Figure S49.** Change in uncontrolled eating outcomes by intervention type at intervention end.
**Figure S50.** Post intervention uncontrolled eating outcomes by intervention duration at intervention end.
**Figure S51.** Change in uncontrolled eating outcomes by intervention duration at intervention end.
**Figure S52.** Post intervention uncontrolled eating outcomes by intervention delivery format at intervention end.
**Figure S53.** Change in uncontrolled eating outcomes by intervention delivery format at intervention end.
**Figure S54.** Post intervention uncontrolled eating outcomes by intervention delivery mode at intervention end.
**Figure S55.** Change in uncontrolled eating outcomes by intervention delivery mode at intervention end.
**Figure S56.** Post intervention uncontrolled eating outcomes by comparison intensity at intervention end.
**Figure S57.** Change in uncontrolled eating outcomes by comparison intensity at intervention end.
**Figure S58.** Post intervention uncontrolled eating outcomes by baseline BMI at intervention end.
**Figure S59.** Change in uncontrolled eating outcomes by baseline BMI at intervention end.
**Figure S60.** Contour enhanced funnel plots for disinhibition.
**Figure S61.** Contour enhanced funnel plots for emotional eating.
**Figure S62.** Contour enhanced funnel plots for external eating.
**Figure S63.** Contour enhanced funnel plots for hedonic hunger.
**Figure S64.** Contour enhanced funnel plots for susceptibility to hunger.
**Figure S65.** Contour enhanced funnel plots for intuitive/mindful eating.
**Figure S66.** Contour enhanced funnel plots for restraint.
**Figure S67.** Contour enhanced funnel plots for uncontrolled eating.
**Figure S68.** Contour enhanced funnel plots for contextual skills, internal regulation, pleasure of eating and eating as a reward at end of intervention.
