# Supplementary material for: The impact of behavioral weight management interventions on eating behavior traits in adults with overweight or obesity: A systematic review and meta‐analysis
Source: Obes Rev. 2024 Nov 21;26(4):e13871. doi: 10.1111/obr.13871 (PMC11884964; doi:10.1111/obr.13871)
Supplement: Supplementary file 1 — Table S1. Detailed characteristics of included trials. Figure S1. Risk of Bias of included trials. Figure S2. Outcomes where the desired intervention effect is an increase in the trait at follow‐up. Figure S3. Outcomes where the desired intervention effect is a decrease in the trait at follow‐up. Table S2. Prediction intervals for outcomes in which a decrease in the trait is the desired intervention effect at intervention end. Table S3. Prediction intervals for outcomes in which an increase in the trait is the desired intervention effect at intervention end. Figure S4. Outcomes where the desired intervention effect is an increase in the trait in trials with the risk of bias rated as “low” or “some concerns” at intervention end. Figure S5. Outcomes where the desired intervention effect is a decrease in the trait in trials with the risk of bias rated as “low” or “some concerns” at intervention end. Table S4. Prediction intervals for outcomes in which an increase in the trait is the desired intervention effect at 12 months follow‐up. Figure S6. Outcomes where the desired intervention effect is an increase in the trait in trials with the risk of bias rated as “low” or “some concerns” at follow‐up. Figure S7. Outcomes where the desired intervention effect is a decrease in the trait in trials with the risk of bias rated as “low” or “some concerns” at follow‐up. Table S5. Prediction intervals for outcomes in which a decrease in the trait is the desired intervention effect at intervention end. Table S6. Prediction intervals for outcomes in which an increase in the trait is the desired intervention effect at intervention end. Figure S8. Outcomes where the desired intervention effect is an increase in the trait in randomised controlled trials at intervention end. Figure S9. Outcomes where the desired intervention effect is a decrease in the trait in in randomised controlled trials at intervention end. Table S7. Prediction intervals for outcomes in which an increase in the tra [file OBR-26-e13871-s001.pdf]

The Impact of Behavioural Weight Management Interventions on Eating Behaviour Traits in Adults with Overweight or Obesity: A Systematic Review and Meta-analysis

Laura Kudlek*[a], Patricia Eustacio Colombo [a], Amy Ahern [a], Struan Tait [a], Natasha Reid [b], Milindu Wickramarachchi [b], Aiswarya Lakshmi [b], Stephen J. Sharp [a], Marie Spreckley [a], Julia Mueller [a], Rebecca A. Jones [a]

[SUPPLEMENTARY MATERIAL]

[a] MRC Epidemiology Unit, School of Clinical Medicine, University of Cambridge, Cambridge, UK

[b] School of Clinical Medicine, University of Cambridge, Cambridge, UK

*Corresponding author, laura.kudlek@mrc-epid.cam.ac.uk

Content

1.0 Search Strategy 4
2.0 Characteristics of included trials 5
3.0 Risk of Bias 30
4.0 Intervention effects on EBTs at Follow-up 31
5.0 Sensitivity Analyses 33
  5.1 Risk of Bias 33
    5.1.1 Sensitivity analyses for risk of bias at end of intervention 33
    5.1.2 Sensitivity analyses for risk of bias at follow-up 37
  5.2 Study Design 39
    5.2.1 Sensitivity analyses for study design at end of intervention 39
    5.2.2 Sensitivity analyses for study design at follow-up 43
6.0 Subgroup Analyses 45
  6.1 Disinhibition 45
  6.2 Emotional Eating 45
    6.2.1 Impact on emotional eating by subgroups at end of intervention 45
    6.2.2 Impact on emotional eating by subgroups at follow-up 53
  6.3 External Eating 53
  6.4 Hunger, hedonic 53
  6.5 Hunger, susceptibility 53
  6.6 Intuitive/ Mindful Eating 53
    6.6.1 Impact on intuitive/ mindful eating by subgroups at end of intervention 53
    6.6.2 Impact on intuitive/ mindful eating by subgroups at follow-up 56
  6.7 Restraint 57
    6.7.1 Impact on restraint by subgroups at end of intervention 57
    6.7.2 Impact on restraint by subgroups at follow-up 69
  6.8 Uncontrolled Eating 74
    6.8.1 Impact on uncontrolled eating by subgroups at intervention end 74
    6.8.2 Impact on uncontrolled eating by subgroups at follow-up 82
  6.9 Other EBTs 82
7.0 Publication Bias 83
  7.1 Disinhibition 83
  7.2 Emotional Eating 84
  7.3 External Eating 85

7.4 Hunger, hedonic 86
7.5 Hunger, susceptibility 87
7.6 Intuitive/ Mindful Eating 88
7.7 Restraint 89
7.8 Uncontrolled Eating 90
7.9 Other EBTs 91
8.0 References 92

1.0 Search Strategy

Search Strategy as applied to MEDLINE

(1) Participants	1.	exp Overweight/ or exp Obesity/ or (adipos* or obes* or over?weight).tw
(2) Weight management intervention	2.	exp Body Weight/ or exp Life Style/ or exp Physical Activity/ or exp Obesity Management/ or exp Exercise/ or exp Diet/ or exp Behavior Therapy/ or exp Health Education/ or ((weight adj3 (body or chang* or los* or maint* or manage* or control* or reduc*)) or (body?mass?index or bmi) or (body adj3 mass) or life?style or ((obes* or weight*) adj3 (intervention or program* or treat* or trial)) or (behavio?r* or psych*)).tw
(3) Eating behaviour trait outcomes	3.	exp Feeding Behavior/ or exp Drive/ or exp Satiation/ or exp Appetite/ or exp Appetite Regulation or ((eating or appetit* or food or satiat*) adj3 (behav* or trait* or phenotype* or regulat* or disturb* or respons* or sensitiv* or uncontrol* or emotion* or comfort* or external)) or (hedonic adj3 (eat* or respons* or hunger or appetite)) or ((cognitive or diet*) adj3 (restrain* or restrict*)) or (disinhibit*) or (hunger*) or (craving*).tw
(4) Study design	4.	exp Randomized Controlled Trials as Topic/ or randomized controlled trial.pt or controlled clinical trial.mp or randomi?ed.mp or randomly.mp or trial.mp
	5.	1 AND 2 AND 3 AND 4

2.0 Characteristics of included trials

Table S1 Detailed characteristics of included trials

Trial	Methods	Participant characteristics	Intervention and comparators	Outcomes and time points
Ahern et al. (2017) [1–3]	Study design:<br>Three-arm RCT (combined for meta-analyses)<br><br>Country:<br>United Kingdom<br><br>Setting:<br>Commercial (Weight Watchers)<br><br>Eligibility:<br>Adults (≥18y) with a BMI ≥ 28 kg/m^2	Sex: 68% female<br><br>Age in years[1]:<br>Control arm: 51.91 (14.07)<br>12 week arm: 53.60 (12.27)<br>52 week arm: 53.29 (13.98)<br><br>BMI (kg/m^2)[1]:<br>Control arm: 34.43 (4.63)<br>12 week arm: 34.68 (5.39)<br>52 week arm: 34.45 (5.05)<br><br>Ethnicity:<br>White: 91%<br>Black: 2%<br>Asian: 2%<br>Mixed or multiple: 1%<br>Other: 1%<br>Missing or prefer not to say: 3%<br><br>Education:<br>None: 4.6%<br>GCSE/A-Level/equivalent: 49%<br>University degree or higher equivalent: 35.8%<br>Missing: 10.2%	Interventions: (1) 12-week behavioural weight loss programme (N = 528) or (2) 52-week behavioural weight loss programme (N = 528)<br><br>• **Content:** Vouchers covering the cost to attend community-based Weight Watchers (WW) meetings and digital tools. WW consists of a food points-based system and behavior change techniques.<br>• **Intensity and delivery:** either 12 or 52 weekly 1 hour long group based face-to-face meetings led by a non-specialist individual who has personal experience of successful weight management<br><br>Comparator (N = 211):<br><br>• **Type:** Usual Care<br>• **Content:** Provision of a 32-page self-help weight loss pamphlet	Outcome(s) and Measure(s):<br>• Restraint (TFEQ)<br>• Hedonic Hunger (PFS)<br><br>Assessment time points:<br>• 3 months<br>• 12 months<br>• 24 months

		Income:<br>Under £20,000: 15.15%<br>£20-£49,999: 32.75%<br>£50,000+: 17%<br>Prefer not to say or missing: 18.8%		
Barnes et al. (2017) [4]	Study design:<br>Three-arm RCT<br><br>Country:<br>USA<br><br>Setting:<br>Clinical<br><br>Eligibility:<br>Adults (≤ 65y) with a BMI between 25 and 55kg/m²	Sex: 76.4% female<br><br>Age in years[1]:<br>47.91 (10.46)<br><br>BMI (kg/m²)[1]:<br>35.19 (6.87)<br><br>Ethnicity:<br>White: 65.2%<br>African American: 20.2%<br>Bi/multiracial: 5.6%<br>White, Hispanic: 4.5%<br>Bi/multiracial Hispanic: 4.5%<br><br>Education:<br>Control arm:<br>≤ High school diploma: 13%<br>Some college: 37%<br>≥ college: 50%<br><br>Motivational interviewing arm:<br>≤ High school diploma: 10%<br>Some college: 30%<br>≥ college: 60%<br><br>Nutrition arm:	Interventions: (1) Motivational interviewing + internet (N = 30) or (2) Nutrition Psychoeducation + internet (N = 29)<br><br>● **Content:** (1) Motivational Interviewing + LEARN manual (free weight loss website including nutritional advice and physical activity recommendations, and self-monitoring of diet and weight loss goals) or (2) basic nutritional information based on recommendations provided from the American Heart Goundation and United States Department of Agriculture + LEARN manual<br>● **Intensity and delivery:** One in person 60 minute group session followed by four sessions (2 in person group sessions and 2 phone sessions) of 20 minute duration. Sessions were delivered over the course of 12 weeks.<br><br>Comparator (N = 30):	Outcome(s) and Measure(s):<br>● Restraint (EDE-R)<br>● Emotional Eating (EOQ)<br><br>Assessment time points:<br>● 12 weeks<br>● 6 months

		≤ High school diploma: 14%<br>Some college: 21%<br>≥ college: 66%<br><br>Income:<br>n/a	• **Type:** Usual Care<br>**Content:** Brief initial instructions to continue working with primary care providers regarding weight concerns	
Camolas et al. (2017) [5]	Study design:<br>Two-arm RCT<br><br>Country:<br>Portugal<br><br>Setting:<br>Clinical<br><br>Eligibility:<br>Adult patients with class 2 or 3 obesity (defined by national norms for obesity surgery) referred to the obesity treatment of a Portuguese hospital	Sex: 80.85% female<br><br>Age in years[1]:<br>Comparison arm: 43.53 (13.92)<br>Intervention arm: 46.31 (13.65)<br><br>BMI (kg/m^2)[1]:<br>Comparison arm: 43.45 (7.04)<br>Intervention arm: 42.81 (4.96)	Intervention: "INDIVIDUO" (N = 45)<br><br>• **Content:** Personalised guidance based on principles from the transtheoretical model of health behavior change, self-determination theory, and motivational interviewing<br>• **Intensity and delivery:** 4 bi-monthly consultations delivered by clinical nutritionists that received training on the INDIVIDUO principles<br><br>Comparator (N = 49):<br><br>• **Type:** Minimal<br>• **Content:** health literacy promotion. Discussed content of leaflets including weight reduction strategies and a standard hypocaloric dietary plan<br>• **Intensity and delivery:** 2 individual, in-person meetings with a nutritionist and provision of 2 leaflets	Outcome(s) and Measure(s):<br><br>• Hedonic Hunger (PFS)<br><br>Assessment time points:<br><br>• 24 weeks
Campos et al. (2022) [6]	Study design:	Sex: 84.5% female<br><br>Age in years[1]: 40.5 (9.1)	Intervention: (1) Intuitive Eating Group (IEG) (N = 28) or (2) Intuitive Eating Dietary Guidelines Group (IEDG) (N = 27)	Outcome(s) and Measure(s):<br><br>• Restraint (TFEQ-R21)

	Three-arm RCT (combined for meta-analyses)<br><br>Country:<br>Brazil<br><br>Setting:<br>Clinical<br><br>Eligibility:<br>Adults (≥20y) awaiting bariatric surgery with a BMI ≥ 30kg/m²	BMI (kg/m²)[1]: 48.3 (7.4)<br><br>Ethnicity: n/a<br><br>Education:<br>Primary school: 32.8%<br>High School: 43.1%<br>Graduation: 24.1%<br><br>Income[1]: 621 (401.7) reais per capita	• **Content:** Intuitive Eating based on a book written by Evelyn Tribole and Elyse Resch. The IEDG group received additional content based on the Dietary Guidelines for the Brazilian Population (2nd edition).<br>• **Intensity and delivery:** 7 bi-weekly 1 hour long group based face to face sessions (plus one reinforcement session after a month) led by a nutritionist and supported by a psychologist during the final session + printed materials for home based exercises<br><br>Comparator (N = 27):<br><br>• **Type:** Usual care (standard pre-operative care for bariatric surgery)<br>• **Content:** Nutritional assistance including a food plan<br>• **Intensity and delivery:** 3 monthly individual face to face visits	• Uncontrolled Eating (TFEQ-R21)<br>• Emotional Eating (TFEQ-R21)<br><br>Assessment time points:<br><br>• 3 months<br>• 6 months
Cassin et al. (2016)[7]	Study design:<br>Two-arm RCT<br><br>Country:<br>Canada<br><br>Setting:<br>Clinical, remote	Sex: 83% female<br><br>Age in years[1]: 45.5 (8.9)<br><br>BMI (kg/m²)[1]: 53.1 (12)<br><br>Ethnicity: 91.5% White	Intervention: Telephone CBT (N = 23)<br><br>• **Content:** Cognitive Behavioural Therapy (CBT)<br>• **Intensity and delivery:** 6 weekly telephone based individual sessions of approximately 55 min duration +	Outcome(s) and Measure(s):<br><br>• Emotional Eating Anxiety, Anger and Depressions (EES)<br><br>Assessment time points:

	Eligibility:<br>Bariatric surgery candidates	Education:<br>Some College/ University: 31.9%<br>Completed College/ University degree: 48.9%<br><br>Income: n/a	homework to be completed between sessions<br><br>Comparator (N = 24):<br><br>● **Type:** Usual care (standard pre-operative care for bariatric surgery)<br>● **Content:** Education on bariatric surgery and access to support group<br>● **Intensity and delivery:** individual face to face clinic visits, optional monthly support group meetings	● 7 weeks
Czepczor-Bernat et al. (2021) [8]	Study design:<br>Three-arm RCT (combined for meta-analyses)<br><br>Country:<br>Poland<br><br>Setting:<br>Remote<br><br>Eligibility:<br>Pre-menopausal women (≤ 48y) with a BMI ≥ 25kg/m^2	Sex: 100% female<br><br>Age in years[1]:<br>Comparison: 31.03 (7.84)<br>Intervention 1: 32.67 (8.25)<br>Intervention 2: 33 (6.91)<br><br>BMI (kg/m^2)[1]:<br>Comparison: 31.5 (2.43)<br>Intervention 1: 29.61 (4.17)<br>Intervention 2: 30.59 (3.97)<br><br>Ethnicity: n/a<br><br>Education: n/a<br><br>Income: n/a	Intervention: (1) Theoretically consistent (N = 43) or (2) Eclectic (N = 46)<br><br>● **Content:** Both intervention groups shared two modules on eating behaviour (based on mindfulness eating training) and on body image (based on Cash's prevention of body image disturbances). The third module on emotional functioning was based on Emotional Schema Therapy for the first intervention group (theoretically consistent) and on a combination of different approaches, including acceptance and commitment therapy (ACT), dialectical behavioural therapy, and compassion focussed therapy for the second intervention group (eclectic).	Outcome(s) and Measure(s):<br><br>● Uncontrolled Eating (TFEQ-R18)<br>● Emotional Eating (TFEQ-R18)<br>● Mindful Eating (MES)<br><br>Assessment time points:<br><br>● 16 days<br>● 76 days

			• **Intensity and delivery:** 3 online modules completed individually over 15 days<br><br>Comparator (N = 40):<br><br>• **Type:** Waitlist control<br>• **Content:** n/a<br>• **Intensity and delivery:** n/a	
Daubenmier et al. (2011) [9]	Study design:<br>Two-arm RCT<br><br>Country:<br>United States<br><br>Setting:<br>Clinical<br><br>Eligibility:<br>Pre-menopausal women with a BMI ≥ 25kg/m^2 and ≤ 40kg/m^2	Sex: 100% female<br><br>Age in years[1]:<br>Comparison: 41.39 (6.7)<br>Intervention: 40.42 (8)<br><br>BMI (kg/m^2)[1]:<br>Comparison: 30.77 (SD 4.8)<br>Intervention: 31.40 (SD 4.7)<br><br>Ethnicity:<br>White: 62%<br>Hispanic/Latino: 15%<br>Asian/Pacific Islander: 15%<br>Other: 9%<br><br>Education: n/a<br><br>Income: n/a	Intervention: Mindfulness (N = 24)<br><br>• **Content:** Mindfulness intervention drawing on components from Mindfulness-Based Stress Reduction (MBSR), Mindfulness-Based Cognitive Therapy (MBCT) and Mindfulness-Based Eating Awareness Training (MB-EAT).<br>• **Intensity and delivery:** 9 weekly 2.5 hour long group based face to face classes and 1x 7-hour silent day of guided meditation after class 6 + home based exercises<br><br>Comparator (N = 23):<br><br>• **Type:** Minimal/ Waitlist<br>• **Content:** both groups participated in a nutrition and exercise information session which did not include information on mindfulness<br>• **Intensity and delivery:** 1 group face to face 2 hour long session	Outcome(s) and Measure(s):<br><br>• Restraint (DEBQ)<br>• External Eating (DEBQ)<br>• Emotional Eating (DEBQ)<br><br>Assessment time points:<br><br>• 9 weeks

Hilbert et al. (2021) [10]	Study design:<br>Two-arm RCT<br><br>Country:<br>Germany<br><br>Setting:<br>Clinical<br><br>Eligibility:<br>Adults (≥18y) with a BMI ≥ 35kg/m^2	Sex: 68.9% female<br><br>Age in years[1]: 44.5 (12.8)<br><br>BMI (kg/m^2)[1]: 45.6 (6.9)<br><br>Ethnicity: 98.5% German<br><br>Education: 28.7% education ≥12 years<br><br>Income: n/a	Intervention: CRT (N = 134)<br><br>● **Content:** Usual care + Cognitive Remediation Therapy (CRT) including strategies on meta-cognition and applying new thinking strategies<br>● **Intensity and delivery:** Usual care + 27 tasks in 8 weekly face to face group sessions (6 to 10 participants per group) of 2h duration run by two master's level female clinical psychologists with specific training in CRT + home based exercises.<br><br>Comparator (N = 136):<br><br>● **Type:** Usual care<br>● **Content:** Nutritional counselling, guided exercise, behavioural therapy<br>● **Intensity and delivery:** Depending on participants health insurance, they received high or low intensity treatments.<br>● High intensity treatment includes: 6 to 12 group and up to 6 individual nutritional counselling sessions, 40 to 48 group exercise sessions, 10 to 12 group and up to 6 individual behaviour therapy sessions.<br>● Low intensity treatment includes: 8 group and 3 individual nutritional counselling sessions, 50 group exercise sessions	Outcome(s) and Measure(s):<br><br>● Restraint (DEBQ)<br>● External Eating (DEBQ)<br>● Emotional Eating (DEBQ)<br><br>Assessment time points:<br><br>● 2 months<br>● 6 months<br>● 12 months

			● Sessions were conducted by registered dietitians, bachelor's or master's level sports scientists, and masters level clinical psychologists (for high intensity treatment)	
Jarvela-Reijonen et al. (2018) [11–13]	Study design:<br>Three-arm RCT (comparison arm split for meta-analyses)<br><br>Country:<br>Finland<br><br>Setting:<br>Community, remote<br><br>Eligibility:<br>Adults (≥25y ≤60y) with symptoms of psychological distress (≥3/12 on the GHQ-12) and a BMI between 27 and 35kg/m^2	Sex: 85% female<br><br>Age in years[1]: 49.5 (7.4)<br><br>BMI (kg/m^2)[1]: 31.3 (2.9)<br><br>Ethnicity: n/a<br><br>Education: n/a<br><br>Income: n/a	Intervention: (1) Face to face (N = 70) or (2) remote (78)<br><br>● **Content:** Acceptance and Commitment Therapy (ACT)<br>● **Intensity and delivery:**<br>○ (1) Face to Face: 6 group sessions with 6 to 12participants of 90 mins duration, led by a psychologist + homework and a printed workbook<br>○ (2) Remote: One initial face to face group session to explain principles of ACT and the pre-installed Oiva mobile app. The app contained 46 exercises of approx. 1-3 mins. Which participants could complete as many times they wanted in any order<br><br>Comparator (N = 71):<br><br>● **Type:** Waitlist<br>● **Content:** n/a	Outcome(s) and Measure(s):<br><br>● Restraint (TFEQ-R18)<br>● Uncontrolled Eating (TFEQ-R18)<br>● Emotional Eating (TFEQ-R18)<br>● Intuitive eating (IES)<br>● Internal Regulation (ecSatter Inventory)<br>● Contextual Skills (ecSatter Inventory)<br>● Using food as a reward (HTAS)<br>● Pleasure (HTAS)<br><br>Assessment time points:<br><br>● 10 weeks<br>● 36 weeks

			● **Intensity and delivery:** n/a	
Jebb et al. (2011) [14]	Study design:<br>Two-arm RCT<br><br>Country:<br>United Kingdom, Australia, and Germany<br><br>Setting:<br>Clinical, commercial<br><br>Eligibility:<br>Adults (≥18y) with a BMI between 27 and 35kg/m^2 and at least one additional risk factor for obesity related disease	Sex: 86.5% female<br><br>Age in years[1]:<br>Comparison: 48.2 (12.2)<br>Intervention: 46.5 (13.5)<br><br>BMI (kg/m^2)[1]:<br>Comparison: 31.3 (2.6)<br>Intervention: 31.5 (2.6)<br><br>Ethnicity: n/a<br><br>Education: n/a<br><br>Income: n/a	Intervention: (N = 377)<br><br>● **Content:** Free access to attend community-based Weight Watchers (WW) meetings and digital tools. WW consists of a food points-based system and behavior change techniques.<br>● **Intensity and delivery:** 52 weekly 1 hour long group based face-to-face meetings led by a non-specialist individual who has personal experience of successful weight management<br><br>● **Content:** X<br>● **Intensity and delivery:** X<br><br>Comparator (N = 395):<br><br>● **Type:** Usual care<br>● **Content:** brief advice<br>● **Intensity and delivery:** weight loss advice from a primary care professional at their local general practitioner (GP) practice	Outcome(s) and Measure(s):<br><br>● Restraint (TFEQ-R21)<br>● Uncontrolled Eating (TFEQ-R21)<br>● Emotional Eating (TFEQ-R21)<br><br>Assessment time points:<br><br>● 52 weeks
Katzmarzyk et al. (2020) [15]	Study design:<br>Two-arm cluster RCT<br><br>Country:<br>United States	Sex: 84.4%<br><br>Age in years[1]: 49.4 (13.1)<br><br>BMI (kg/m^2)[1]: 37.2 (4.7)<br><br>Ethnicity:	Intervention: ILI (N Clusters = 9 clinics; N participants = 452)<br><br>● **Content:** Pragmatic, intensive lifestyle program, which was consistent with the 2013 recommendations for the	Outcome(s) and Measure(s):<br><br>● Restraint (TFEQ-51)<br>● Disinhibition (TFEQ-51)

	Setting:<br>Clinical, remote<br><br>Eligibility:<br>Adults (≥20y ≤75y) with a BMI between 30 and 50kg/m^2	Black: 67.2%<br>White: 25.9%<br>Other: 6.8%<br><br>Education: n/a<br><br>Income:<br>Total household income <40.000$: 64.1%<br>Total household income >40.000$: 33.8%<br>Missing: 2.1 %	management of overweight and obesity set out by the American Heart Association, American College of Cardiology, and The Obesity Society. Included education on nutrition and physical activity as well as provision of meal replacements.<br>● **Intensity and delivery:** 24 month total duration, with weekly sessions in the first 6 months (16 individual face to face and 6 via telephone), followed by sessions that were held at least monthly. Sessions were delivered by trained health coaches embedded within primary care clinics.<br><br>Comparator (N = N Clusters = 9 clinics; N participants = 351):<br><br>● **Type:** Usual Care<br>● **Content:** the care routinely delivered by the clinic for the duration of the trial<br>● **Intensity and delivery:** 6 newsletters that covered numerous topics such as sitting and health, goal setting, memory health, self-care, sleep hygiene, and smoking cessation	Assessment time points:<br><br>● 6 months<br>● 12 months
Keraenen et al. (2009) [16]	Study design:<br>Two-arm RCT<br><br>Country:<br>Finland	Sex: 72% female<br><br>Age in years[1]: 49 (9)<br><br>BMI (kg/m^2)[1]: 35 (5)	Intervention: (N = 97)<br><br>● **Content:** Dietary counselling, including topics such as motivation, planning,	Outcome(s) and Measure(s):<br><br>● Restraint (TFEQ)<br>● Uncontrolled Eating (TFEQ)

	Setting:<br>Clinical<br><br>Eligibility:<br>Adults (≥18y ≤65y) with a BMI ≥27kg/m^2	Ethnicity: n/a<br><br>Education:<br>Basic education: 12%<br>Vocational training: 22%<br>College: 42%<br>University or polytechnics: 23%<br><br>Income: n/a	cooking skills, recognising emotional eating, evaluating dietary changes, barriers, maintaining weight loss etc.<br>● **Intensity and delivery:** 10 face to face sessions (both group and individual) every second week over a time period of 20 weeks delivered by a clinical nutritionist + homework<br><br>Comparator (N = 98):<br><br>● **Type:** Minimal<br>● **Content:** Dietary counselling, similar to the two introductory sessions of the intervention group<br>● **Intensity and delivery:** 2 individual visits at a 2 week interval delivered by a nurse specialised in obesity	● Emotional Eating (TFEQ)<br><br>Assessment time points:<br><br>● 18 months
Levin et al. (2021) [17]	Study design:<br>Two-arm RCT<br><br>Country:<br>United States<br><br>Setting:<br>Remote<br><br>Eligibility:<br>Adults (≥18y) with a BMI ≥25kg/m^2	Sex: 82.3% female<br><br>Age in years[1]: 39.56 (12.12)<br><br>BMI (kg/m^2)[1]: 33.78 (5.69)<br><br>Ethnicity: 92.4% White<br><br>Education: n/a<br><br>Income:<br>Intervention median household income: $60,000-79,000	Intervention: ACT on health (N = 39)<br><br>● **Content:** ACT with behavioural strategies<br>● **Intensity and delivery:** online guided self-help programme delivered through an online learning management system (Canvas) monitored by a doctoral student in clinical/ counselling psychology. Included 8 weekly sessions and 5 to 10 minute weekly coaching calls or emails<br><br>Comparator (N = 40):	Outcome(s) and Measure(s):<br><br>● Restraint (TFEQ)<br>● Uncontrolled Eating (TFEQ)<br>● Emotional Eating (TFEQ)<br><br>Assessment time points:<br><br>● 8 weeks<br>● 16 weeks

		Control median household income: $60,000-79,000	● **Type:** Waitlist<br>● **Content:** n/a<br>● **Intensity and delivery:** n/a	
Mason et al. (2019) [18]	Study design:<br>Four-arm RCT (combined for meta-analyses)<br><br>Country:<br>United States<br><br>Setting:<br>Clinical, remote<br><br>Eligibility:<br>Post-menopausal women (≥50y ≤75y) with a BMI ≥ 25kg/m^2	Sex: 100% female<br><br>Age in years[1]: 58 (5)<br><br>BMI (kg/m^2)[1]: 30.9 (4)<br><br>Ethnicity:<br>Non-Hispanic White: 85%<br>Non-Hispanic Black: 8%<br><br>Education: 65.4% college graduate<br><br>Income: n/a	Intervention: (1) Diet (N = 118), (2) Exercise (N = 117), (3) Diet and Exercise (N = 117)<br><br>(1) Diet<br><br>● **Content:** Modification of the Diabetes Prevention Program and Look AHEAD lifestyle behavior change programs, including topics such as goal setting, problem solving, self-monitoring and coping strategies.<br>● **Intensity and delivery:** Minimum of 32 sessions over a total period of 12 months delivered by registered dietitians with training in behaviour modification. Sessions were delivered face to face and over phone/ email and included individual as well as group-based sessions.<br><br>(2) Exercise<br><br>● **Content:** Facility based exercise training program (starting with 15 min at 60–70% maximal heart rate, progressing to the target 70–85% maximal heart rate for 45 min by the 7th week and maintaining this for the remainder of the study)	Outcome(s) and Measure(s):<br><br>● Restraint (TFEQ-R18)<br>● Uncontrolled Eating (TFEQ-R18)<br>● Emotional Eating (TFEQ-R18)<br><br>Assessment time points:<br><br>● 12 months

			• **Intensity and delivery:** 3 weekly face to face sessions delivered by a certified exercise psychologist for 12 months + home based exercise<br><br>(3) Diet + Exercise<br><br>• **Content:** Both the diet and exercise programs<br>• **Intensity and delivery:** Both the diet and exercise programs<br><br><u>Comparator (N = 87):</u><br><br>• **Type:** Waitlist<br>• **Content:** n/a<br>• **Intensity and delivery:** n/a	
McRobbie et al. (2016) [19]	<u>Study design:</u><br>Two-arm RCT<br><br><u>Country:</u><br>United Kingdom<br><br><u>Setting:</u><br>Clinical<br><br><u>Eligibility:</u><br>Adults (≥18y) with a BMI ≥ 30kg/m^2 or a BMI ≥ 28kg/m^2 plus comorbidities	<u>Sex:</u> 72% female<br><br><u>Age in years[1]:</u><br>Comparison: 45.1 (14.2)<br>Intervention: 46.6 (15)<br><br><u>BMI (kg/m^2)[1]:</u><br>Comparison: 35.7 (4.3)<br>Intervention: 35 (4.2)<br><br><u>Ethnicity:</u><br>Black or other ethnic minority communities: 48%<br>White British: 40%	<u>Intervention</u>: Multimodal health behaviour modification intervention (N = 221)<br><br>• **Content:** behaviour change techniques, e.g. relating to dietary advice and education, goal setting, self-monitoring, motivation (incorporating cognitive behavioural elements) and non-judgemental support. The programme was developed with feedback from underprivileged groups.<br>• **Intensity and delivery:** 8 weekly group face to face sessions with 10-20 participants of 1 hour duration followed	<u>Outcome(s) and Measure(s):</u><br><br>• Restraint (TFEQ)<br>• Uncontrolled Eating (TFEQ)<br>• Emotional Eating (TFEQ)<br><br><u>Assessment time points:</u><br><br>• 2 months<br>• 6 months<br>• 12 months

		White other: 12%<br><br><u>Education:</u><br>Left school before age 16: 38%<br>No higher education: 51%<br><br><u>Income:</u><br>52% no paid employment	by 10 monthly group maintenance sessions open to all participants<br><br><u>Comparator (N = 109):</u><br><br>● **Type:** Usual care<br>● **Content:** standard advice on diet and physical activity based on NHS 'Change4Life' materials and motivational support<br>● **Intensity and delivery:** four individual face to face sessions delivered over 8 weeks by a practice nurse	
Mueller et al. (2022) [20]	<u>Study design:</u><br>Two-arm RCT<br><br><u>Country:</u><br>United Kingdom<br><br><u>Setting:</u><br>Remote<br><br><u>Eligibility:</u><br>Adults (≥18y) with a BMI ≥ 25kg/m^2	<u>Sex:</u> 78.1% female<br><br><u>Age in years[1]:</u> 50.3 (13.8)<br><br><u>BMI (kg/m^2)[1]:</u> 34.8 (7.7)<br><br><u>Ethnicity:</u><br>White: 93.8%<br>Non-White: 5.2%<br>None of these: 0.3%<br>Prefer not to say: 0.8%<br><br><u>Education:</u><br>Below post-secondary (up to and including A-levels): 25.8%<br>Post-secondary: 72.7%<br>Other: 1.3%<br>Prefer not to say: 0.3%	<u>Intervention</u>: SWiM-C (N = 192)<br><br>● **Content:** Acceptance and Commitment Therapy (ACT), including psychoeducation, reflective exercises, and behavioural experiments.<br>● **Intensity and delivery:** Online self-help programme containing 12 weekly online modules + weekly automated email reminders + two semi-structured phone calls of approx. 20 minutes with trained non-specialist coaches + tailored emails after session 4 and 10<br><br><u>Comparator (N = 196):</u><br><br>● **Type:** Minimal, waitlist	<u>Outcome(s) and Measure(s):</u><br><br>● Restraint (TFEQ-R21)<br>● Uncontrolled Eating (TFEQ-R21)<br>● Emotional Eating (TFEQ-R21)<br><br><u>Assessment time points:</u><br><br>● 4 months

		Income: n/a	• **Content:** standard advice in the form of a leaflet from the European Association on the Study of Obesity on diet, physical activity, and mood during the COVID 19 pandemic, tailored to people living with obesity<br>• **Intensity and delivery:** leaflet posted to participants home	
Munsch et al. (2003) [21]	Study design:<br>Two-arm RCT (contained one non-randomised group, Clinic BASEL, which was excluded from meta-analyses due to non-randomised nature)<br><br>Country:<br>Switzerland<br><br>Setting:<br>Clinical<br><br>Eligibility:<br>Adults (≥18y) with a BMI ≥ 30kg/m^2	Sex: 75.4%<br><br>Age in years[1]: 45.2 (23.9)<br><br>BMI (kg/m^2)[1]:<br>Comparison (GP Control) female: 34 (3)<br>Comparison (GP Control) male: 33.4 (2.5)<br><br>Intervention (GP Basel) female: 35.7 (5.6)<br>Intervention (GP Basel) male: 36.8 (5.2)<br><br>Ethnicity: n/a<br><br>Education: n/a<br><br>Income: n/a	Intervention: GP Basel (N = 53)<br><br>• **Content:** Cognitive Behavioural Therapy (CBT)<br>• **Intensity and delivery:** X a total of 16 face to face group sessions of 90 min each delivered by trained practitioners and tutors (trained by a psychologist and a dietician)<br><br>Comparator (N = 17):<br><br>• **Type:** Usual care<br>• **Content:** non-specific comments about general measures to lose weight. No specific technique, tools or written material was used.<br>• **Intensity and delivery:** n/a	Outcome(s) and Measure(s):<br><br>• Restraint (German version of the TFEQ)<br>• Disinhibition "distractibility" (German version of the TFEQ)<br>• Hunger (German version of the TFEQ) (TFEQ)<br><br>Assessment time points:<br><br>• Posttreatment<br>• 12 months from posttreatment
Nourizadeh et al. (2020) [22]	Study design:<br>Two-arm RCT	Sex: 100% female<br><br>Age in years[1]:	Intervention: Motivational interviewing (N = 35)<br><br>• **Content:** Motivational Interviewing (MI)	Outcome(s) and Measure(s):<br><br>• Restraint (TFEQ-R18)

	Country:<br>Iran<br><br>Setting:<br>Clinical<br><br>Eligibility:<br>Women before conception (≥18y ≤35y) with a BMI between 25 and 35kg/m^2	Comparison: 28.7 (4.2)<br>Intervention: 28.2 (4.8)<br><br>BMI (kg/m^2)[1]:<br>Comparison: 30.6 (2.5)<br>Intervention: 30 (2.6)<br><br>Ethnicity: n/a<br><br>Education:<br>Middle school comparison: 42.9%<br>Middle school Intervention: 45.7%<br><br>High school and diploma comparison: 51.4%<br>High school and diploma intervention: 40%<br><br>College education comparison: 5.7%<br>College education intervention: 14.3%<br><br>Income: n/a	• **Intensity and delivery:** 6 weekly face to face group sessions (8-12 women) of 60-90 minutes duration delivered by the second author who is a certified counsellor in MI<br><br>Comparator (N = 35):<br><br>• **Type:** Usual care<br>• **Content:** routine preconception care including immunization and prescription of folic acid along with physical examination and laboratory tests for identifying high-risk cases, presenting instructions to delay pregnancy, and taking diagnostic and therapeutic measures.<br>• **Intensity and delivery:** n/a	• Uncontrolled Eating (TFEQ-R18)<br>• Emotional Eating (TFEQ-R18)<br><br>Assessment time points:<br><br>• 14 weeks
Nurkkala et al. (2015) [23]	Study design:<br>Two-arm RCT<br><br>Country:<br>Finland	Sex: 78% female<br><br>Age in years[1]: 45 (11)	Intervention: Counselling (N = 90)<br><br>• **Content:** Counselling including topics such as healthy diet, risk situations in weight management and physical	Outcome(s) and Measure(s):<br><br>• Restraint (TFEQ-R18)<br>• Uncontrolled Eating (TFEQ-R18)

	Setting:<br>n/a<br><br>Eligibility:<br>Adults (≥18y ≤65y) with a BMI ≥ 30kg/m^2	BMI median (Interquartile range): 35.6 (33.2; 40.1)<br><br>Ethnicity: n/a<br><br>Education years[1]: 13.7 (3.3)<br><br>Income: n/a	activity. Eating behaviour was taken into account in the counselling by questionnaires, exercises and diaries about supporting permanent change in eating behaviour to achieve successful weight loss management.<br>● **Intensity and delivery:** 9-month weight loss period followed by 27- month weight maintenance period with individual face to face counselling sessions delivered by a nutritionist or a qualified nurse.<br>○ 1st year: 3x sessions with nutritionist plus 11x sessions with nurse<br>○ 2nd year: 4x sessions with nurse<br>○ 3rd year: 2x sessions with nurse<br><br>Comparator (N = 30):<br><br>● **Type:** Minimal<br>● **Content:** Brief advice<br>● **Intensity and delivery:** One meeting with a qualified nurse + booklet concerning the principles of weight management.	● Emotional Eating (TFEQ-R18)<br><br>Assessment time points:<br><br>● 9 months<br>● 24 months<br>● 36 months
Palmeira et al. (2017) [24]	Study design:<br>Two-arm RCT<br><br>Country:<br>Portugal<br><br>Setting:<br>Clinical	Sex: 100% female<br><br>Age in years[1]:<br>Comparison: 42.73 (8.36)<br>Intervention: 41.97 (8.79)<br><br>BMI (kg/m^2)[1]:<br>Comparison: 33.65 (4.83)	Intervention: Kg-free (N = 36)<br><br>● **Content:** Usual care + Acceptance and Commitment Therapy (ACT)<br>● **Intensity and delivery:** 10 weekly + 2 fortnightly booster face to face manualised group sessions (10-12	Outcome(s) and Measure(s):<br><br>● Uncontrolled Eating (TFEQ)<br>● Emotional Eating (TFEQ)

	Eligibility:<br>Women with a BMI ≥ 25kg/m^2 enrolled in nutritional treatment for weight loss in primary care units and Hospitals from Coimbra, Portugal	Intervention: 34.82 (5.26)<br><br>Ethnicity: n/a<br><br>Education years[1]:<br>Comparison: 15.35 (3.45)<br>Intervention: 14.94 (3.03)<br><br>Income:<br>Comparison low socioeconomic status: 10.8%<br>Intervention low socioeconomic status: 22.2%<br><br>Comparison medium socioeconomic status: 83.8%<br>Intervention medium socioeconomic status: 61.1%<br><br>Comparison high socioeconomic status:5.4%<br>Intervention high socioeconomic status: 16.7%	participants) of 2 hours and 30 mins in duration delivered by a clinical psychologist with previous training in contextual-behavioural therapies and one clinical psychology master student + a printed manual including targeted constructs, examples, and exercise sheets + audio files for mindfulness ad compassion exercises between sessions<br><br>Comparator (N = 37):<br><br>● **Type:** Waitlist, usual care<br>● **Content:** Individual medical and nutritional appointments. Medical appointments include physical examination, addressing comorbidities, discussing difficulties regarding weight loss plans. Nutritional appointments include weighing, tailored dietary recommendations (according to one's needs and food preferences) and physical activity prescriptions. No psychological treatment was included.<br>● **Intensity and delivery:** n/a	Assessment time points:<br><br>● 10 weeks
Paul et al. (2021) [25]	Study design:<br>Two-arm RCT<br><br>Country:<br>Netherlands	Sex: 74% female<br><br>Age in years[1]: 41.7 (9.7)<br><br>BMI (kg/m^2)[1]:<br>Comparison: 43.4 (5.4)	Intervention: Pre-operative CBT (N = 63)<br><br>● **Content:** Usual care + Cognitive Behavioural Therapy (CBT), focused on nutritional and activity management	Outcome(s) and Measure(s):<br><br>● Restraint (DEBQ)<br>● External Eating (DEBQ)<br>● Emotional Eating (DEBQ)

	Setting:<br>Clinical<br><br>Eligibility:<br>Adults (≥21y ≤65y) awaiting bariatric surgery	Intervention: 42.7 (5)<br><br>Ethnicity: n/a<br><br>Education level:<br>High: 27%<br>Middle: 65%<br>Low: 9%<br><br>Income: n/a	(sessions 1–4), cognitive restructuring and developing alternative behaviour (sessions 5–8), and relapse prevention strategies and preparation for the postoperative period (sessions 9– 10).<br>● **Intensity and delivery:** 10 weekly individual face to face sessions of 45 minute duration<br><br>Comparator (N = 65):<br><br>● **Type:** Usual care<br>● **Content:** Pre-operative care<br>● **Intensity and delivery:** One mandatory group meeting providing information on the surgery + One booklet with information on the surgery	Assessment time points:<br><br>● 10 weeks
Porca et al. (2021) [26]	Study design:<br>Two-arm RCT<br><br>Country:<br>Spain<br><br>Setting:<br>Clinical<br><br>Eligibility:<br>Adults (≥18y) with a BMI ≥ 30kg/m^2 referred to the Division of Endocrinology and	Sex: 81.5% female<br><br>Age in years[1]: 48.8 (12.9)<br><br>BMI (kg/m^2)[1]: 39.9 (7)<br><br>Ethnicity: n/a<br><br>Education: n/a<br><br>Income: n/a	Intervention: Habit change (N = 221)<br><br>● **Content:** Usual care + structured program of habit change and exercise based on behavioural therapy. Topics included nutrition education, physical activity, managing emotional eating<br>● **Intensity and delivery:** 6 weekly face to face group sessions (15 participants) of 1h duration delivered by a nutritionist and a nurse plus 2 follow-up visits (at six months and at 12 months) + email for social support system + website for information and recipes + homework	Outcome(s) and Measure(s):<br><br>● Eating for psychological wellbeing (Habits Questionnaire)<br><br>Assessment time points:<br><br>● 12 months

	Nutrition of the reference Hospital		Comparator (N = 211):<br><br>● **Type:** Usual care<br>● **Content:** Patients received recommendations for lifestyle change, diet prescription and options for body weight controls performed by the nursing team<br>● **Intensity and delivery:** 2 individual face to face clinical revision visits at 6 and 12 months (only the later one was mandatory) delivered by an endocrinology specialist and a nurse	
Potts et al. (2022) [27]	Study design:<br>Three-arm RCT (combined for meta-analyses)<br><br>Country:<br>United States<br><br>Setting:<br>Remote<br><br>Eligibility:<br>Adults (≥18y ≤64y) with problematic weight self-stigma (score of ≥36 on the weight self-stigma	Sex: 81.8% female<br><br>Age in years[1]: 38.65 (12.4)<br><br>BMI (kg/m^2)[1]: 37.01 (6.51)<br><br>Ethnicity:<br>Non-Hispanic/Latino White: 89.09%<br>Hispanic/Latino White: 5.45%<br>Hispanic/Latino of Other Race: 3.64%<br>Black: 1.82%<br><br>Education: n/a<br><br>Income: median income range $40,000 to $59,999	Intervention: (1) Guided self-help with phone coaching (GSH-P) (N = 17) or (2) guided self-help with email prompts (GSH-E) (N = 20)<br><br>● **Content:** Acceptance and Commitment Therapy (ACT) based on "the diet trap", a guided self-help book<br>● **Intensity and delivery:** Participants in both GSH-E and GSH-P conditions were sent a physical copy of The Diet Trap and asked to read one of seven book chapters each week for the first 7 weeks + asked to complete the journaling activities contained within the book, and to complete a weekly online Qualtrics quiz for each chapter they finished.	Outcome(s) and Measure(s):<br><br>● Emotional Eating (DEBQ)<br><br>Assessment time points:<br><br>● 8 weeks

	questionnaire) and a BMI ≥ 27.5kg/m^2		○ GSH-E: weekly templated email focusing on adherence to the book. If a participant did not complete the weekly quiz, up to two reminders were sent.<br>○ GSH-P: same as GSH-E plus 8 weekly phone calls of 5-30 minutes duration focussing mainly on adherence to the book<br><br>Comparator (N = 18):<br><br>● **Type:** Waitlist<br>● **Content:** n/a<br>● **Intensity and delivery:** n/a	
Roehling et al. (2019) [28]	Study design:<br>Two-arm RCT<br><br>Country:<br>Germany<br><br>Setting:<br>Workplace<br><br>Eligibility:<br>Employees of the Düsseldorf Catholic Hospital Group (≥18y) with a BMI ≥ 25kg/m^2	Sex:<br>Comparison: 87% female<br>Intervention: 80% female<br><br>Age in years[1]:<br>Comparison: 49 (7)<br>Intervention: 44 (9)<br><br>BMI (kg/m^2)[1]:<br>Comparison: 32.8 (6.1)<br>Intervention: 35.1 (6.9)<br><br>Ethnicity: n/a<br><br>Education: n/a	Intervention: SAMMAS (N = 15)<br><br>● **Content:** Education and telemedical coaching, including topics on nutrition education, optional meal replacement, continuous glucose monitoring, control of hunger and suggestibility, physical activity, acid-base balance<br>● **Intensity and delivery:** 12 weekly contacts, including 7 group based face to face theoretical sessions (90 mins duration), 2 practical modules (90 mins duration), 4 care calls (20-30 mins duration) delivered by academic experts, such as nutritionists, exercise scientists,	Outcome(s) and Measure(s):<br><br>● Restraint (German version of the TFEQ)<br>● Disinhibition "suggestibility" (German version of the TFEQ)<br>● Hunger (German version of the TFEQ)<br><br>Assessment time points:<br><br>● 12 weeks

		Income: n/a	biologists and physicians in cooperation with psychologists + personalised online portal + continuous glucose monitoring device + optional structured meal replacement<br><br>Comparator (N = 15):<br><br>● **Type:** Waitlist<br>● **Content:** All participants were equipped with telemetric devices (scales and pedometers).<br>● **Intensity and delivery:** n/a	
Salvo et al. (2022) [29]	Study design: Three-arm RCT (only two meet eligibility criteria and were included in meta-analyses)<br><br>Country: Brazil<br><br>Setting: Clinical<br><br>Eligibility: Women (≥18y ≤60y) with a BMI between 25 and 40kg/m^2	Sex: 100% female<br><br>Age in years[1]: 40.4 (10.7)<br><br>BMI (kg/m^2)[1]: 32.7 (3.8)<br><br>Ethnicity: n/a<br><br>Education:<br>Elementary school: 24%<br>High school: 62%<br>Higher education: 14%<br><br>Income: n/a	Intervention: MB-EAT (N = 95)<br><br>● **Content:** Usual care + Mindfulness programme, raising awareness of hunger, fullness, taste awareness, and triggers for eating and introducing nutrition knowledge. The main mindfulness practices used are mindfulness of breathing, mindfulness of the body (body scan), mindful walking and mindful movements.<br>● **Intensity and delivery:** 10 weekly face to face group sessions + homework + handouts and audio files + email reminders<br><br>Comparator (N = 96):	Outcome(s) and Measure(s):<br><br>● Mindful Eating (MES)<br><br>Assessment time points:<br><br>● 10 weeks<br>● 3 months

			● **Type:** Usual care<br>● **Content:** Usual care depends on participants BMI and presence of co-morbidities<br>○ BMI 25-30 and no co-morbidities: Primary care teams organised care plans<br>○ BMI 25-30 plus co-morbidities: Dietary prescriptions offered by nutritionist if deemed necessary<br>○ BMI 30 – 40 with or without co-morbidities: PC teams must assess the need for the provision of behavioural and pharmacotherapy and must organise it when appropriate.<br>○ BMI > 40 are assisted by specialised services (ambulatory or hospital setting)<br>● **Intensity and delivery:** n/a	
Steinberg et al. (2014) [30]	Study design:<br>Two-arm RCT<br><br>Country:<br>United States<br><br>Setting:<br>Remote<br><br>Eligibility:<br>Adults (≥18y ≤60y) with a BMI	Sex: 74%<br><br>Age in years[1]: 44 (11)<br><br>BMI (kg/m^2)[1]: 32.15 (3.8)<br><br>Ethnicity: 74% white<br><br>Education: 87% college educated<br><br>Income: n/a	Intervention (N = 47):<br><br>● **Content:** Daily self-weighing intervention incorporating educational and behavioural lessons on topics such as portion size, restaurant eating, structured exercise, problem solving, stimulus control, and relapse prevention<br>● **Intensity and delivery:** 22 weekly lessons over email and weekly tailored feedback	Outcome(s) and Measure(s):<br><br>● Restraint (TFEQ-51)<br>● Disinhibition (TFEQ-51)<br>● Hunger (TFEQ-51)<br><br>Assessment time points:<br><br>● 3 months<br>● 6 months

	between 25 and 40kg/m^2		on self-weighing over email + "smart" scales<br><br><u>Comparator (N = 44):</u><br>● **Type:** Waitlist<br>● **Content:** n/a<br>● **Intensity and delivery:** n/a	
Whitelock et al. (2019) [31]	<u>Study design:</u><br>Two-arm RCT<br><br><u>Country:</u><br>United Kingdom<br><br><u>Setting:</u><br>Remote<br><br><u>Eligibility:</u><br>Adults (≥18y ≤65y) with a BMI ≥25kg/m^2	<u>Sex:</u><br>Comparison: 70.4%<br>Intervention: 77.4%<br><br><u>Age in years[1]:</u><br>Comparison: 44.5 (10.7)<br>Intervention: 42.8 (10.5)<br><br><u>BMI (kg/m^2)[1]:</u><br>Comparison: 35.2 (6.2)<br>Intervention: 35.9 (6.8)<br><br><u>Ethnicity:</u><br>White comparison: 94.4%<br>White intervention: 92.5%<br><br>Mixed/ multiple: 1.9%<br><br>Asian comparison: 3.7%<br>Asian intervention: 5.7%<br><br><u>Education:</u><br>Entry level or equivalent comparison: 7.4%	<u>Intervention</u>: (N = 53)<br>● **Content:** Dietary advice + Attentive eating<br>● **Intensity and delivery:** Dietary advice + attentive eating application (included photographing and reflecting on meals. App also sent reminders and audio was instructed to be listened to a few times a week) + attentive eating leaflet<br><br><u>Comparator (N = 54):</u><br>● **Type:** Minimal<br>● **Content:** dietary advice adapted from British Heart Foundation materials on healthy eating and weight loss (e.g. components of a balanced diet, reducing calories and lower calorie swaps, consuming fruits and vegetables, avoiding foods high in fat and sugar, drinks, shopping and eating out) and brief	<u>Outcome(s) and Measure(s):</u><br>● Restraint (TFEQ-R21)<br>● Uncontrolled Eating (TFEQ-R21)<br>● Emotional Eating (TFEQ-R21)<br><br><u>Assessment time points:</u><br>● 8 weeks

		Entry level or equivalent intervention: 0%<br><br>GCSE's or equivalent comparison: 14.8%<br>GCSE's or equivalent intervention: 17%<br><br>A/AS level or equivalent comparison: 14.8%<br>A/AS level or equivalent intervention: 22.6%<br><br>Undergraduate degree or equivalent comparison: 33.3%<br>Undergraduate degree or equivalent intervention: 37.7%<br><br>Higher degree or equivalent comparison: 27.8%<br>Higher degree or equivalent intervention: 17%<br><br><u>Income:</u> n/a	information about the importance of physical activity<br>● **Intensity and delivery:** booklet and 8 weekly text messages relating to the same dietary advice	

[1] mean (SD)

Abbreviations: RCT, Randomised controlled trial; BMI, Body mass index; N, Number of participants; **Abbreviations Questionnaires:** TFEQ, Three Factor Eating Questionnaire; PFS, Power of Food Scale; EDE-R, Eating Disorder Inventory Restraint Subscale; EOQ, Emotional Overeating Questionnaire; EES, Emotional Eating Scale; MES, Mindful Eating Scale; DEBQ, Dutch Eating Behaviour Questionnaire; IES, Intuitive Eating Scale; HTAS, Health and Taste Attitude Scales;

3.0 Risk of Bias

Study	D1	D2	D3	D5	Overall
Ahern et al. (2017)	+	+	+	+	+
Barnes et al. (2017)	-	+	+	!	-
Camolas et al. (2017)	!	+	+	+	!
Campos et al. (2022)	-	+	!	+	-
Cassin et al. (2016)	+	+	+	+	+
Czepczor-Bernat et al. (2020)	!	+	+	!	!
Daubenmeier et al. (2011)	+	+	+	!	!
Hilbert et al. (2021)	+	+	+	+	+
Jarvela-Reijonen et al. (2018)	+	+	+	+	+
Jebb et al. (2011)	+	+	+	+	+
Katzmarzyk et al. (2020)	+	+	+	+	+
Keranen et al. (2009)	+	+	-	!	-
Levin et al. (2021)	+	+	+	+	+
Mason et al. (2019)	-	+	+	+	-
McRobbie et al. (2016)	+	+	+	+	+
Mueller et al. (2022)	+	+	+	+	+
Munsch et al. (2003)	!	+	!	!	!
Nourizadeh et al. (2020)	+	+	+	+	+
Nurkkala et al. (2015)	+	+	+	+	+
Palmeira et al. (2019)	+	+	+	+	+
Paul et al. (2021)	+	+	+	+	+
Porca et al. (2021)	+	+	+	+	+
Potts et al. (2022)	+	+	+	!	!
Roehling et al. (2019)	+	+	+	+	+
Salvo et al. (2022)	+	+	+	+	+
Steinberg et al. (2014)	+	+	+	+	+
Whitelock et al. (2018)	+	+	+	+	+

+ Low risk
! Some concerns
- High risk

D1 Randomisation process
D2 Deviations from the intended interventions
D3 Missing outcome data
D5 Selection of the reported result

Figure S1 Risk of Bias of included trials

4.0 Intervention effects on EBTs at Follow-up

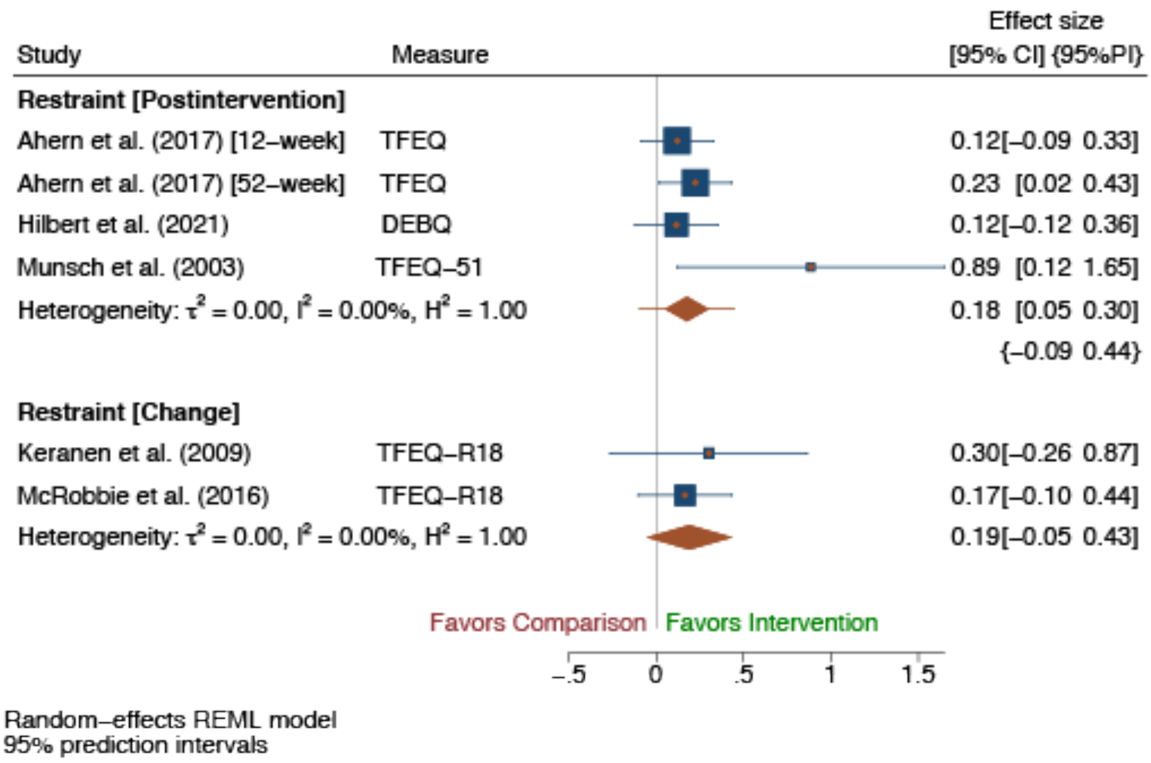

Figure S2 Outcomes where the desired intervention effect is an increase in the trait at follow-up.

Note: The size of blue squares representing each trial are proportional to the study weight. The width of the orange diamond representing overall effect size corresponds to the length of the CI, and its' orange whiskers correspond to the length of the PI. The height of the diamond is irrelevant.

Abbreviations: CI, Confidence Interval; PI, Prediction Interval; TFEQ, Three Factor Eating Questionnaire; TFEQ-51, Three Factor Eating Questionnaire original 51 item version; TFEQ-R18, Three Factor Eating Questionnaire revised 18 item version; DEBQ, Dutch Eating Behaviour Questionnaire

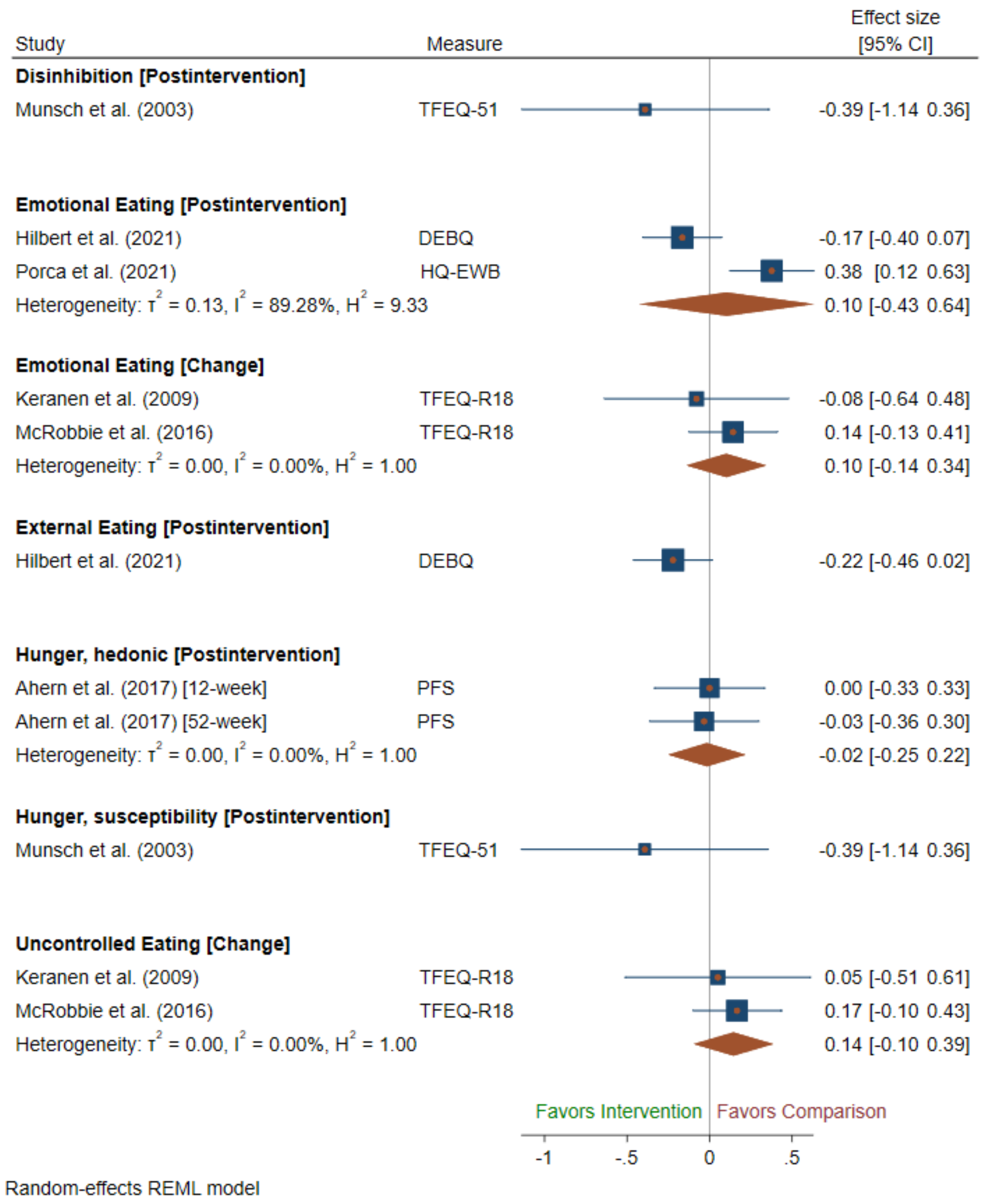

Figure S3 Outcomes where the desired intervention effect is a decrease in the trait at follow-up.

Note: The size of blue squares representing each trial are proportional to the study weight. The width of the orange diamond representing overall effect size corresponds to the length of the CI, and its' orange whiskers correspond to the length of the PI. The height of the diamond is irrelevant.

Abbreviations: CI, Confidence Interval; TFEQ-51, Three Factor Eating Questionnaire original 51 item version; TFEQ-R18, Three Factor Eating Questionnaire revised 18 item version; DEBQ, Dutch Eating Behaviour Questionnaire; PFS, Power of Food Scale; HQ-EWB, Eating habits questionnaire for patients with overweight and obesity eating for psychological wellbeing subscale

5.0 Sensitivity Analyses

5.1 Risk of Bias

5.1.1 Sensitivity analyses for risk of bias at end of intervention

Table S2 Prediction intervals for outcomes in which a decrease in the trait is the desired intervention effect at intervention end

Outcome		Effect Estimate [95% Prediction interval]
**Uncontrolled Eating**		
	Post- intervention outcomes	-0.25 [-0.484,-0.006]
	Change outcomes	-0.13 [-2.017, 1.757]
**Emotional Eating**		
	Post- intervention outcomes	-0.11 [-0.488, 0.278]
	Change outcomes	-0.17 [-1.034, 0.702]
**Hedonic Hunger**		
	Post- intervention outcomes	-0.09 [-1.470, 1.289]

Table S3 Prediction intervals for outcomes in which an increase in the trait is the desired intervention effect at intervention end

Outcome		Effect Estimate [95% Prediction interval]
**Restraint**		
	Post- intervention outcomes	0.38 [-0.421, 1.187]
	Change outcomes	0.48 [-0.854, 1.809]
**Intuitive Eating**		
	Post- intervention outcomes	0.23 [-0.200, 0.666]

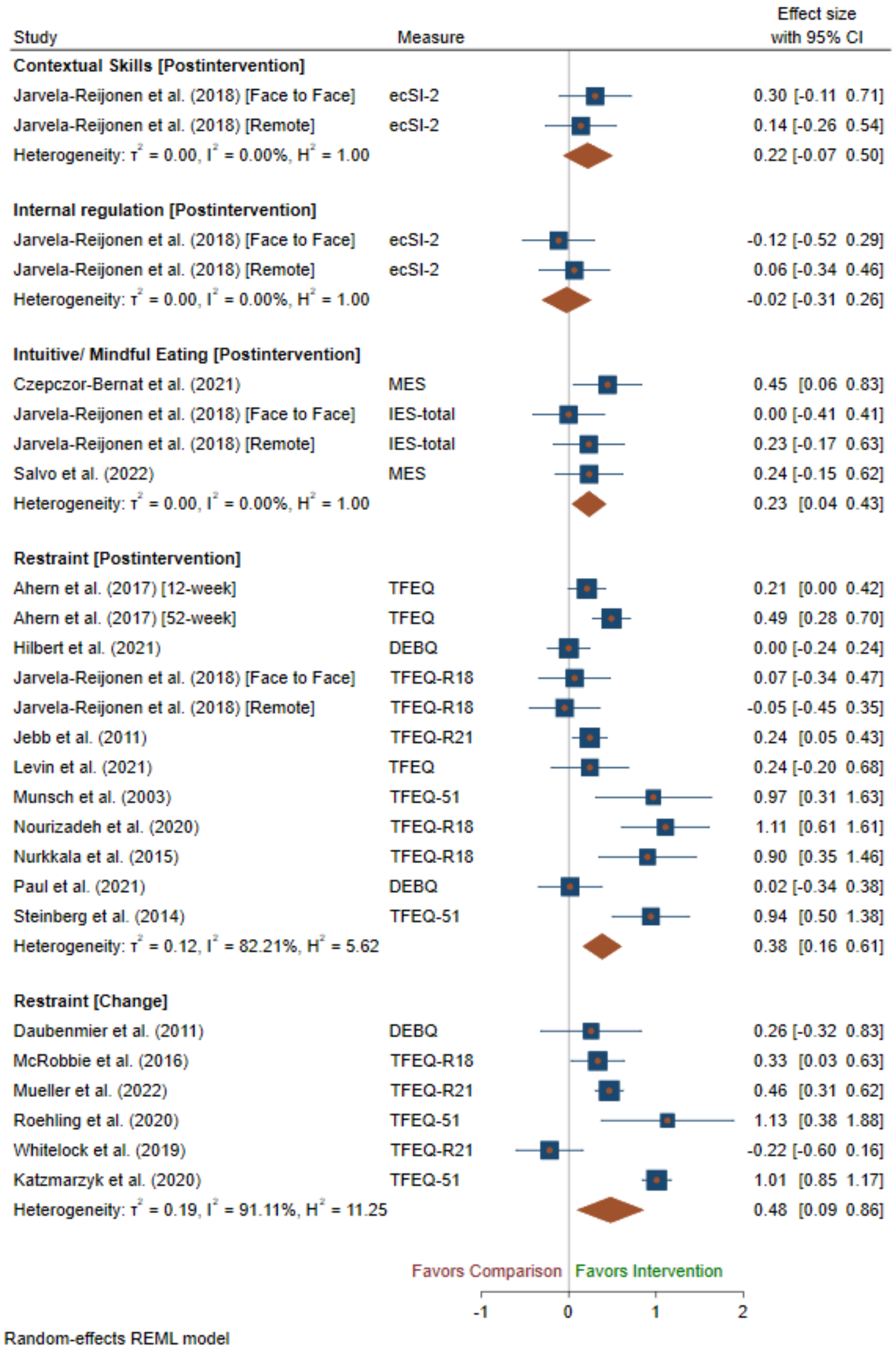

Figure S4 Outcomes where the desired intervention effect is an increase in the trait in trials with the risk of bias rated as "low" or "some concerns" at intervention end. *Abbreviations:* CI, Confidence Interval; TFEQ-51, Three Factor Eating Questionnaire original 51 item version; TFEQ-R18, Three Factor Eating Questionnaire revised 18 item version; TFEQ-R21, Three Factor Eating Questionnaire revised 21 item version; DEBQ, Dutch Eating Behaviour Questionnaire; MES, Mindful Eating Scale; IES, Intuitive Eating Scale; EDE-R, Eating Disorder Examination Questionnaire Restraint Subscale; ecSI-2, Satter Eating Competence Inventory

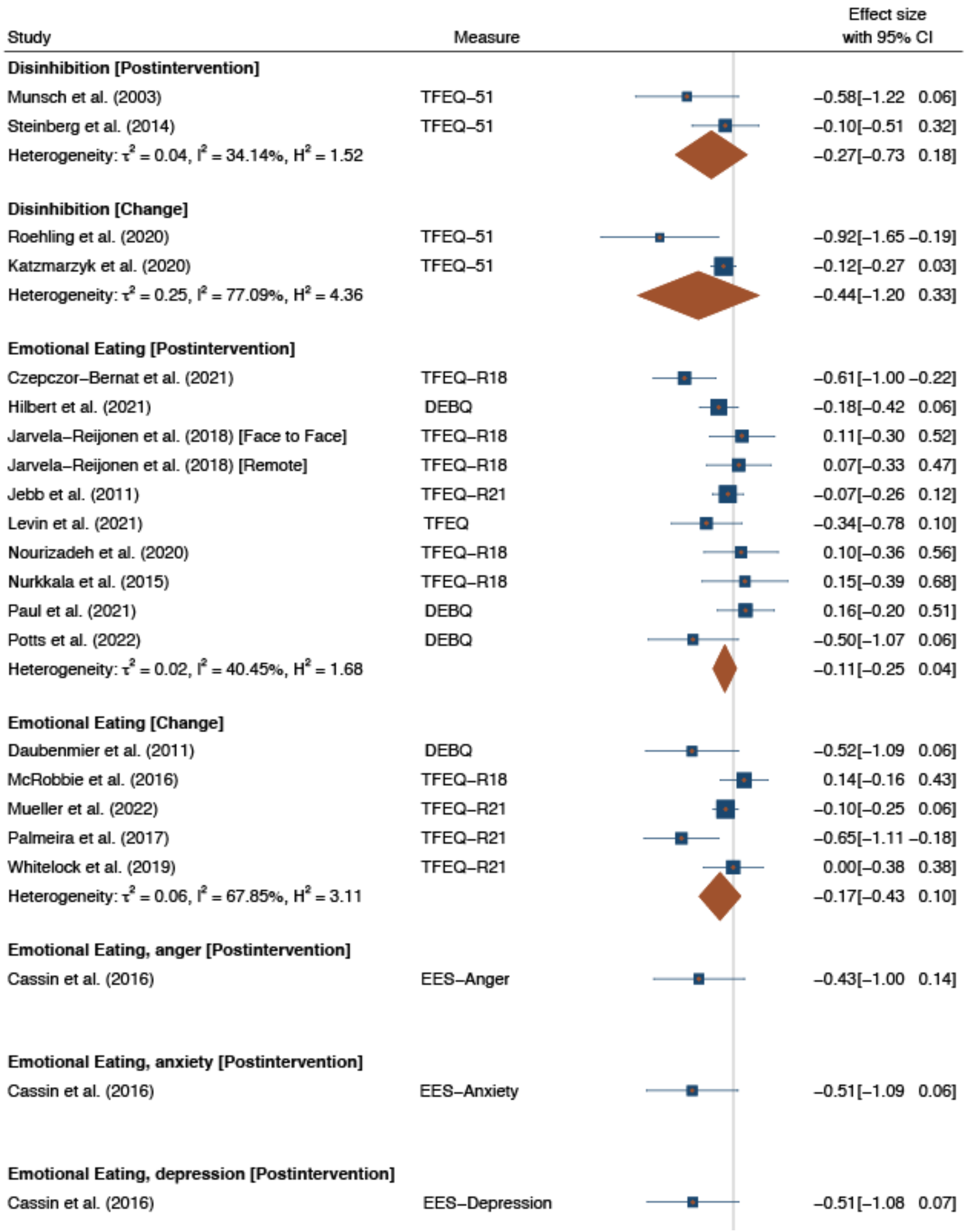

Figure S5 Outcomes where the desired intervention effect is a decrease in the trait in trials with the risk of bias rated as "low" or "some concerns" at intervention end. *Abbreviations:* CI, Confidence Interval; TFEQ-51, Three Factor Eating Questionnaire original 51 item version; TFEQ-R18, Three Factor Eating Questionnaire revised 18 item version; TFEQ-R21, Three Factor Eating Questionnaire revised 21 item version; DEBQ, Dutch Eating Behaviour Questionnaire; PFS, Power of Food Scale; EOQ, Emotional Overeating Questionnaire; EES, Emotional Eating Scale; HTAS, Health and Taste Attitude Scales

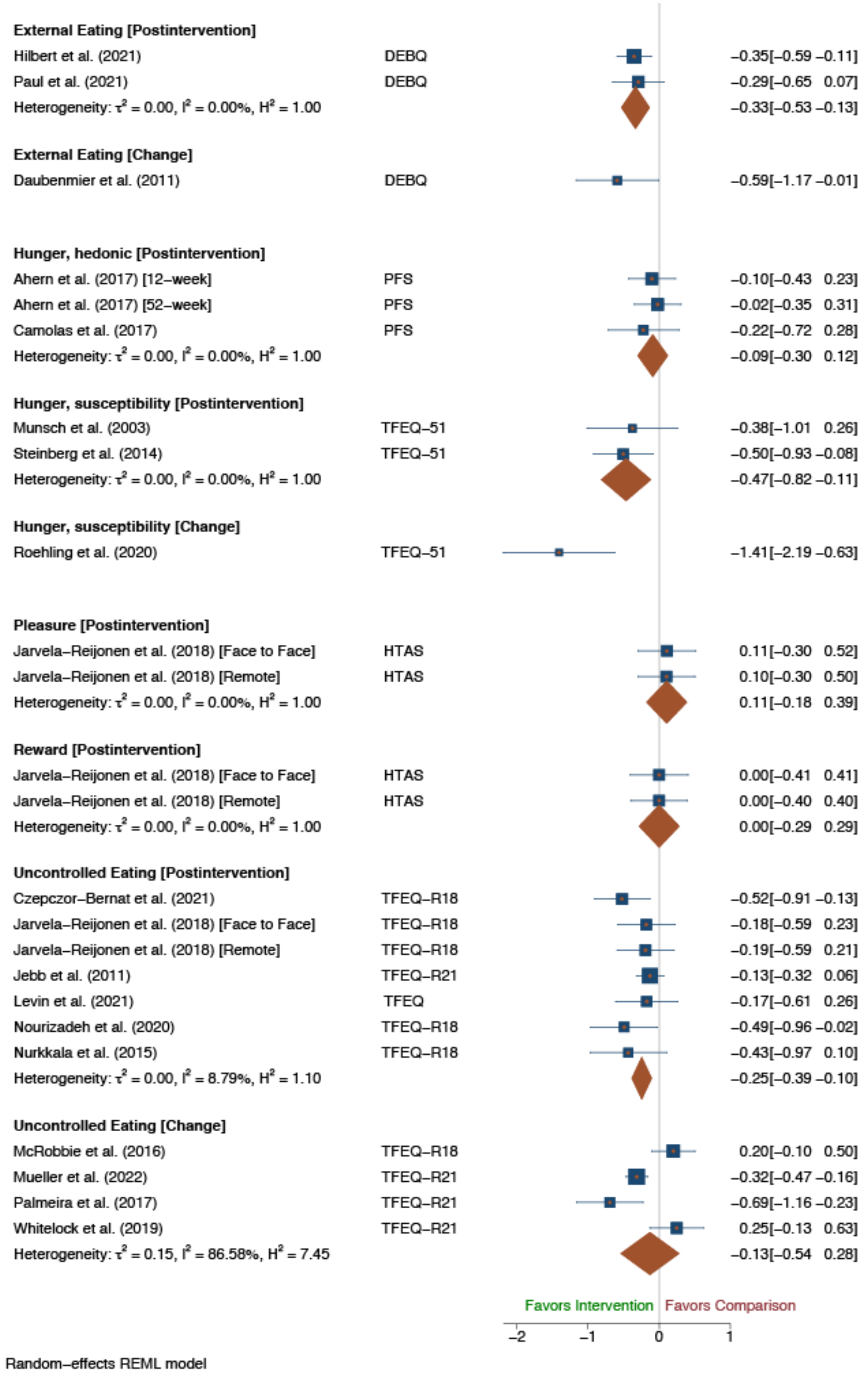

Figure S5 (continued)

5.1.2 Sensitivity analyses for risk of bias at follow-up

Table S4 Prediction intervals for outcomes in which an increase in the trait is the desired intervention effect at 12 months follow-up

Outcome	Effect Estimate [95% Prediction interval]
Restraint	
Post intervention outcomes	0.18 [-0.095, 0.448]

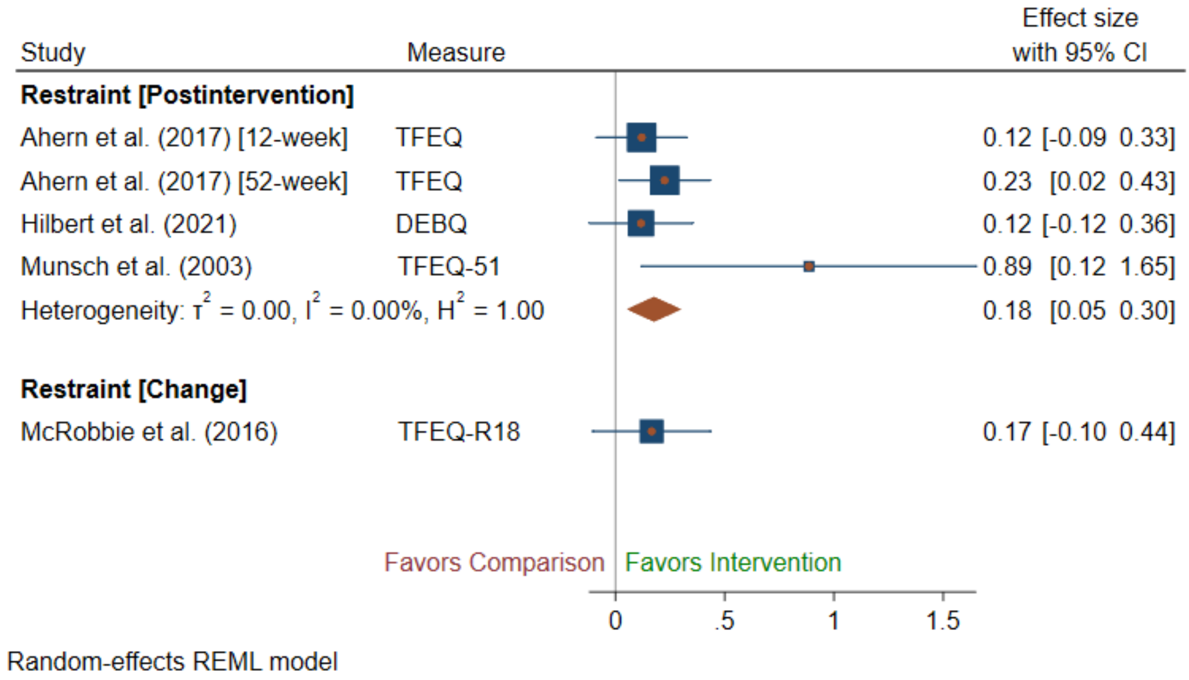

Figure S6 Outcomes where the desired intervention effect is an increase in the trait in trials with the risk of bias rated as "low" or "some concerns" at follow-up. *Abbreviations:* CI, Confidence Interval; TFEQ, Three Factor Eating Questionnaire; TFEQ-51, Three Factor Eating Questionnaire original 51 item version; TFEQ-R18, Three Factor Eating Questionnaire revised 18 item version; DEBQ, Dutch Eating Behaviour Questionnaire

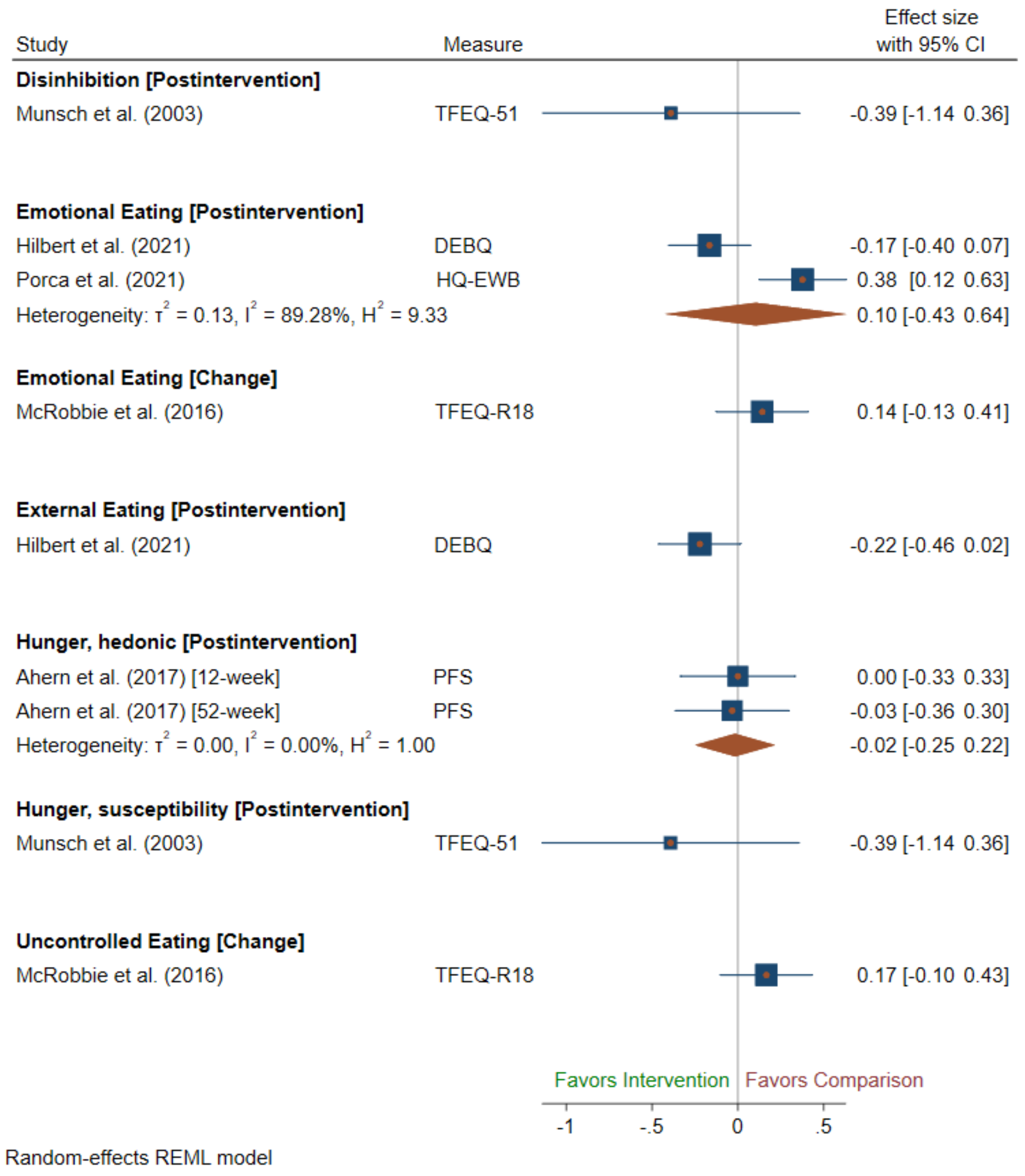

Figure S7 Outcomes where the desired intervention effect is a decrease in the trait in trials with the risk of bias rated as "low" or "some concerns" at follow-up. *Abbreviations:* CI, Confidence Interval; TFEQ-51, Three Factor Eating Questionnaire original 51 item version; TFEQ-R18, Three Factor Eating Questionnaire revised 18 item version; DEBQ, Dutch Eating Behaviour Questionnaire; PFS, Power of Food Scale; HQ-EWB, Eating habits questionnaire for patients with overweight and obesity eating for psychological wellbeing subscale

5.2 Study Design

5.2.1 Sensitivity analyses for study design at end of intervention

Table S5 Prediction intervals for outcomes in which a decrease in the trait is the desired intervention effect at intervention end

Outcome		Effect Estimate [95% Prediction interval]
Uncontrolled Eating		
	Post- intervention outcomes	-0.25 [-0.484,-0.006]
	Change outcomes	-0.20 [-1.130, 0.727]
Emotional Eating		
	Post- intervention outcomes	-0.11 [-0.497, 0.269]
	Change outcomes	-0.16 [-0.814, 0.488]
Hedonic Hunger		
	Post- intervention outcomes	-0.09 [-1.470, 1.289]

Table S6 Prediction intervals for outcomes in which an increase in the trait is the desired intervention effect at intervention end

Outcome		Effect Estimate [95% Prediction interval]
Restraint		
	Post- intervention outcomes	0.36 [-0.366, 1.078]
	Change outcomes	0.30 [-0.690, 1.293]
Intuitive Eating		
	Post- intervention outcomes	0.23 [-0.200, 0.666]

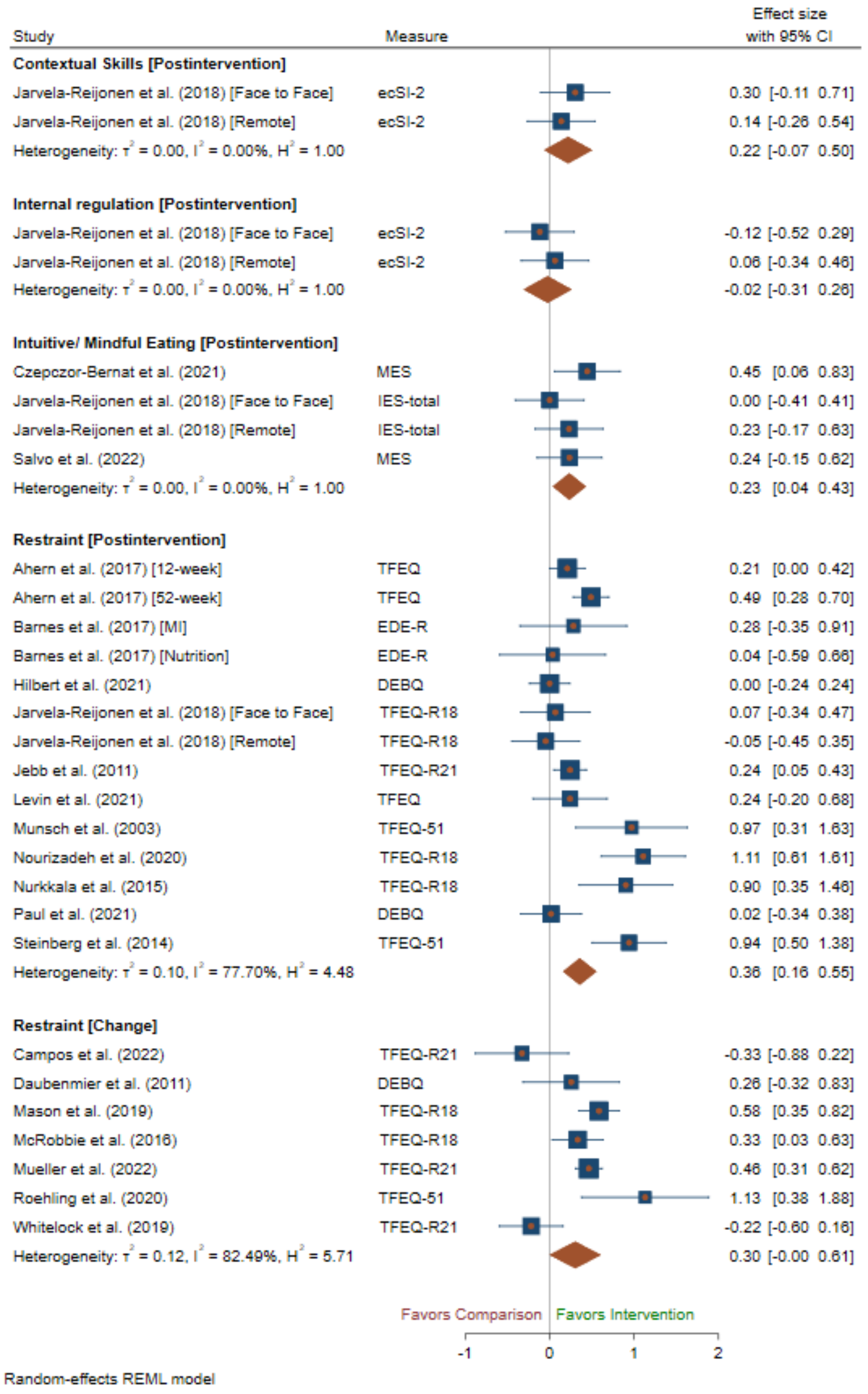

Figure S8 Outcomes where the desired intervention effect is an increase in the trait in randomised controlled trials at intervention end. *Abbreviations:* CI, Confidence Interval; TFEQ-51, Three Factor Eating Questionnaire original 51 item version; TFEQ-R18, Three Factor Eating Questionnaire revised 18 item version; TFEQ-R21, Three Factor Eating Questionnaire revised 21 item version; DEBQ, Dutch Eating Behaviour Questionnaire; MES, Mindful Eating Scale; IES, Intuitive Eating Scale; EDE-R, Eating Disorder Examination Questionnaire Restraint Subscale; ecSI-2, Satter Eating Competence Inventory

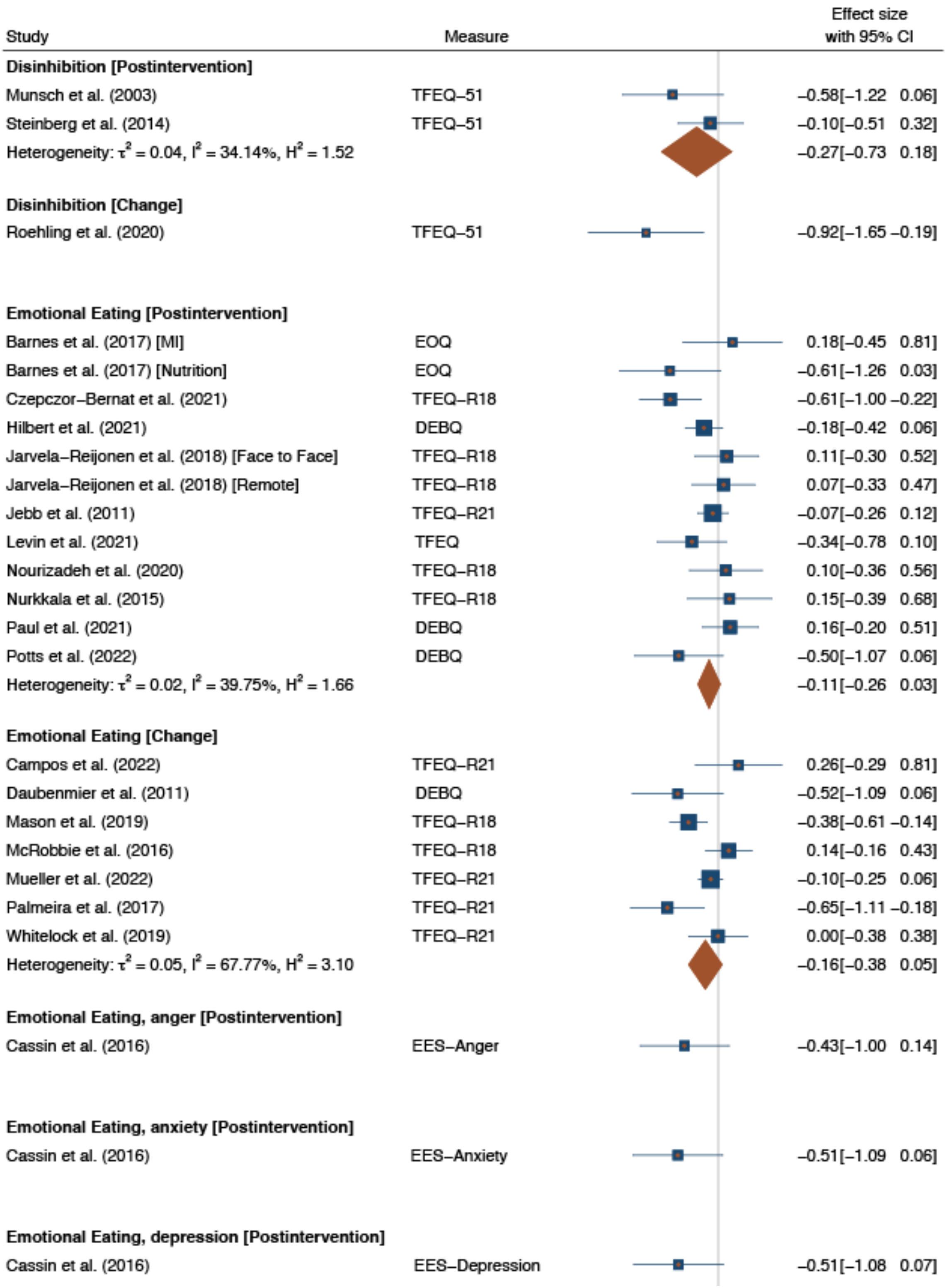

Figure S9 Outcomes where the desired intervention effect is a decrease in the trait in in randomised controlled trials at intervention end. *Abbreviations:* CI, Confidence Interval; TFEQ-51, Three Factor Eating Questionnaire original 51 item version; TFEQ-R18, Three Factor Eating Questionnaire revised 18 item version; TFEQ-R21, Three Factor Eating Questionnaire revised 21 item version; DEBQ, Dutch Eating Behaviour Questionnaire; PFS, Power of Food Scale; EOQ, Emotional Overeating Questionnaire; EES, Emotional Eating Scale; HTAS, Health and Taste Attitude Scales

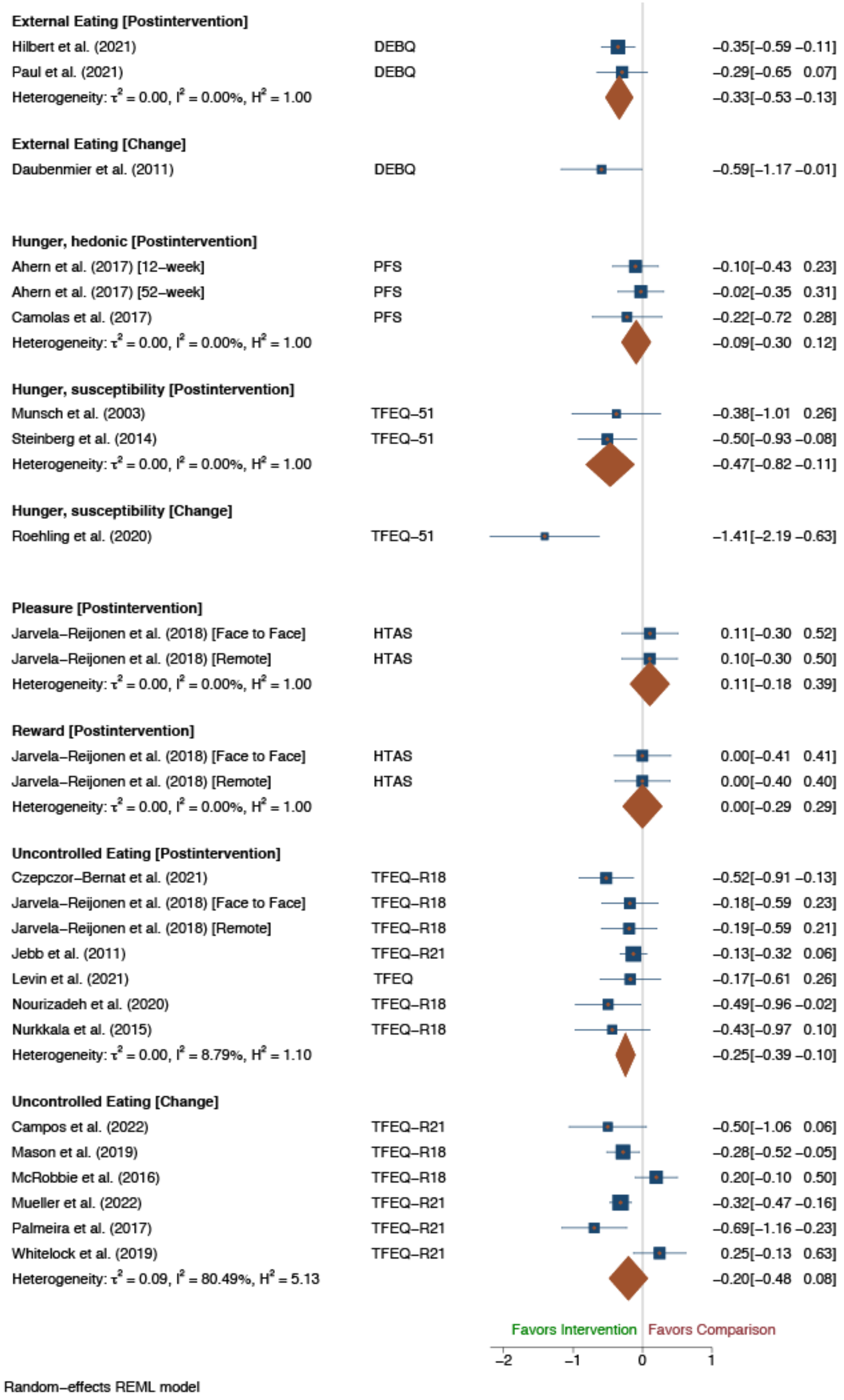

Figure S9 (continued)

5.2.2 Sensitivity analyses for study design at follow-up

Table S7 Prediction intervals for outcomes in which an increase in the trait is the desired intervention effect at 12 months follow-up

Outcome		Effect Estimate [95% Prediction interval]
Restraint		
	Post intervention outcomes	0.18 [-0.095, 0.448]

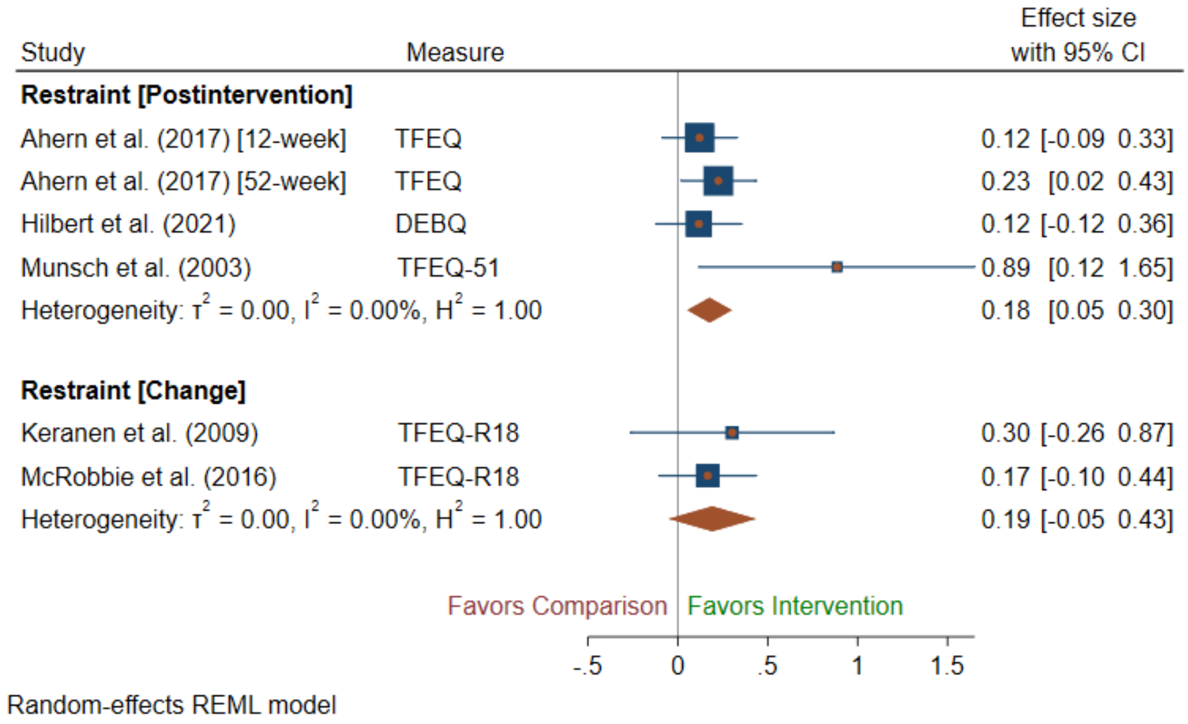

Figure S10 Outcomes where the desired intervention effect is an increase in the trait in in randomised controlled trials at follow-up. *Abbreviations:* CI, Confidence Interval; TFEQ, Three Factor Eating Questionnaire; TFEQ-51, Three Factor Eating Questionnaire original 51 item version; TFEQ-R18, Three Factor Eating Questionnaire revised 18 item version; DEBQ, Dutch Eating Behaviour Questionnaire

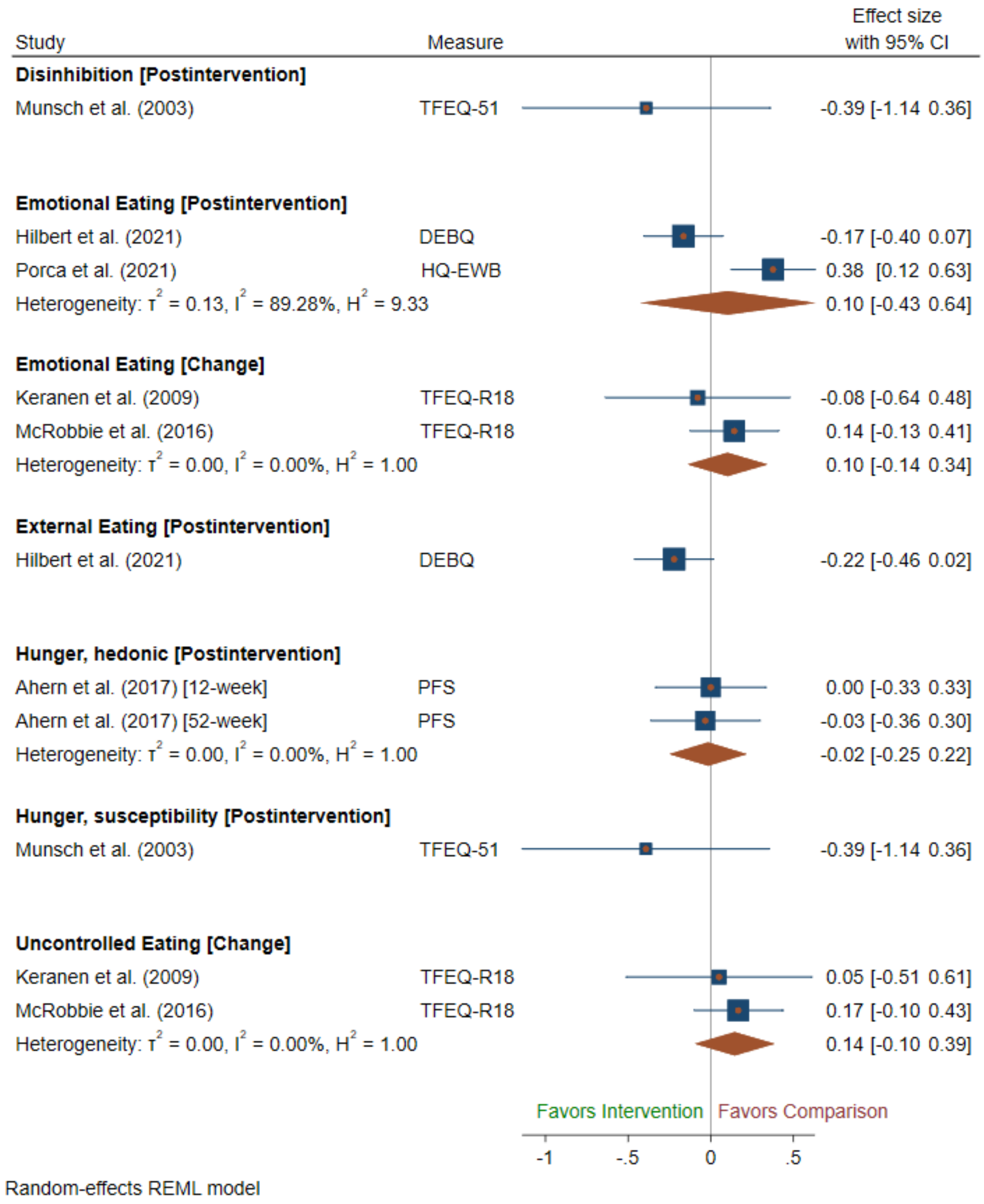

Figure S11 Outcomes where the desired intervention effect is a decrease in the trait in in randomised controlled trials at follow-up. *Abbreviations:* CI, Confidence Interval; TFEQ-51, Three Factor Eating Questionnaire original 51 item version; TFEQ-R18, Three Factor Eating Questionnaire revised 18 item version; DEBQ, Dutch Eating Behaviour Questionnaire; PFS, Power of Food Scale; HQ-EWB, Eating habits questionnaire for patients with overweight and obesity eating for psychological wellbeing subscale

6.0 Subgroup Analyses

6.1 Disinhibition

Due to insufficient contributing trial arms at both end of intervention (N = 3) and follow-up (N =1), no subgroup analyses were performed for disinhibition.

6.2 Emotional Eating

6.2.1 Impact on emotional eating by subgroups at end of intervention

Details on the impact of different subgroups on emotional eating at intervention end are presented in Figures S12, S14, S16, S18, S20 and S22 for post intervention outcomes and in Figures S13, S15, S17, S19, S21 and S23 for change outcomes. Due to insufficient contributing trial arms (N = 1), no subgroup analyses were performed for the outcomes of emotional eating anger, depression and anxiety.

Subgroup analyses by intervention type found no evidence of an effect on emotional eating in trials of standard behavioural interventions (Post intervention: SMD -0.09 [95%CI -0.26, 0.09]; N = 3; Change: SMD -0.13 [95%CI -0.63, 0.37]; N = 2), nor was there evidence of an effect in trials of third wave psychological interventions (Post intervention: SMD -0.24 [95%CI -0.54, 0.06]; N = 5; Change: SMD -0.18 [95%CI -0.46, 0.10]; N = 5). Only one study was classified as a second wave cognitive behavioural therapy intervention, finding no evidence of an effect on emotional eating (Post intervention: SMD 0.16 [95%CI -0.20, 0.51]; N = 1).

In subgroup analyses based on intervention duration, two trials with intervention durations longer than 12 weeks found no evidence of an effect on emotional eating for post intervention outcomes (Post intervention: SMD -0.04 [95%CI -0.22, 0.14]). Only one trial with an intervention duration longer than 12 weeks reported change outcomes and found evidence of an effect in favour of the intervention on emotional eating (Change: SMD -0.38 [95%CI -0.61, -0.14]). There was no evidence of an effect on emotional eating in interventions shorter than 12 weeks duration (Post intervention: SMD -0.15 [95%CI -0.33, 0.04]; N = 10; Change: SMD -0.17 [95%CI -0.43, 0.10]; N = 5).

Subgroup analyses by intervention delivery format found no evidence of an effect in interventions delivered on an individual basis (Post intervention: SMD -0.17 [95%CI -0.45, 0.11]; N = 6; Change: -0.08 [95%CI -0.22, 0.06]; N = 2), nor in interventions delivered in a group-based format (Post intervention: SMD -0.07 [95%CI -0.20, 0.06]; N = 4; Change: SMD -0.18 [95%CI -0.62, 0.27]; N = 4). Only two trials reporting post intervention outcomes used a combination of both individual and group-based sessions and found no evidence of an effect on emotional eating (Post intervention: SMD -0.21 [95%CI -0.99, 0.57]). One study reporting change outcomes used a combination of individual and

group-based sessions and found evidence of an effect in favour of the intervention on emotional eating (SMD -0.38 [95%CI -0.61, -0.14]).

Subgroup analyses by intervention delivery mode found no evidence of an effect in interventions delivered face-to-face (Post intervention: SMD -0.03 [95%CI -0.16, 0.09], N = 6; Change: SMD -0.22 [95%CI -0.56, 0.11]; N = 5). There was evidence of an effect on emotional eating in favour of the intervention in remote interventions reporting post intervention outcomes (Post intervention: SMD -0.33 [95%CI -0.65, -0.02]; N = 4) but not in remote interventions reporting change outcomes (Change: SMD -0.08 [95%CI -0.22, 0.06]; N = 2). Two trials that reported post intervention outcomes used a combination of face-to-face and remote approaches and found no evidence of an effect on emotional eating (SMD -0.21 [95%CI -0.99, 0.57]).

In subgroup analyses based on comparison intensity, there was no evidence of an effect on emotional eating in trials reporting post intervention outcomes where interventions were compared to inactive control groups (Post intervention: SMD -0.11 [95%CI -0.28, 0.07]; N = 11). Only one trial reporting post intervention outcomes compared interventions to an active control group, also finding no evidence of an effect (Post intervention: SMD -0.18 [95%CI -0.42, 0.06]). For change outcomes, there was evidence of an effect on emotional eating in favour of the intervention for trials comparing interventions to inactive control groups (Change: SMD -0.26 [95%CI -0.51, -0.02]; N = 3) but not for trials comparing interventions to active control groups (Change: SMD -0.06 [95%CI -0.43, 0.31]; N = 4).

Subgroup analyses by average baseline BMI found no evidence of an effect on post intervention emotional eating outcomes in both trials where the average baseline BMI of participants was below 35 (Post intervention: SMD -0.09 [95%CI -0.29, 0.10]; N = 7) and in those where it was above 35 (Post intervention: SMD -0.15 [95%CI -0.42, 0.12]; N = 5). For change outcomes, there was evidence of an effect on emotional eating in favour of the intervention in trials where the average baseline BMI was below 35 (SMD -0.34 [95%CI -0.59, -0.08]; N = 4) but not for those where the average baseline BMI was above 35 (SMD 0.11 [95%CI -0.10, 0.33]; N = 3).

6.2.1.1 Intervention type - Forest plots for impact on emotional eating by intervention type at end of intervention

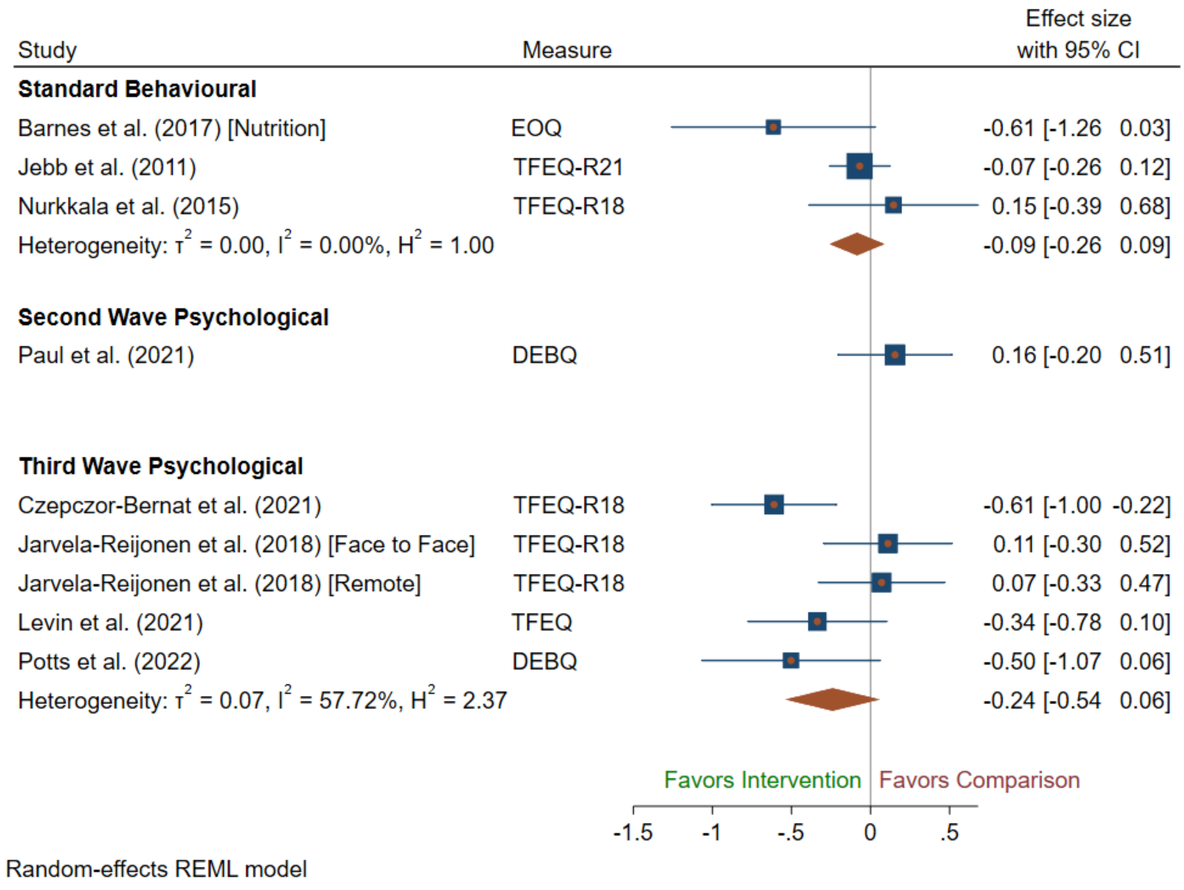

Figure S12 Post intervention emotional eating outcomes by intervention type at intervention end

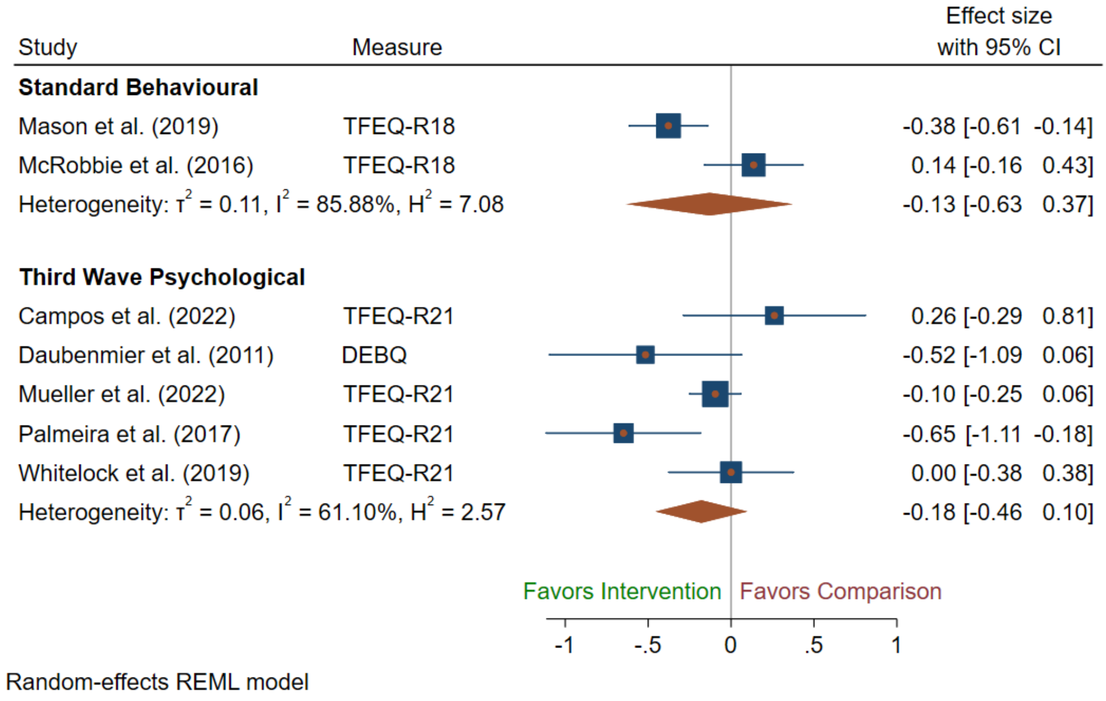

Figure S13 Change in emotional eating outcomes by intervention type at intervention end

6.2.1.2 Intervention duration - Forest plots for impact on emotional eating by intervention duration at end of intervention

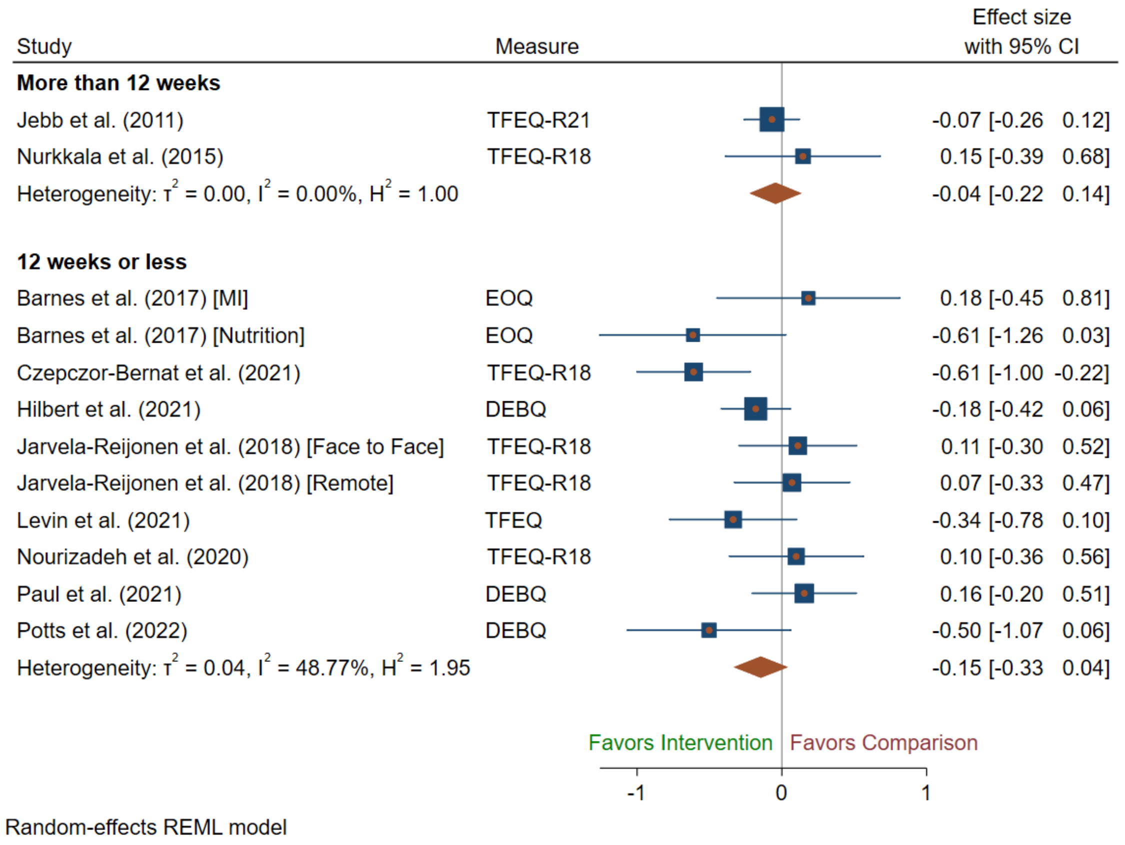

Figure S14 Post intervention emotional eating outcomes by intervention duration at intervention end

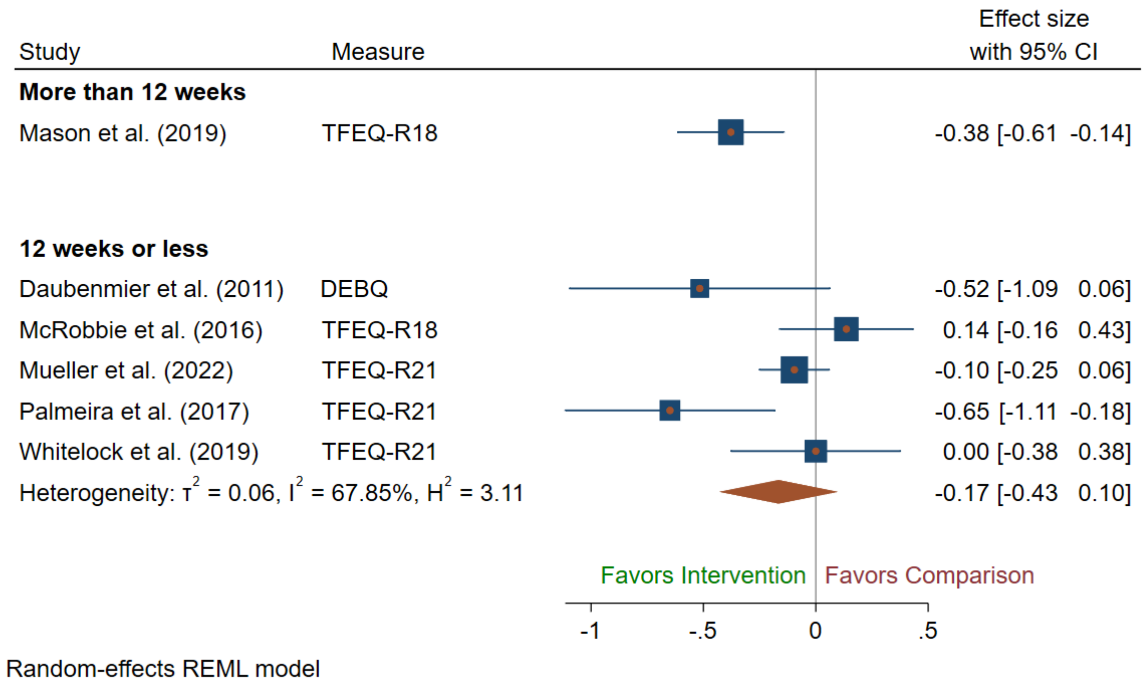

Figure S15 Change in emotional eating outcomes by intervention duration at intervention end

6.2.1.3 Intervention delivery format - Forest plots for impact on emotional eating by intervention delivery format at end of intervention

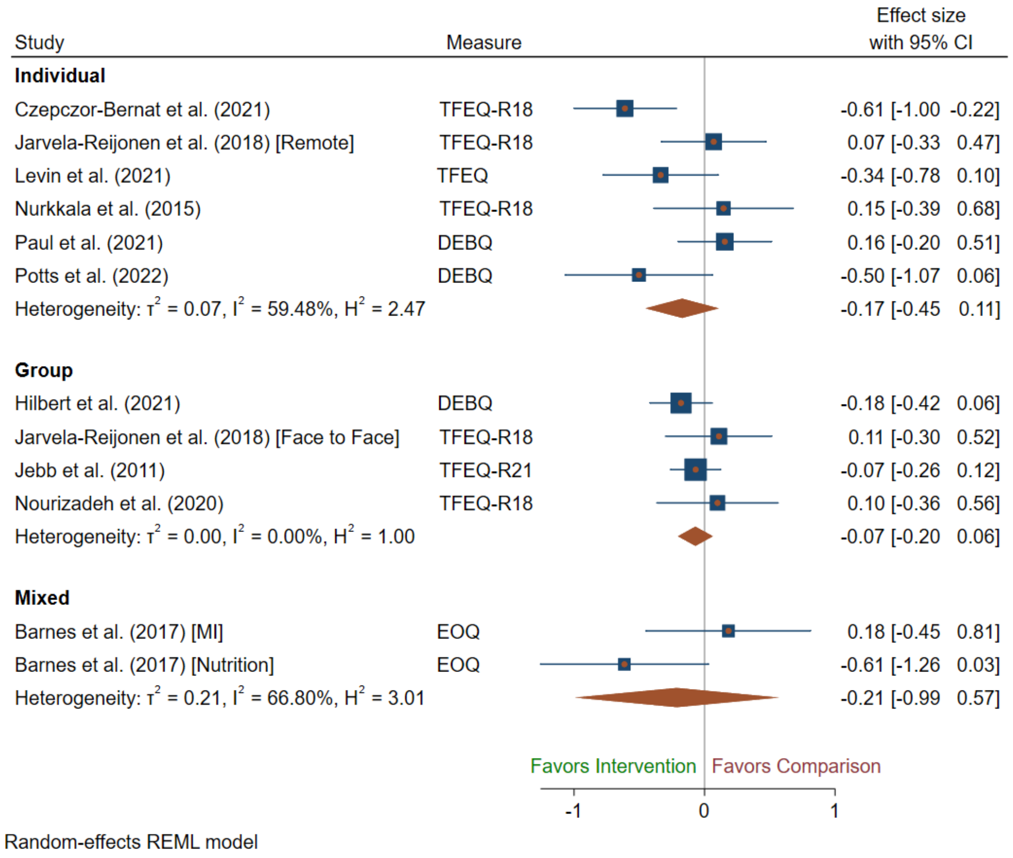

Figure S16 Post intervention emotional eating outcomes by intervention delivery format at intervention end

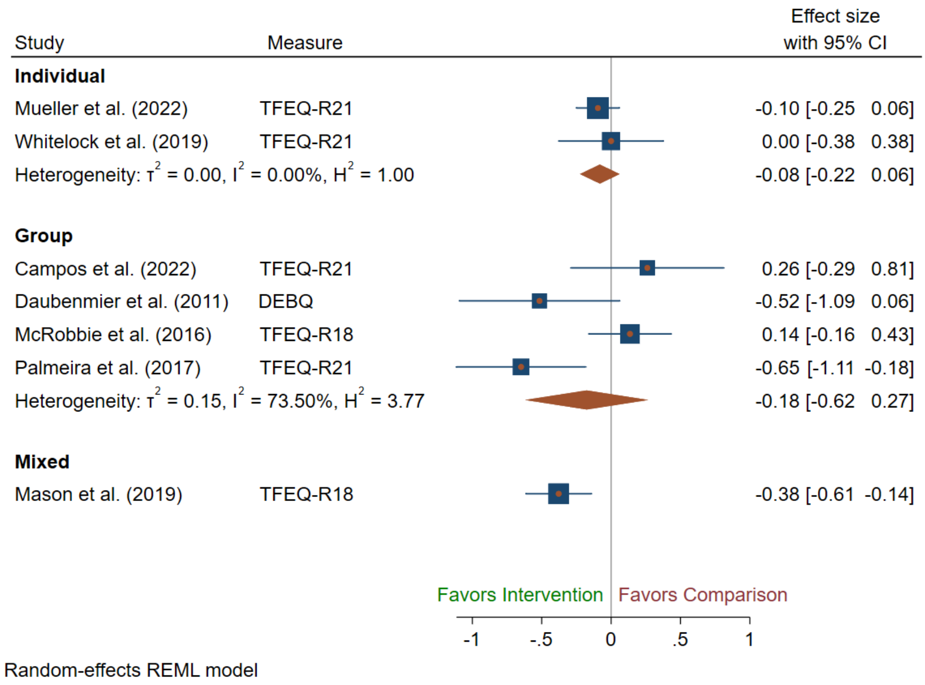

Figure S17 Change in emotional eating outcomes by intervention delivery format at intervention end

6.2.1.4 Intervention delivery mode - Forest plots for impact on emotional eating by Intervention Delivery Mode at end of intervention

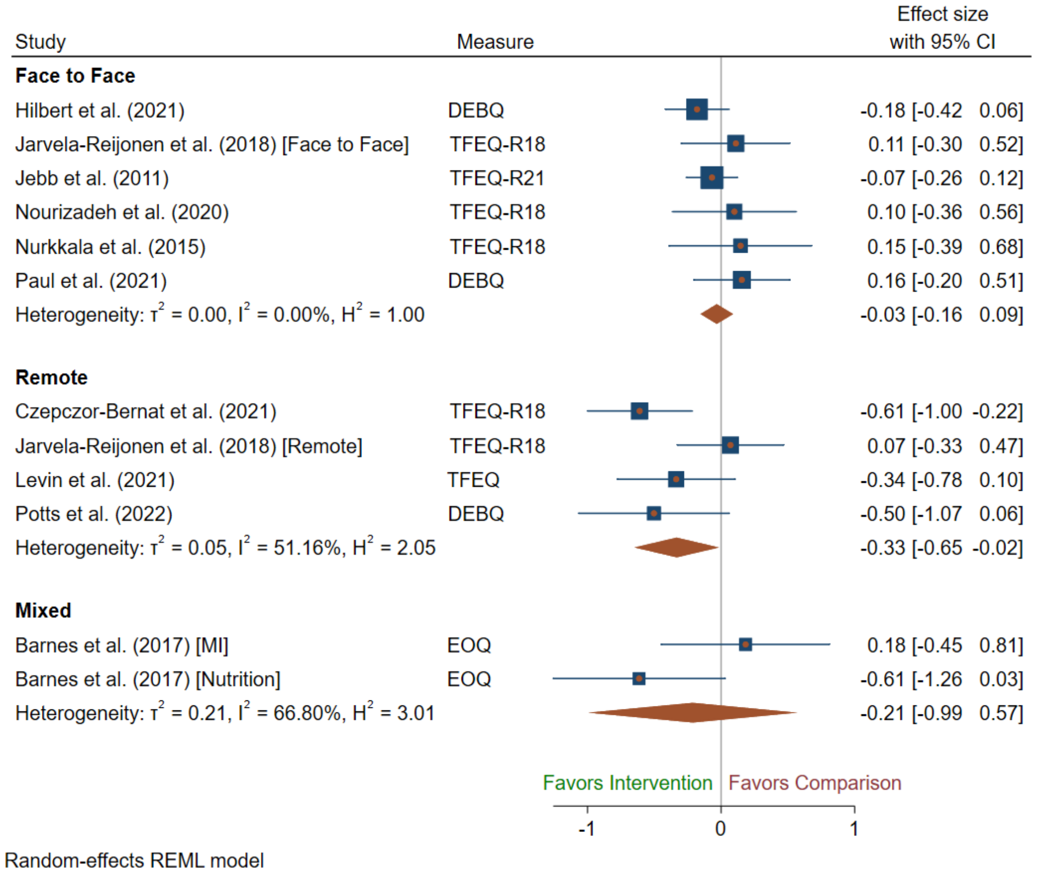

Figure S18 Post intervention emotional eating outcomes by intervention delivery mode at intervention end

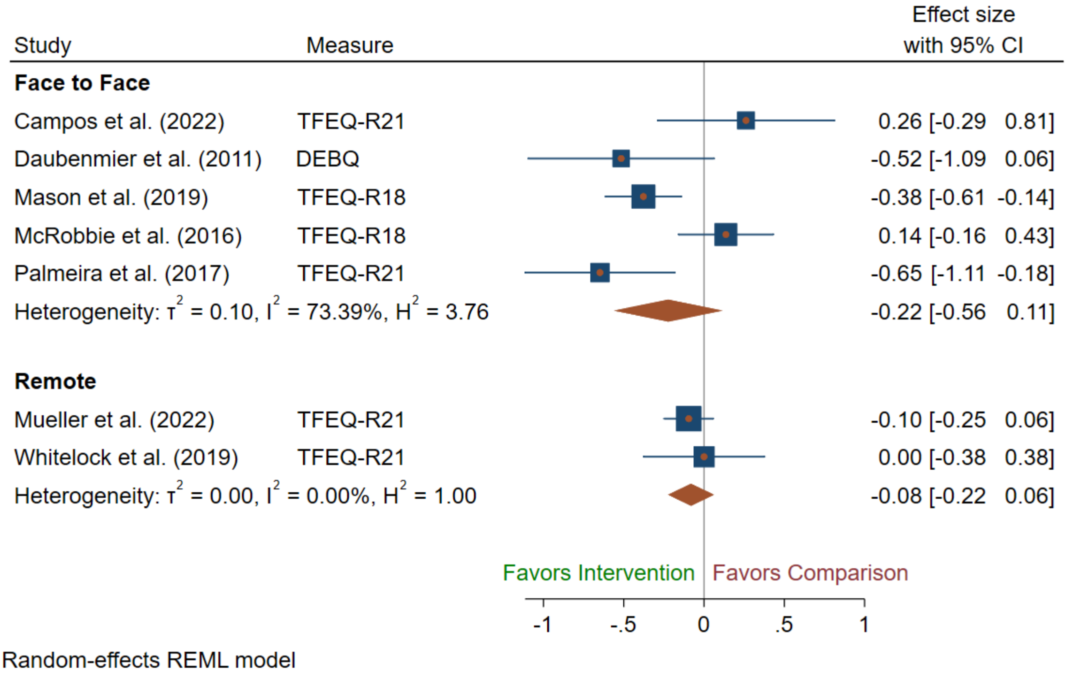

Figure S19 Change in emotional eating outcomes by intervention delivery mode at intervention end

6.2.1.5 Comparison Intensity - Forest plots for impact on emotional eating by comparison intensity at end of intervention

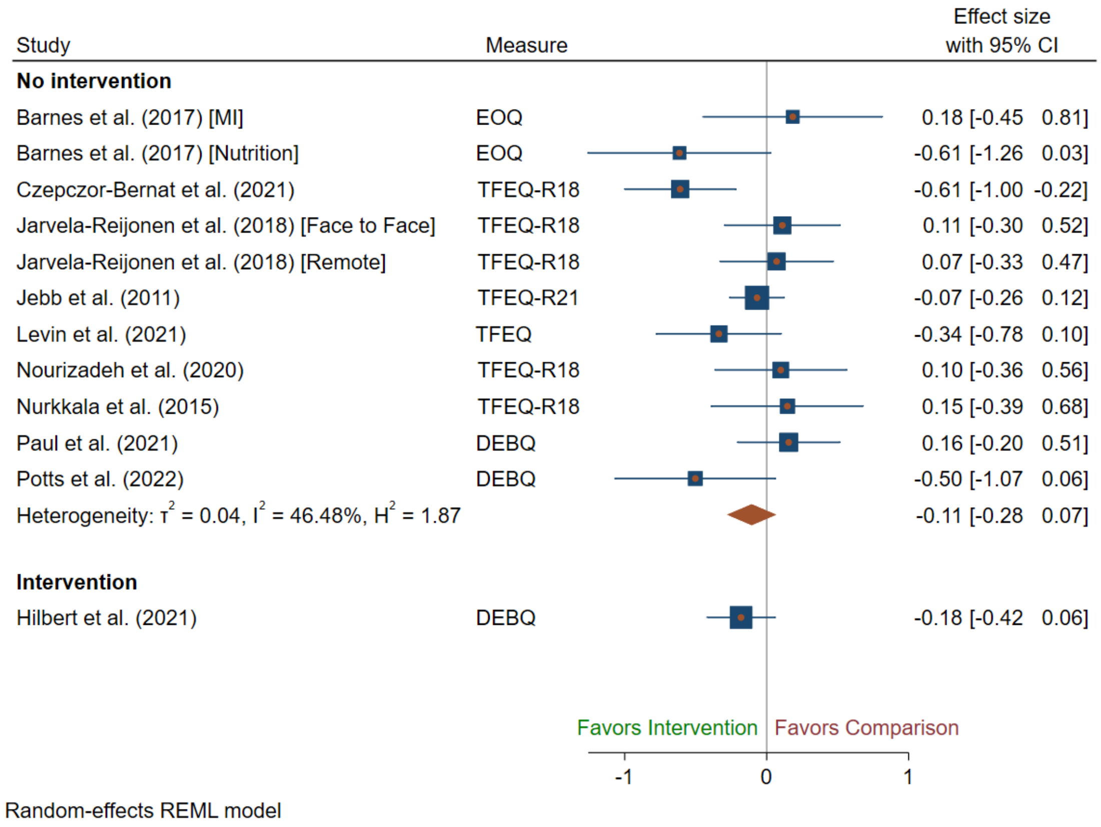

Figure S20 Post intervention emotional eating outcomes by comparison intensity at intervention end

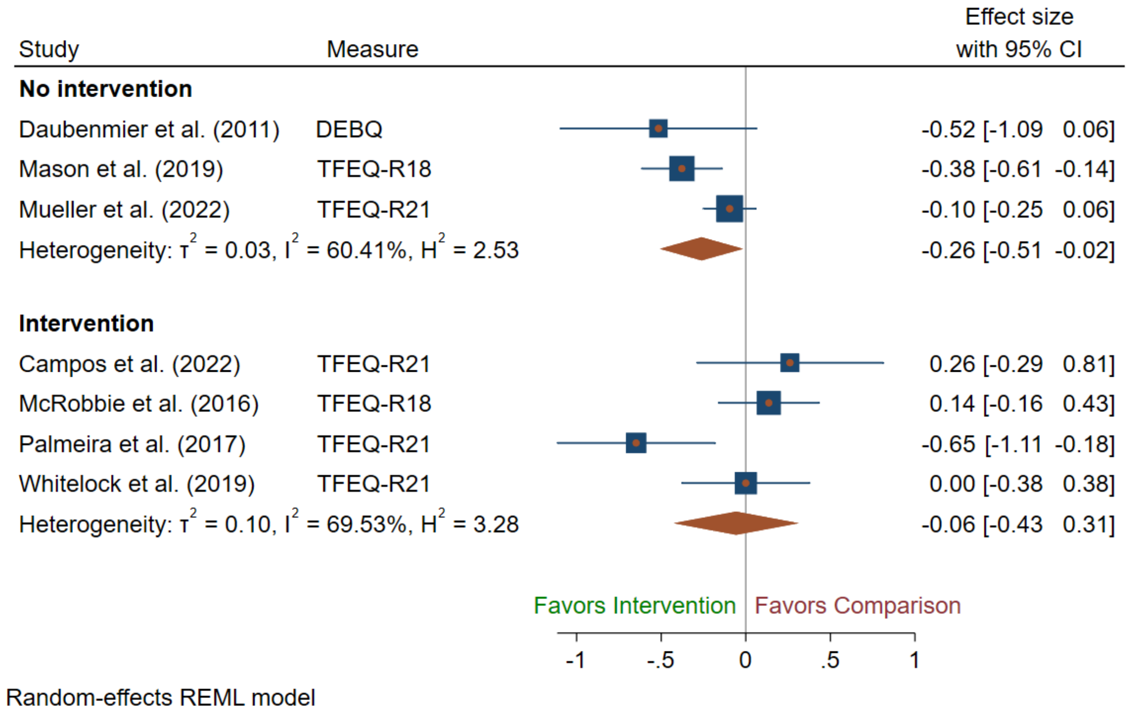

Figure S21 Change in emotional eating outcomes by comparison intensity at intervention end

6.2.1.6 Baseline BMI - Forest plots for impact on emotional eating by baseline BMI at end of intervention

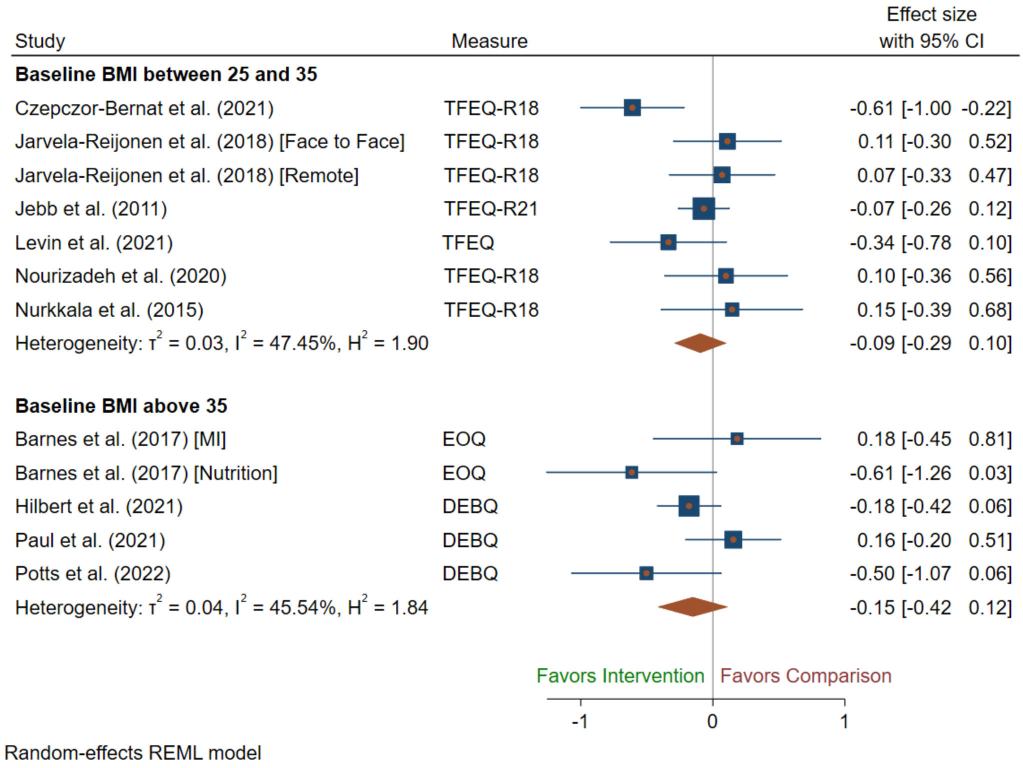

Figure S22 Post intervention emotional eating outcomes by baseline BMI at intervention end

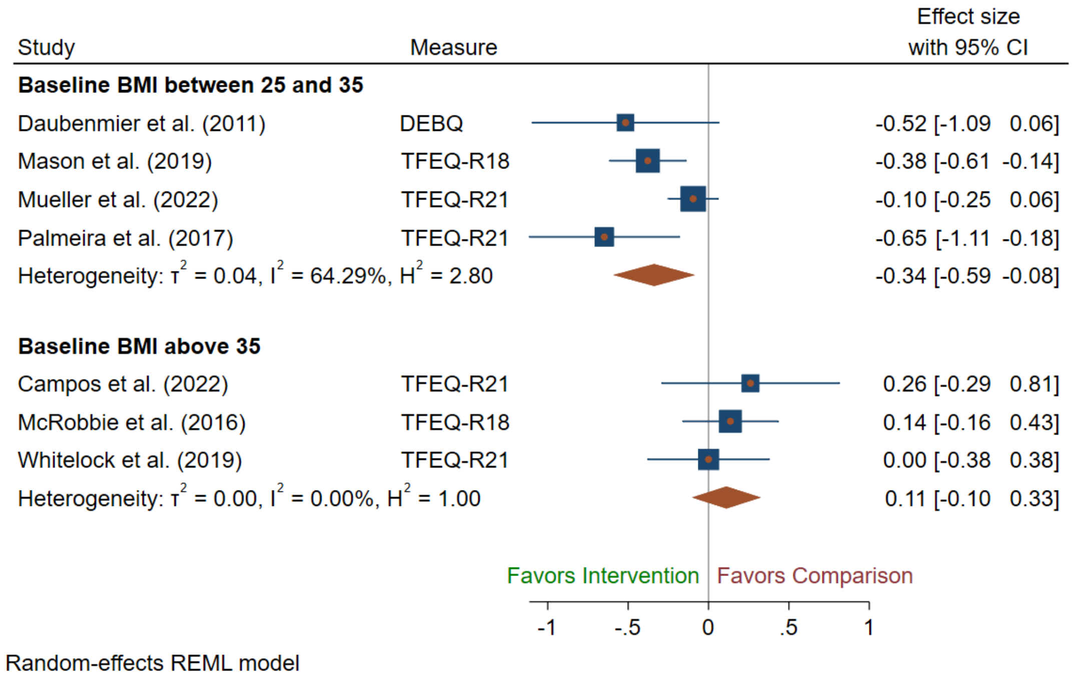

Figure S23 Change in emotional eating outcomes by baseline BMI at intervention end

6.2.2 Impact on emotional eating by subgroups at follow-up

Only two trials were included for emotional eating outcomes at follow-up (both post intervention and change outcomes). Thus, no subgroup analyses were performed.

6.3 External Eating

Due to insufficient contributing trials at both end of intervention (N = 3) and follow-up (N =1), no subgroup analyses were performed for external eating.

6.4 Hunger, hedonic

Due to insufficient contributing trial arms at both end of intervention (N = 3) and follow-up (N =2), no subgroup analyses were performed for hedonic hunger.

6.5 Hunger, susceptibility

Due to insufficient contributing trials at both end of intervention (N = 3) and follow-up (N =1), no subgroup analyses were performed for susceptibility to hunger.

6.6 Intuitive/ Mindful Eating

6.6.1 Impact on intuitive/ mindful eating by subgroups at end of intervention

Details on the impact of different subgroups on intuitive/mindful eating at intervention end are presented in Figures S24 to S29 for post intervention outcomes. No trials reported change outcomes for intuitive/mindful eating at intervention end. All four included intervention arms were classified as third wave behavioural interventions of 12 weeks duration or less, and the average BMI in all trial arms was between 25 and 35. These four trials found evidence for an effect on intuitive eating in favour of the intervention (SMD 0.23 [95%CI 0.04, 0.43]).

Subgroup analyses by intervention delivery format and mode found evidence of an effect on intuitive/mindful eating in favour of the intervention in trial arms where interventions were delivered to individuals and remotely (SMD 0.34 [95%CI 0.06, 0.62]; N = 2) but not in those where interventions were delivered to groups and face-to-face (SMD 0.12 [95%CI -0.15, 0.40]; N = 2).

In subgroup analyses based on comparison intensity, there was no evidence of an effect in trial arms where interventions were compared to inactive control groups (SMD 0.23 [95%CI -0.02, 0.48]). Only one trial compared the intervention to an active control finding no evidence of an effect on intuitive/mindful eating (SMD 0.24 [-0.15, 0.62]).

6.6.1.1 Intervention type - Forest plots for impact on intuitive/ mindful eating by Intervention type at end of intervention

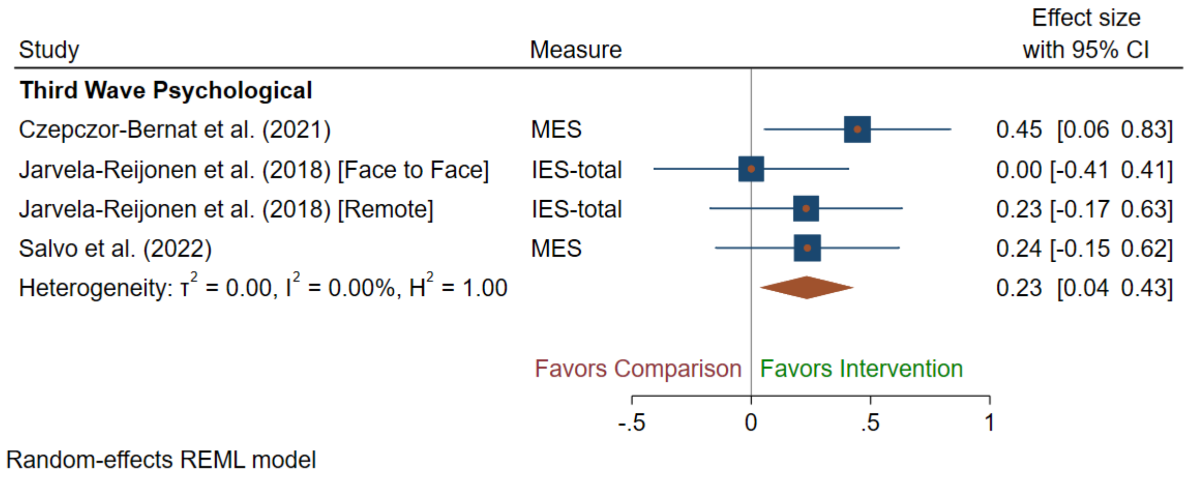

Figure S24 Post intervention intuitive/ mindful eating outcomes by intervention type at intervention end

6.6.1.2 Intervention duration - Forest plots for impact on intuitive/ mindful eating by intervention duration at end of intervention

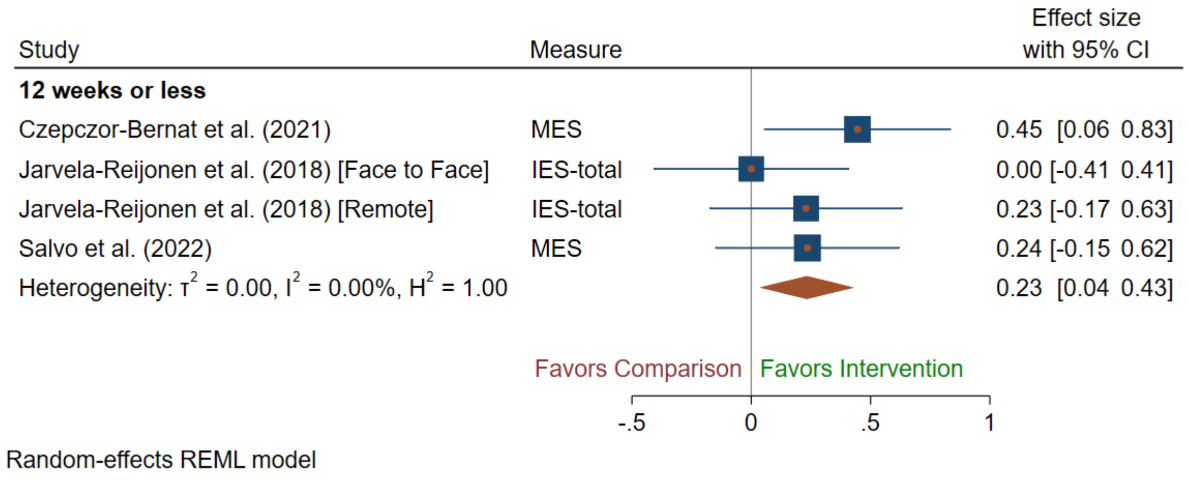

Figure S25 Post intervention intuitive/ mindful eating outcomes by intervention duration at intervention end

6.6.1.3 Intervention delivery format - Forest plots for impact on intuitive/ mindful eating by intervention delivery format at end of intervention

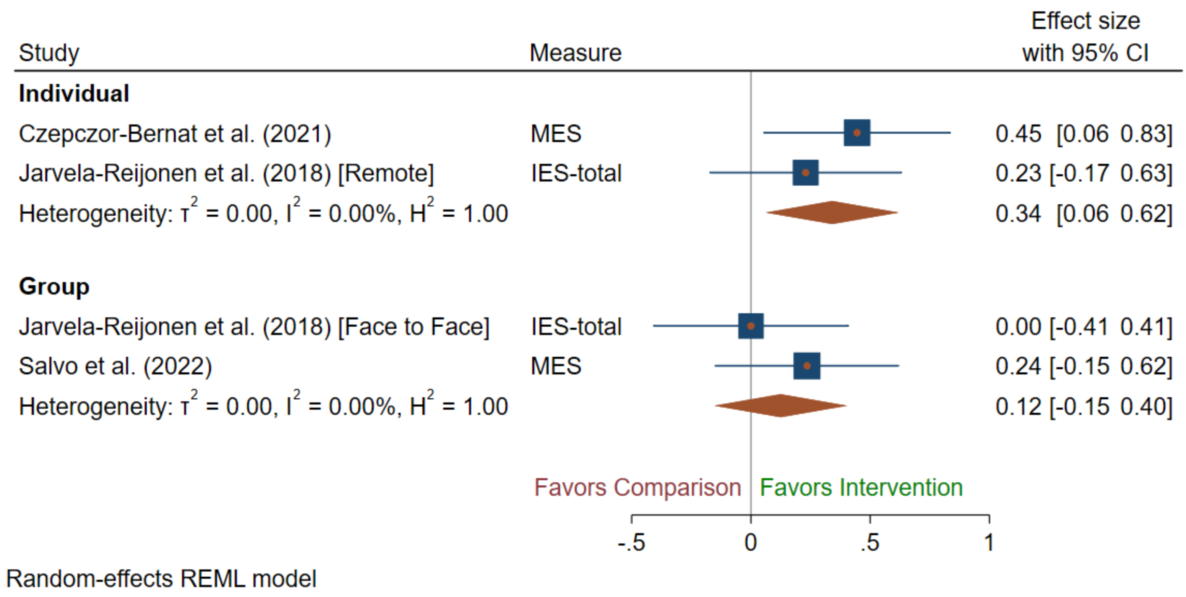

Figure S26 Post intervention intuitive/ mindful eating outcomes by intervention delivery format at intervention end

6.6.1.4 Intervention delivery mode - Forest plots for impact on intuitive/ mindful eating by intervention delivery mode at end of intervention

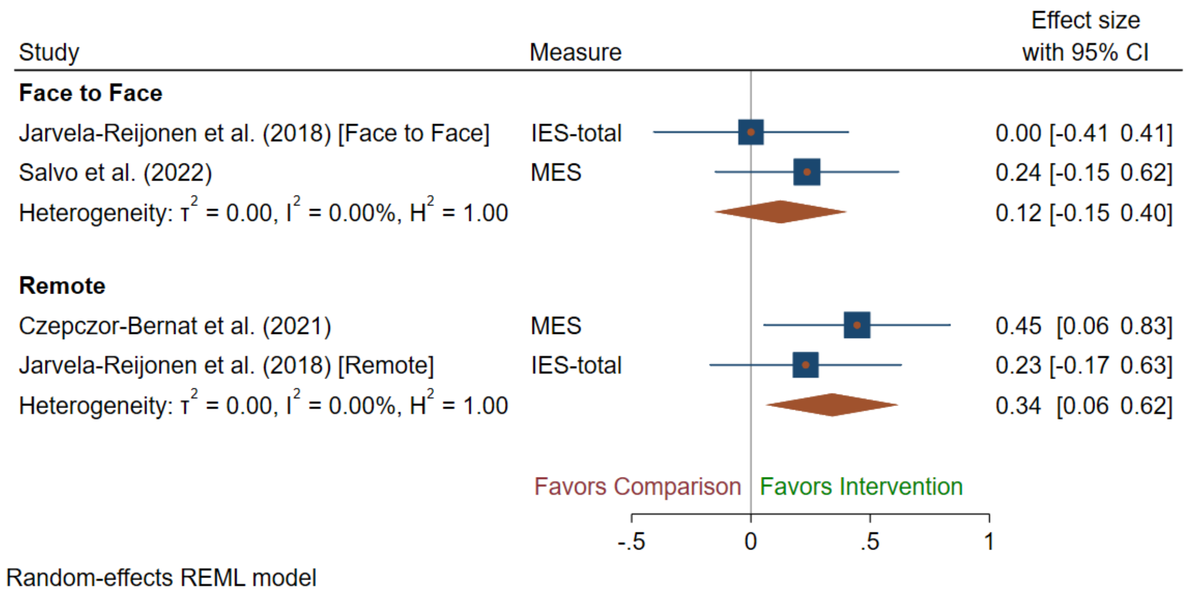

Figure S27 Post intervention intuitive/ mindful eating outcomes by intervention delivery mode at intervention end

6.6.1.5 Comparison intensity - Forest plots for impact on intuitive/ mindful eating by comparison intensity at end of intervention

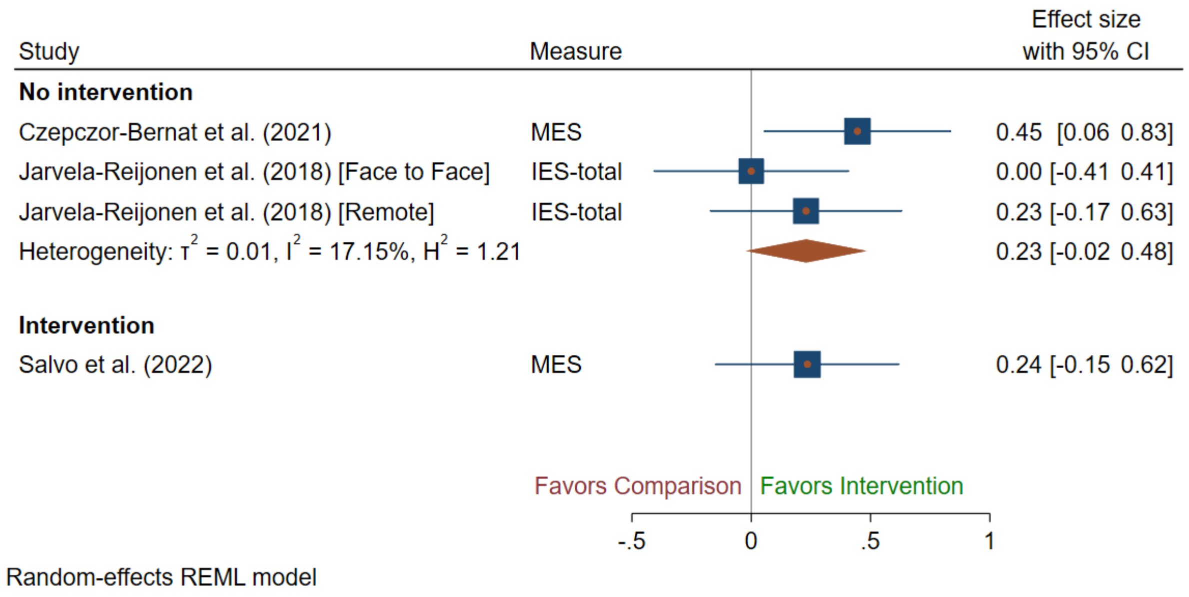

Figure S28 Post intervention intuitive/ mindful eating outcomes by comparison intensity at intervention end

6.6.1.6 Baseline BMI - Forest plots for impact on intuitive/ mindful eating by baseline BMI at end of intervention

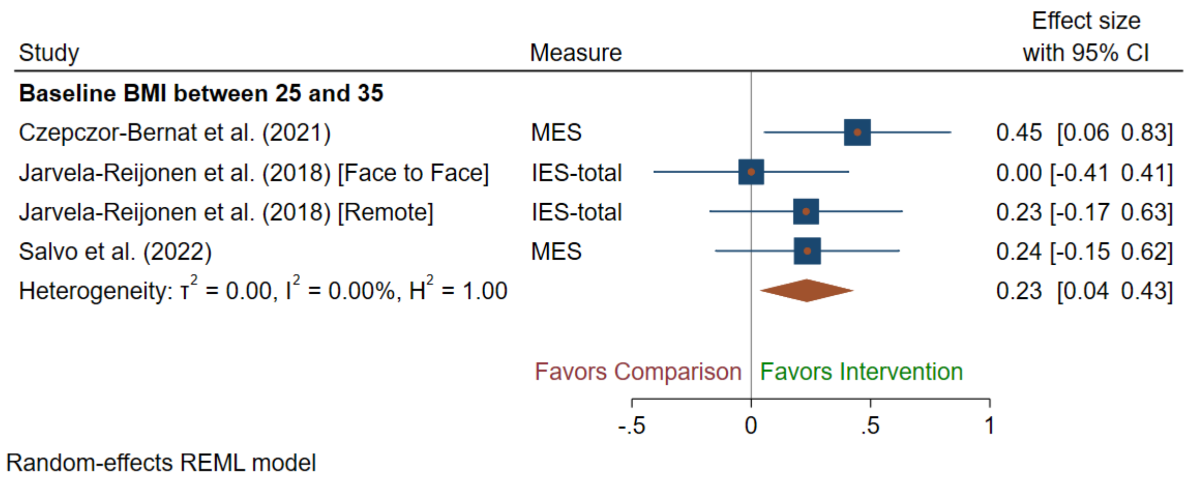

Figure S29 Post intervention intuitive/ mindful eating outcomes by baseline BMI at intervention end

6.6.2 Impact on intuitive/ mindful eating by subgroups at follow-up

No trials reported intuitive/mindful eating outcomes at the eligible follow-up (both post intervention and change outcomes).

6.7 Restraint

6.7.1 Impact on restraint by subgroups at end of intervention

Details on the impact of different subgroups on restraint at intervention end are presented in Figures S30, S32, S34, S36, S38 and S40 for post intervention outcomes and in Figures S31, S33, S35, S37, S39 and S41 for change outcomes.

Subgroup analyses by intervention type found evidence of an effect on restraint in favour of the intervention in trial arms classified as standard behavioural interventions (Post intervention: SMD 0.44 [95%CI 0.19. 0.70]; N = 6; Change: SMD 0.72 [95%CI 0.37, 1.07]; N = 4). There was no evidence of an effect on restraint in trial arms classified as second wave cognitive behavioural therapy based interventions (Post intervention: SMD 0.45 [95%CI -0.48, 1.38]; N = 2) nor in trial arms classified as third wave cognitive behavioural therapy based interventions (Post intervention: SMD 0.08 [95%CI -0.16, 0.32]; N = 3; Change: SMD 0.08 [95%CI -0.32, 0.48]; N = 4).

In subgroup analyses based on intervention duration, there was evidence of an effect on restraint in favour of the intervention in trial arms of interventions lasting longer than 12 weeks reporting post intervention outcomes (Post intervention: SMD 0.58 [95%CI 0.25-0.91]; N = 4; I^2 =79.18%), but not in those reporting change outcomes (Change: SMD 0.46 [95%CI -0.28, 1.20]; N = 3). There was some evidence to suggest an effect on restraint in trial arms of interventions of 12 weeks duration or less (Post intervention: SMD 0.19 [95%CI -0.02, 0.39]; N = 9; Change: SMD 0.34 [95%CI -0.01, 0.68]; N = 5).

Subgroup analyses by intervention delivery format found no evidence of an effect of interventions on restraint in both trials of individual-based (Post intervention: SMD 0.39 [95%CI -0.03, 0.81]; N = 5; Change: SMD 0.43 [95%CI -0.25, 1.12]; N = 3) and group-based interventions (Post intervention: SMD 0.38 [95%CI 0.10, 0.66]; N = 7; Change: SMD 0.31 [95%CI -0.21, 0.83]; N = 4). Only two trials reporting post intervention outcomes combined individual-and group-based formats and found no evidence of an effect on restraint (Post intervention: SMD 0.16 [95%CI -0.29. 0.60). Only one trial reported change outcomes and combined individual and group-based formats and found evidence of an effect on restraint in favour of the intervention (Change: SMD 0.58 [95%CI 0.35, 0.82]).

Subgroup analyses by intervention delivery mode found evidence of an effect on restraint in favour of the intervention in trials reporting post intervention outcomes for face-to-face interventions (Post intervention: SMD 0.39 [95%CI 0.13, 0.64]; N = 9), but not in trials reporting change outcomes (Change: SMD 0.27 [95%CI -0.09, 0.62]; N = 4). There was no evidence of an effect on restraint in remote interventions (Post intervention: SMD 0.37 [95%CI -0.20, 0.95]; N = 3; Change: SMD 0.14 [95%CI -0.52, 0.81]; N = 2). There was evidence of an effect on restraint in favour of the intervention in interventions using a combination of remote and face-to-face approaches for change outcomes (Change: SMD 1.01 [95%CI 0.86, 1.17]; N = 2], but not for post intervention outcomes (Post intervention: SMD 0.16 [95%CI -0.29, 0.60]; N = 2).

Subgroup analyses by comparison intensity found evidence of an effect on restraint in favour of the intervention in trials comparing interventions to inactive control groups (Post intervention: SMD 0.39 [95%CI 0.18, 0.60]; N = 13; Change: SMD 0.51 [95%CI 0.38, 0.63]; N = 4). There was no evidence of an effect on restraint in trials comparing interventions to active control groups and reporting change outcomes (Change: SMD 0.23 [95%CI -0.38, 0.84]; N = 4). There was only one trial with an active comparison group for post intervention outcomes. This trial did also not find evidence of an effect on restraint (Post intervention: SMD 0 [95%CI -0.24, 0.24]).

In subgroup analyses based on baseline BMI, there was evidence of an effect on restraint in favour of the intervention in intervention arms of trials with an average baseline BMI of below 35 (Post intervention: SMD 0.47 [95%CI 0.22, 0.71]; N = 10; Change: SMD 0.51 [0.38, 0.63]; N = 4) but not in those from trials with a baseline BMI above 35 (Post intervention: SMD 0.03 [95%CI -0.15, 0.21]; N = 4; Change: SMD 0.23 [95%CI -0.38, 0.84]; N = 4).

6.7.1.1 Intervention type - Forest plots for impact on restraint by intervention type at end of intervention

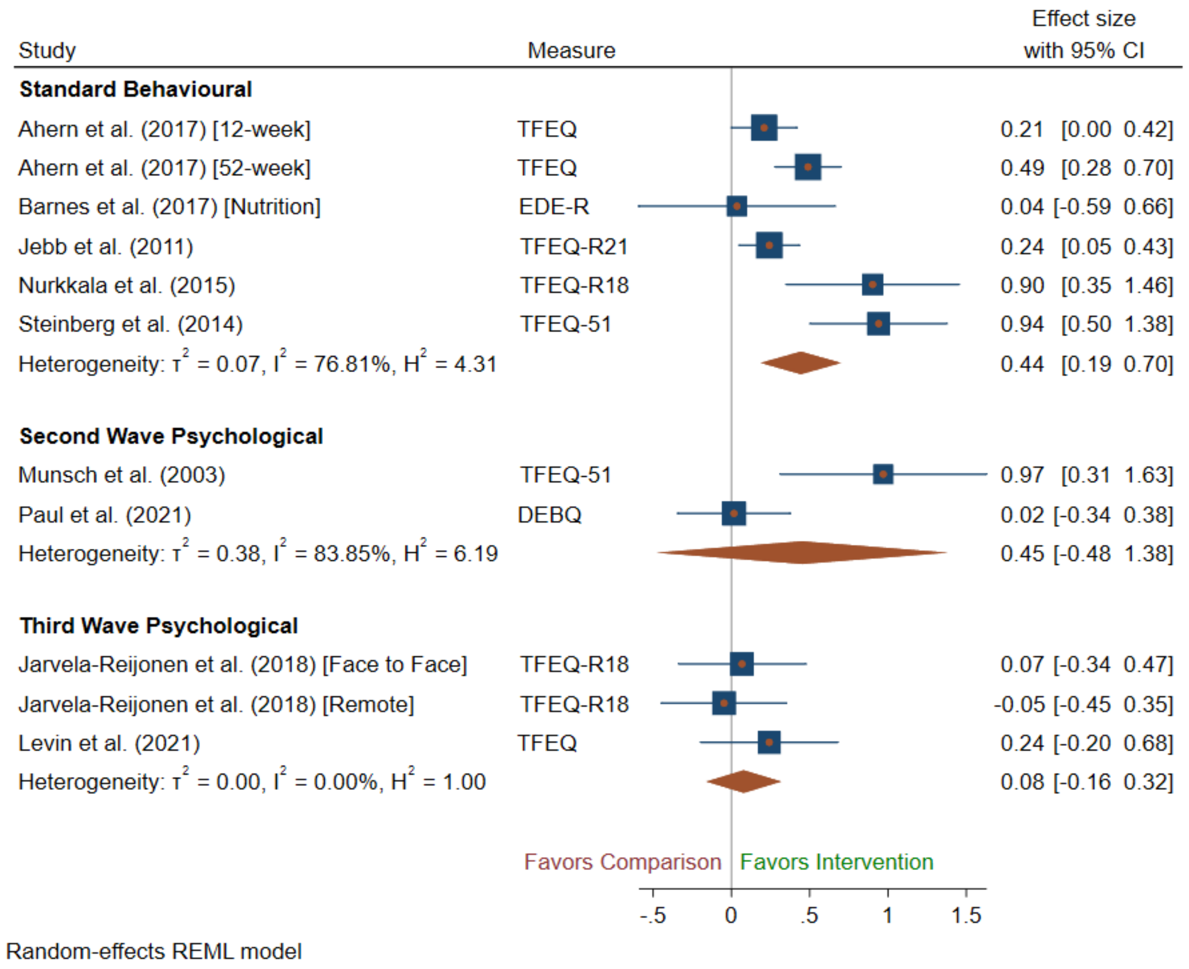

Figure S30 Post intervention restraint outcomes by intervention type at intervention end

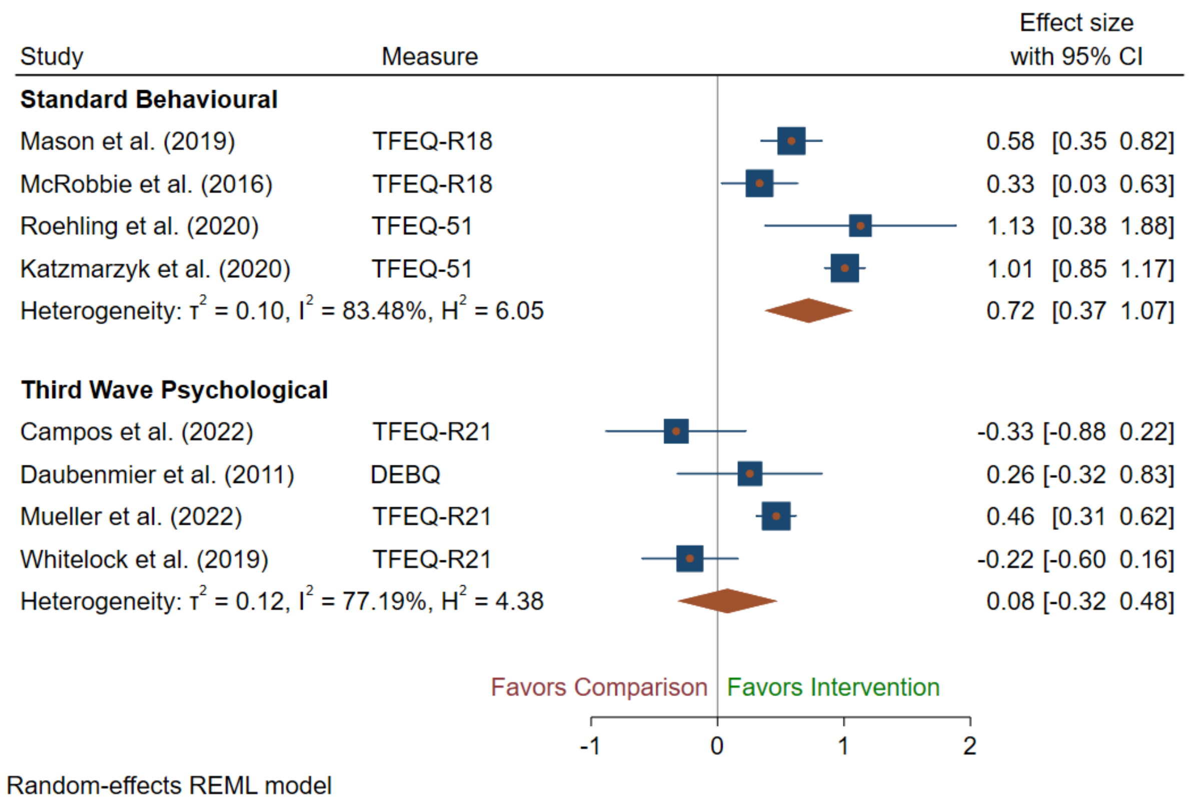

Figure S31 Change in restraint outcomes by intervention type at intervention end

6.7.1.2 Intervention duration - Forest plots for impact on restraint by intervention duration at end of intervention

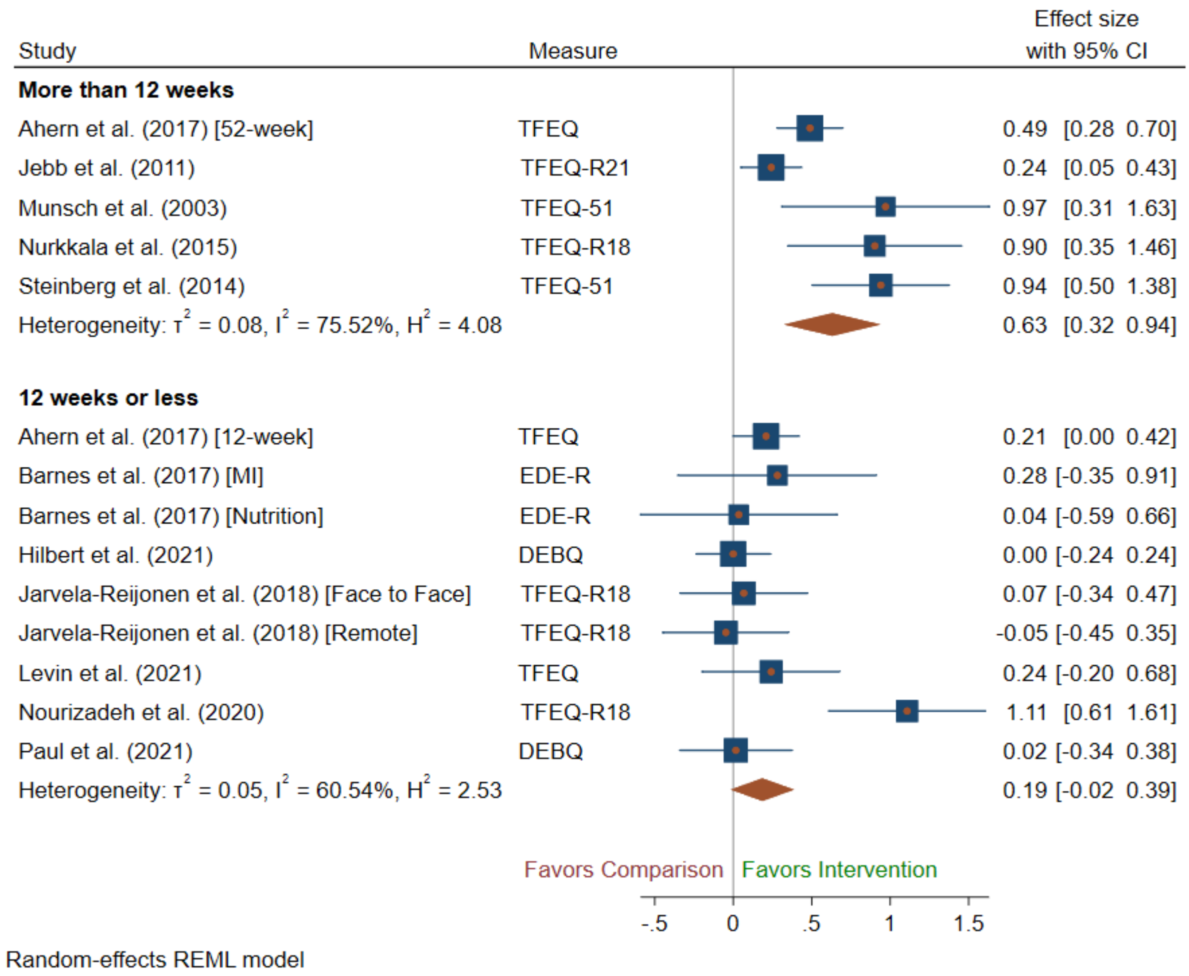

Figure S32 Post intervention restraint outcomes by intervention duration at intervention end

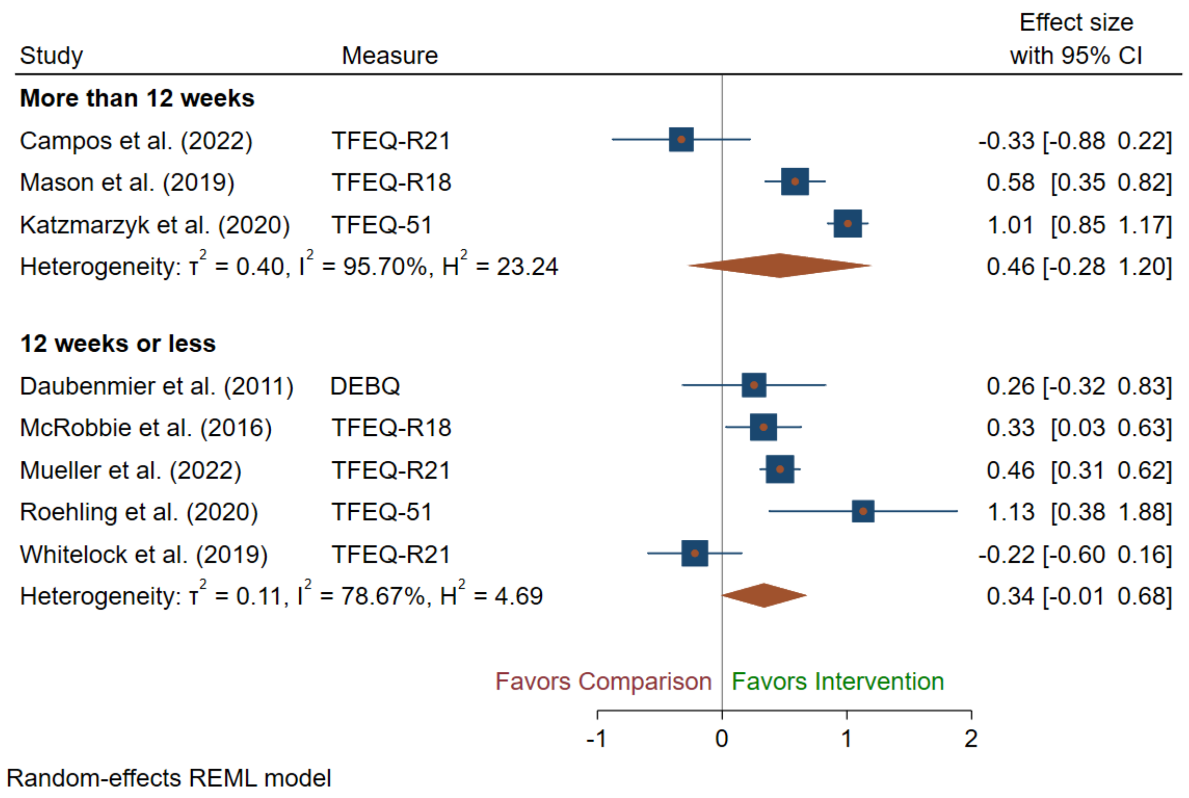

Figure S33 Change in restraint outcomes by intervention duration at intervention end

6.7.1.3 Intervention delivery format – Forest plots for impact on restraint by intervention delivery format at end of intervention

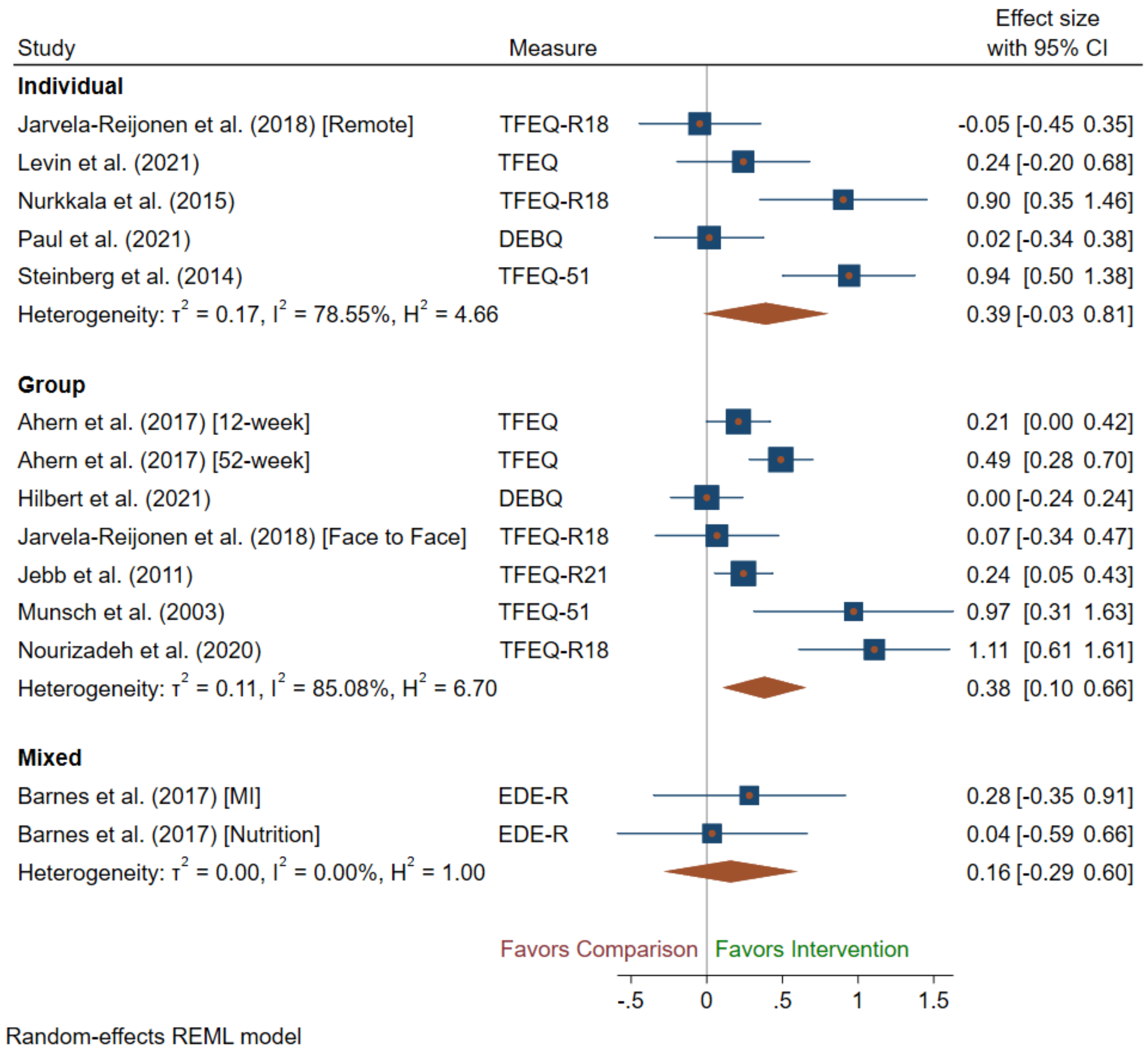

Figure S34 Post intervention restraint outcomes by intervention delivery format at intervention end

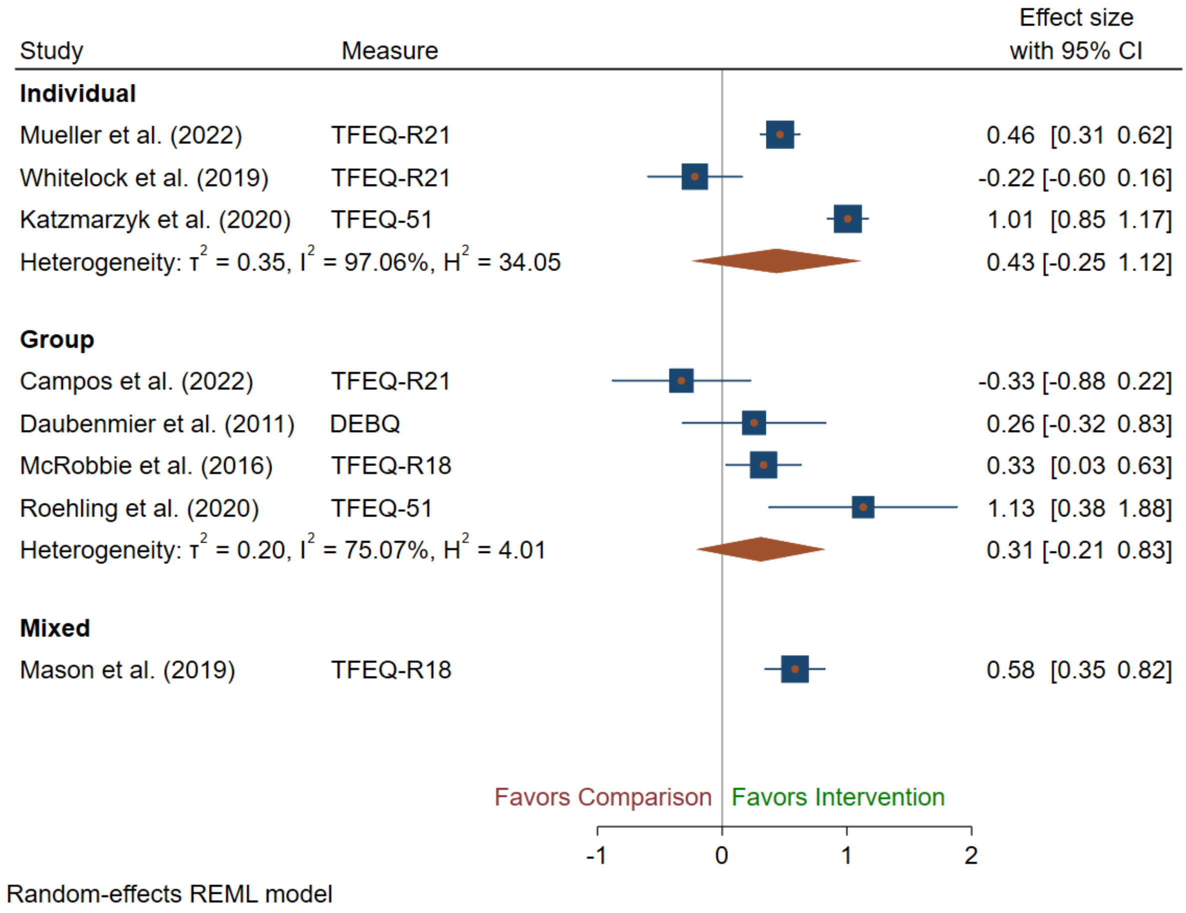

Figure S35 Change in restraint outcomes by intervention delivery format at intervention end

6.7.1.4 Intervention delivery mode – Forest plots for impact on restraint by intervention delivery mode at end of intervention

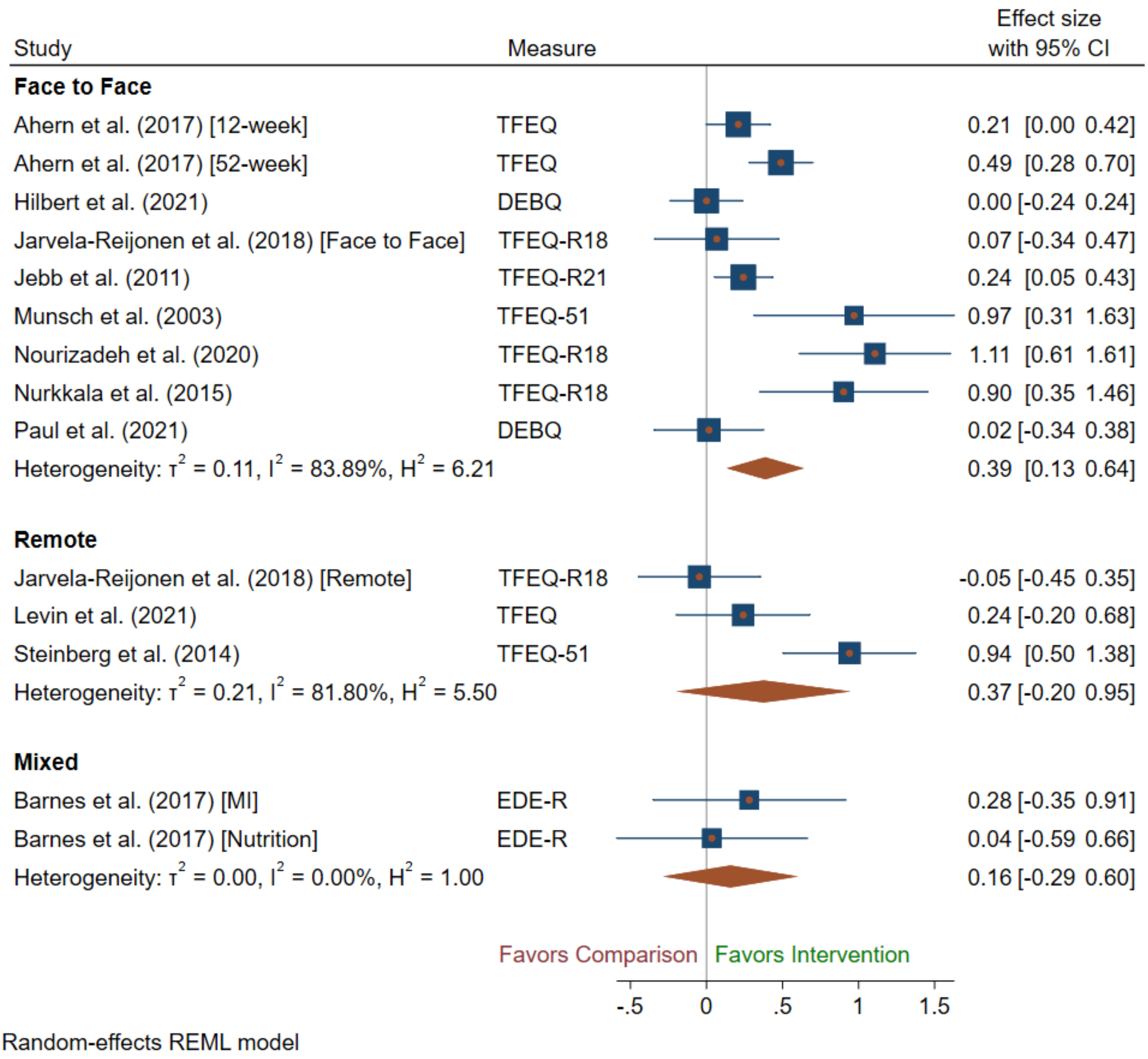

Figure S36 Post intervention restraint outcomes by intervention delivery mode at intervention end

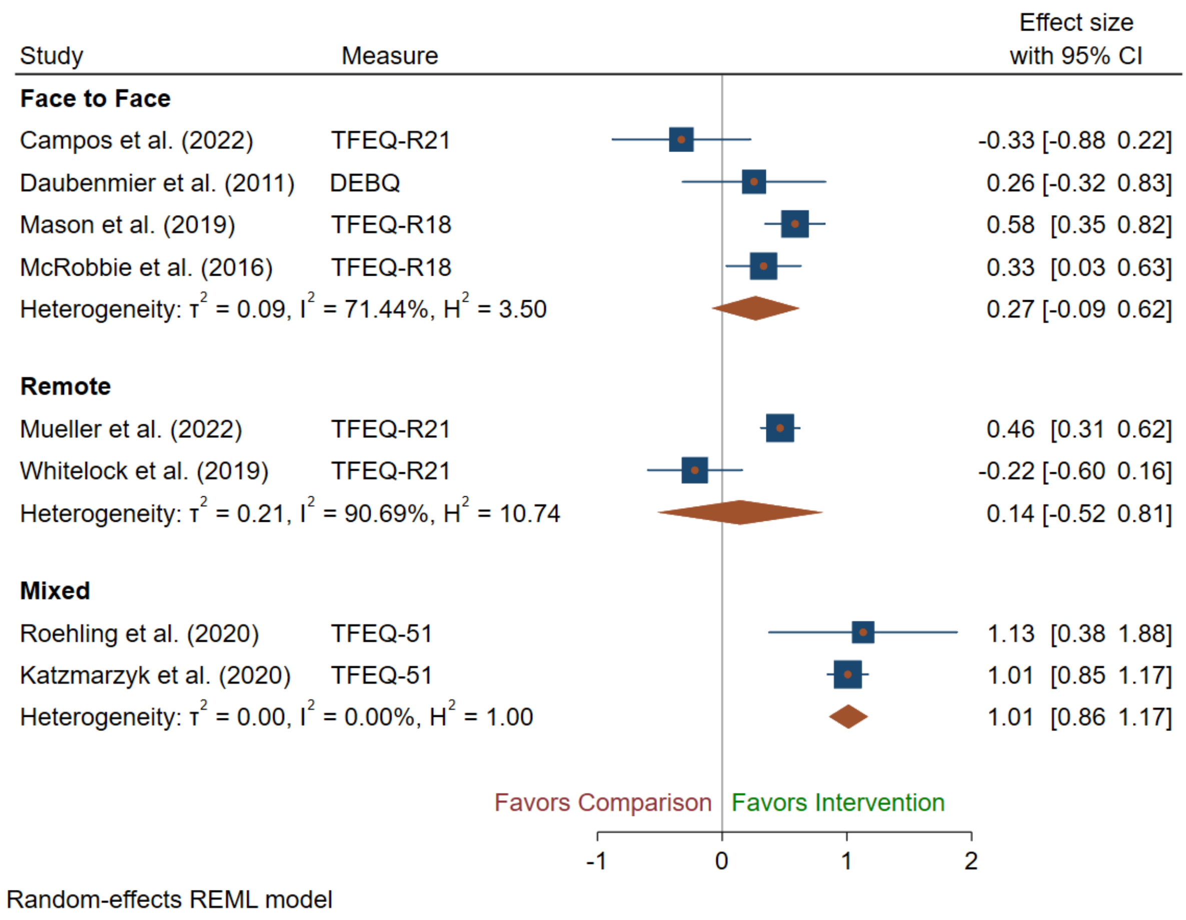

Figure S37 Change in restraint outcomes by intervention delivery mode at intervention end

6.7.1.5 Comparison intensity – Forest plots for impact on restraint by comparison intensity at intervention end

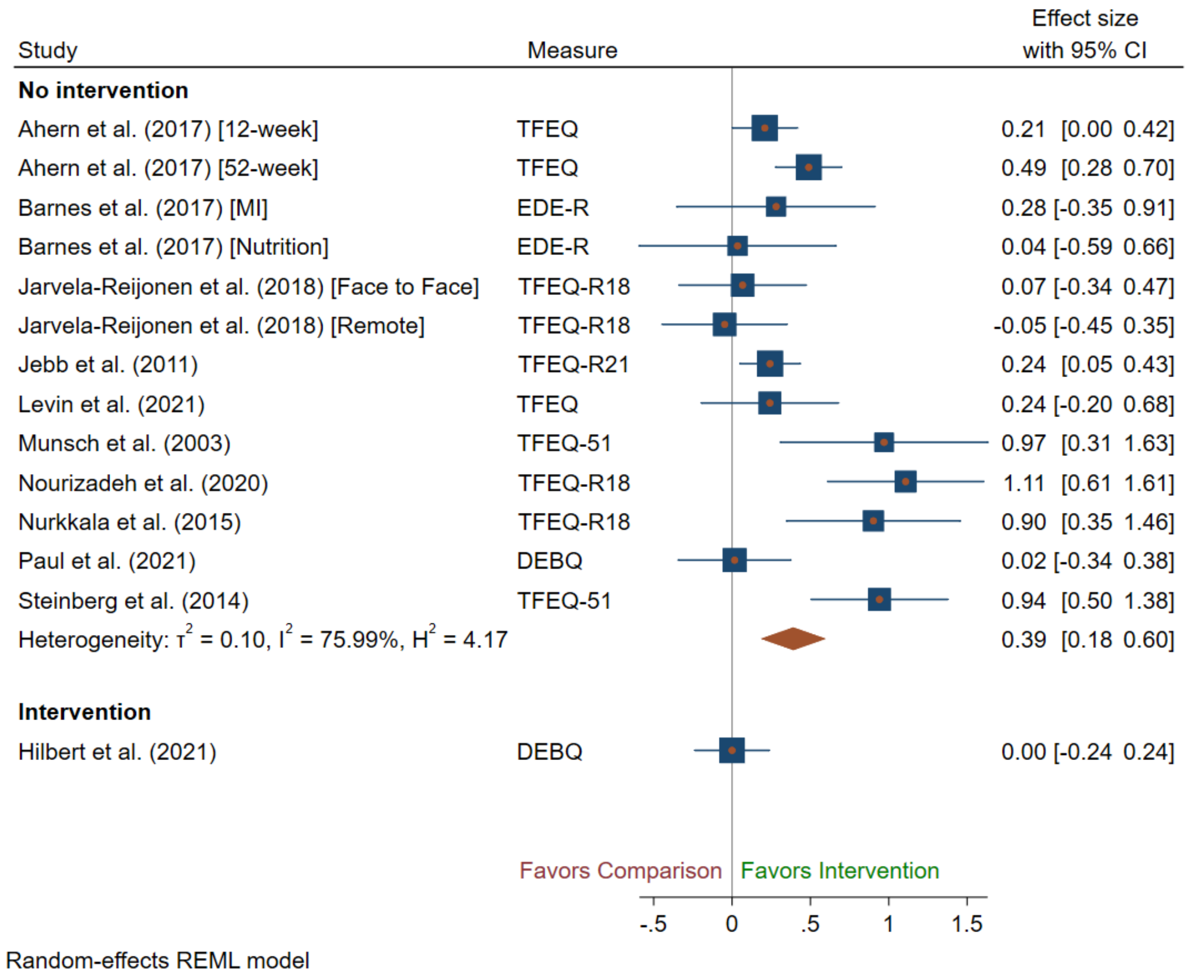

Figure S38 Post intervention restraint outcomes by comparison intensity at intervention end

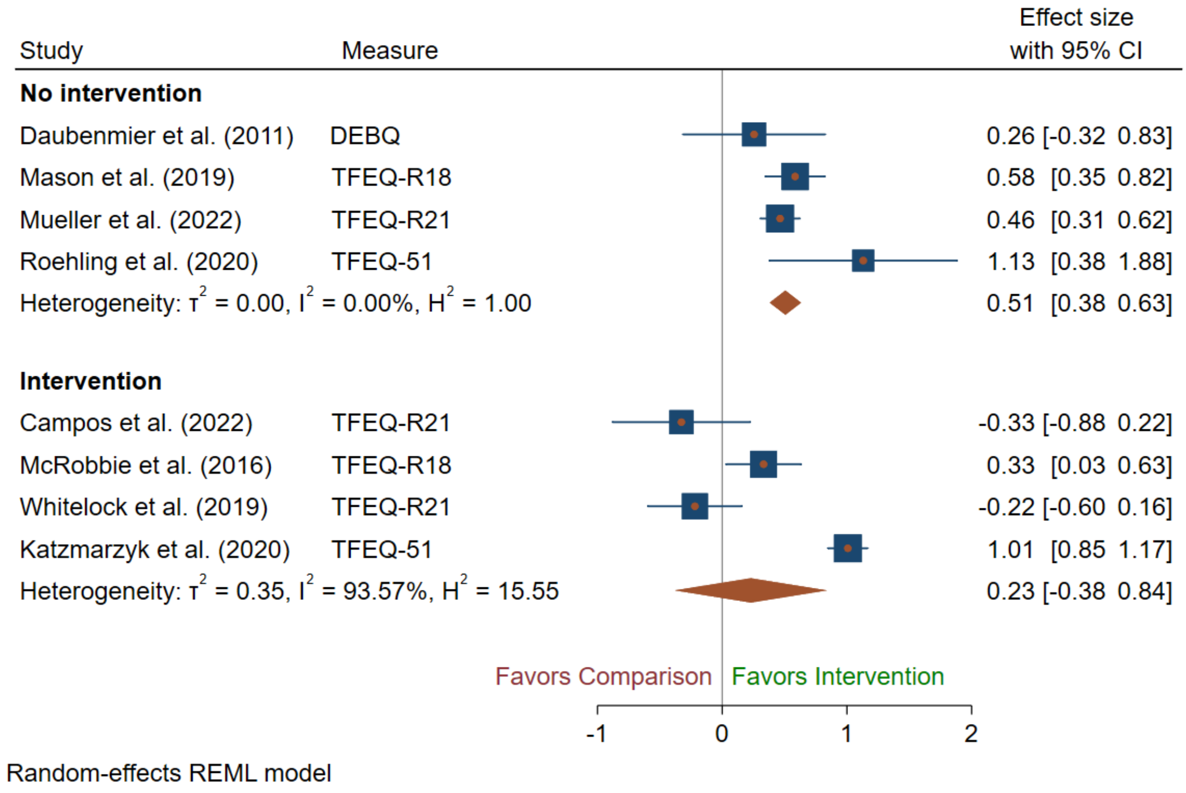

Figure S39 Change in restraint outcomes by comparison intensity at intervention end

6.7.1.6 Baseline BMI – Forest plots for impact on restraint by baseline BMI at end of intervention

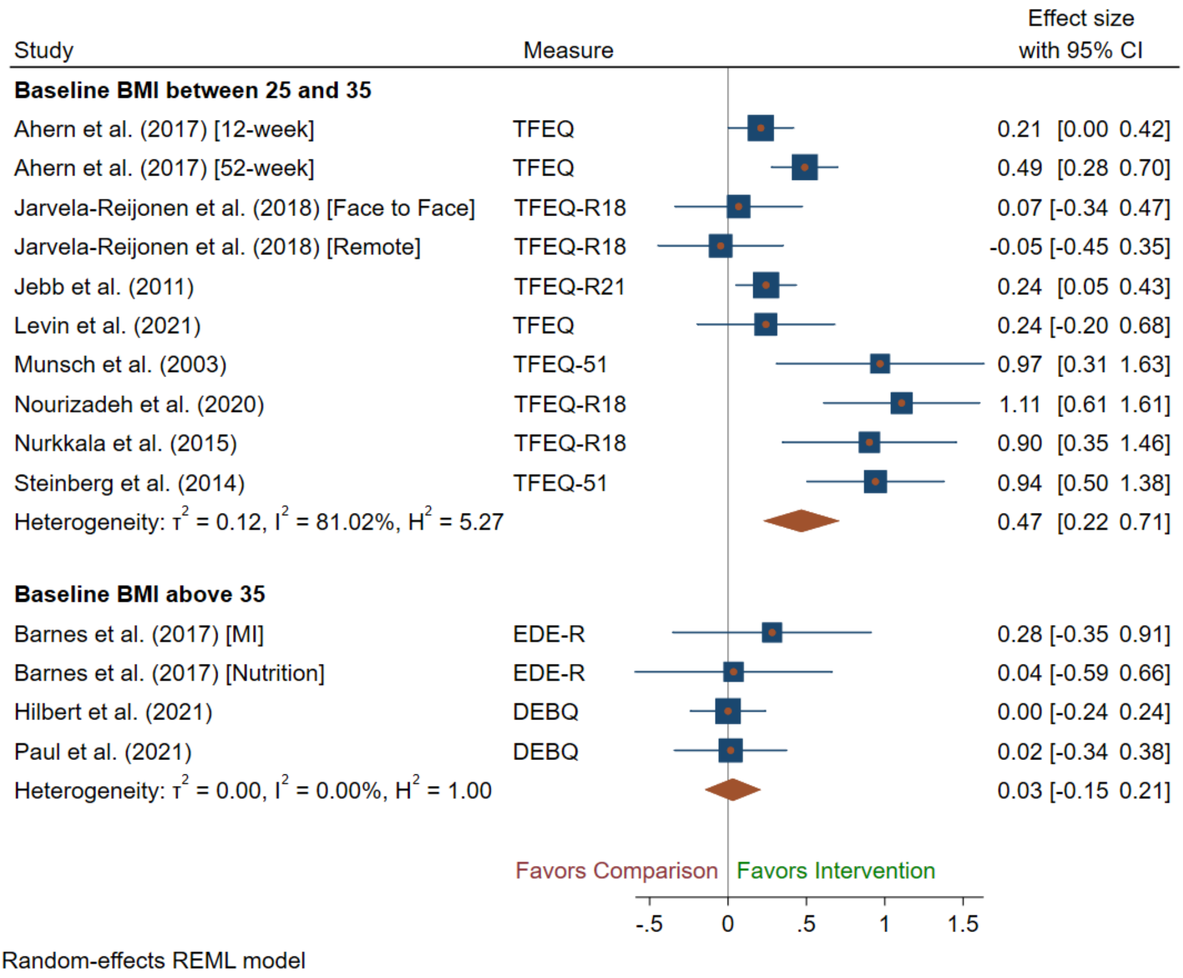

Figure S40 Post intervention restraint outcomes by baseline BMI at intervention end

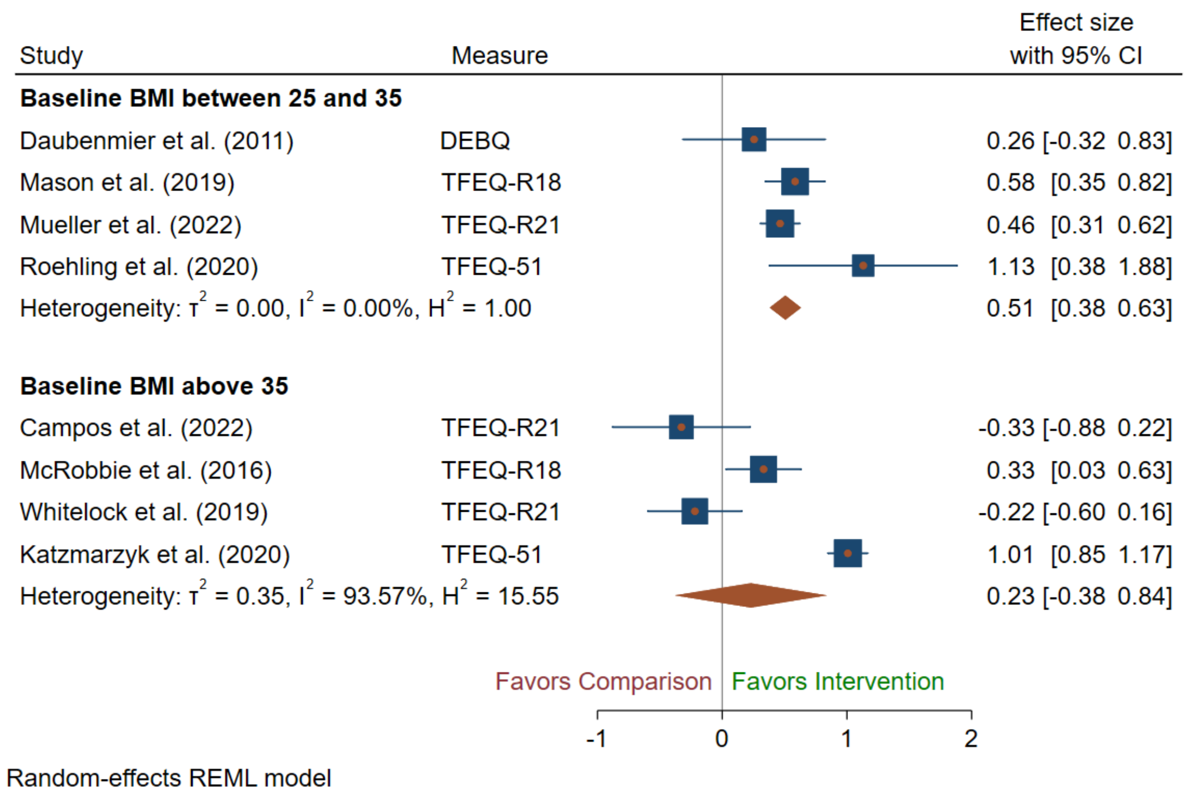

Figure S41 Change in restraint outcomes by baseline BMI at intervention end

6.7.2 Impact on restraint by subgroups at follow-up

Details on the impact of different subgroups on restraint at the 12-month follow-up are presented in Figures S42 to S47 for post intervention outcomes. There were only two trials reporting change outcomes at follow-up, hence, no subgroup analyses were conducted for that outcome.

In subgroup analyses based on intervention type, there was evidence of an effect on restraint in favour of the intervention in trial arms classified as standard behavioural interventions (SMD 0.17 [95% CI 0.03, 0.32]; N = 2) at 12 months after intervention end. Only one trial reported the effects of trial arms classified as second wave cognitive behavioural therapy-based interventions at follow-up, also finding evidence of an effect on restraint in favour of the intervention (SMD 0.89 [95%CI 0.12, 1.65]). No trials of third wave psychological interventions reported restraint at the eligible follow-up time point.

Subgroup analyses by intervention duration found no evidence of an effect in both trial arms of interventions with a duration of 12 weeks or less (SMD 0.12 [95%CI -0.04, 0.28]; N = 2) and in those of interventions with longer duration (SMD 0.45 [95%CI -0.16, 1.06], N = 2).

All trials reporting post intervention outcomes of restraint at follow-up were group-based and delivered face to face. These trial arms found evidence of an effect on restraint in favour of the interventions (SMD 0.18 [95% CI 0.05, 0.30]; N = 4).

In subgroup analyses based on comparison intensity and baseline BMI, there was evidence of an effect on restraint in favour of the intervention in trials comparing interventions to inactive control groups, which were the same trials in which the average baseline BMI of participants was between 25 and 35 (SMD 0.20 [95%CI 0.05, 0.34]; N = 3). Only one trial with an active comparison group and with participants baseline BMI above 35 reported outcomes of restraint at follow-up, finding no evidence of an effect on restraint (SMD 0.12 [95% CI -0.12, 0.36]).

6.7.2.1 Intervention type - Forest plots for impact on restraint by intervention type at follow-up

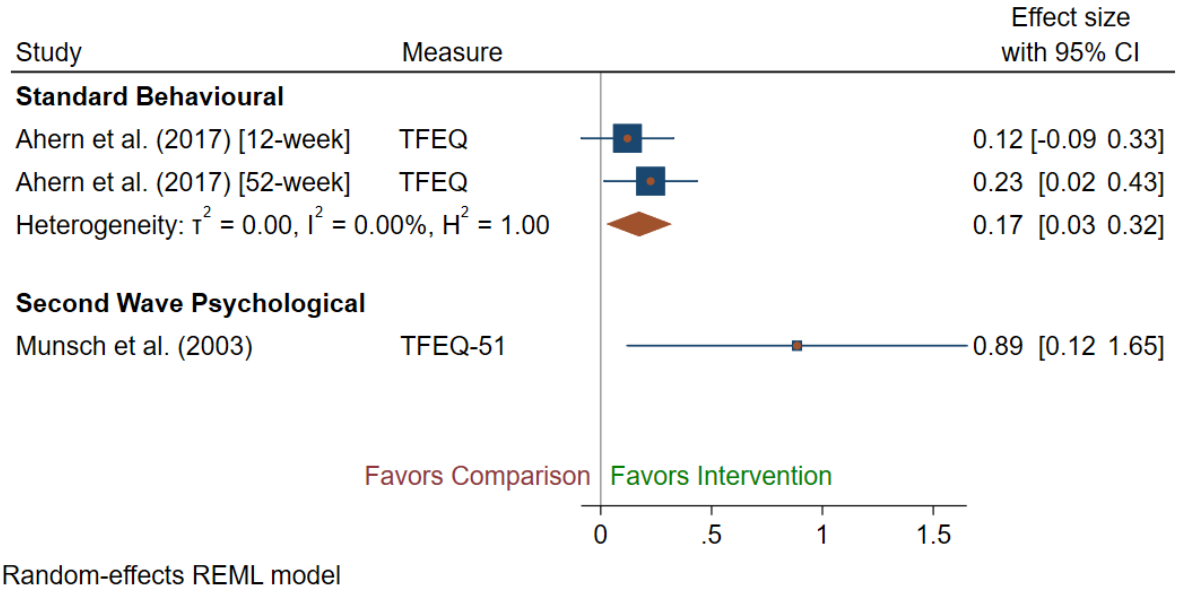

Figure S42 Post intervention restraint outcomes by intervention type at follow-up

6.7.2.2 Intervention duration - Forest plots for impact on restraint by intervention duration at follow-up

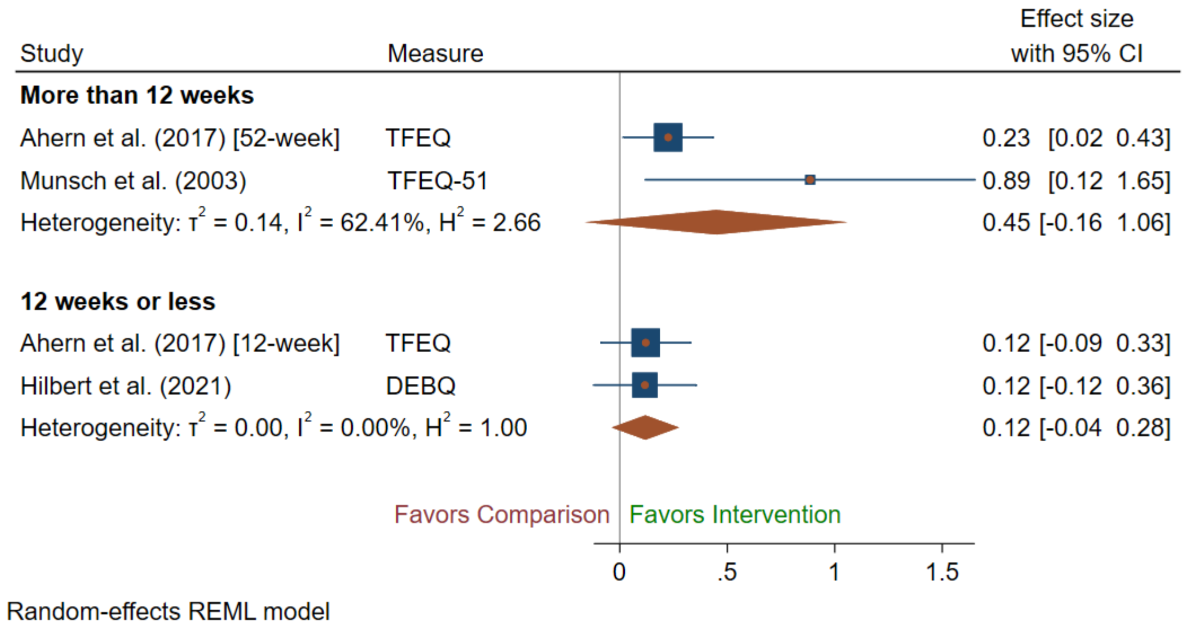

Figure S43 Post intervention restraint outcomes by intervention duration at follow-up

6.7.2.3 Intervention delivery format - Forest plots for impact on restraint by intervention delivery format at follow-up

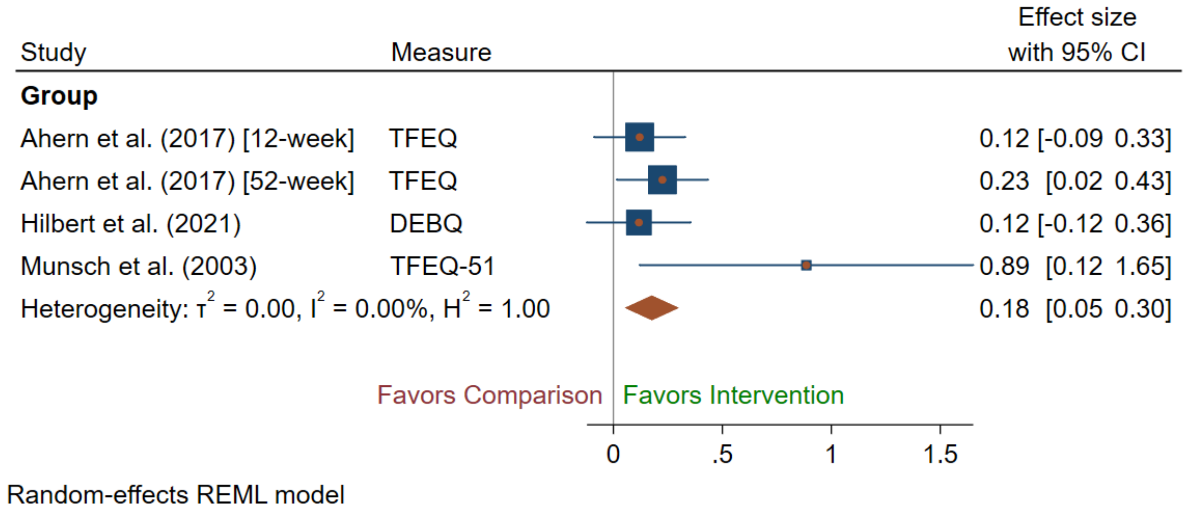

Figure S44 Post intervention restraint outcomes by intervention delivery format at follow-up

6.7.2.4 Intervention delivery mode - Forest plots for impact on restraint by intervention delivery mode at follow-up

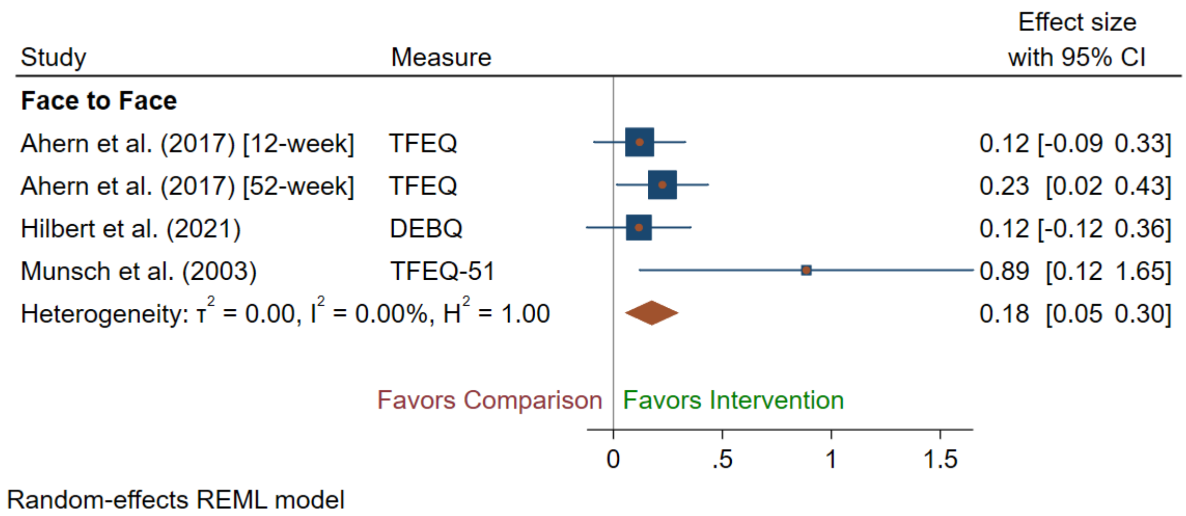

Figure S45 Post intervention restraint outcomes by intervention delivery mode at follow-up

6.7.2.5 Comparison intensity - Forest plots for impact on restraint by comparison intensity at follow-up

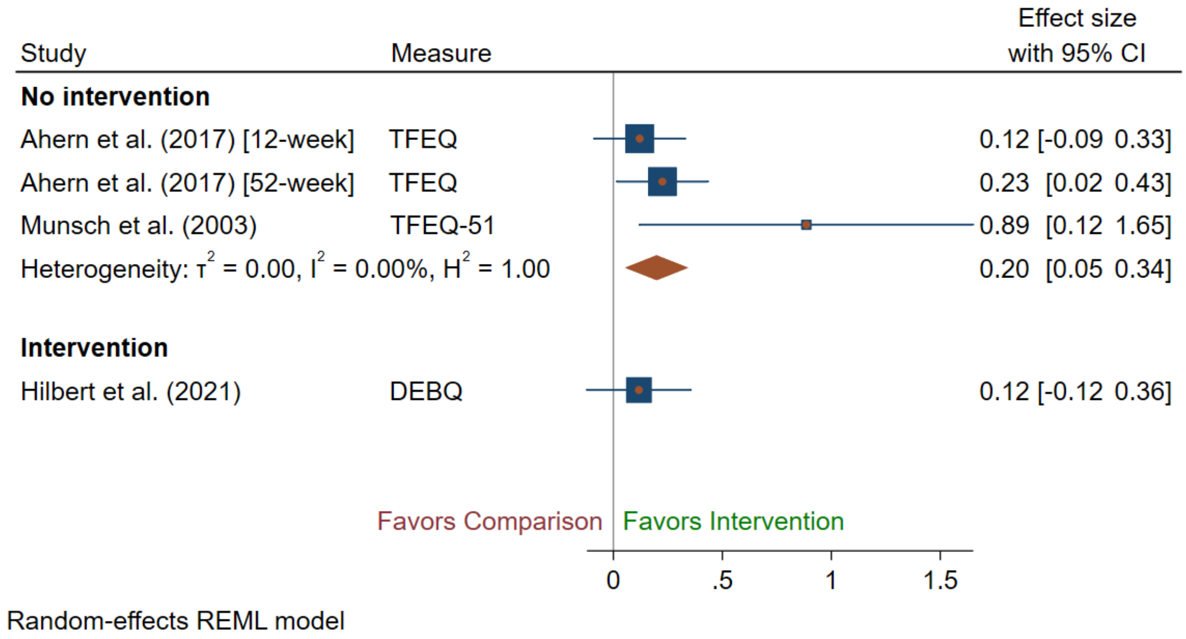

Figure S46 Post intervention restraint outcomes by comparison intensity at follow-up

6.7.2.6 Baseline BMI – Forest plots for impact on restraint by baseline BMI at follow-up

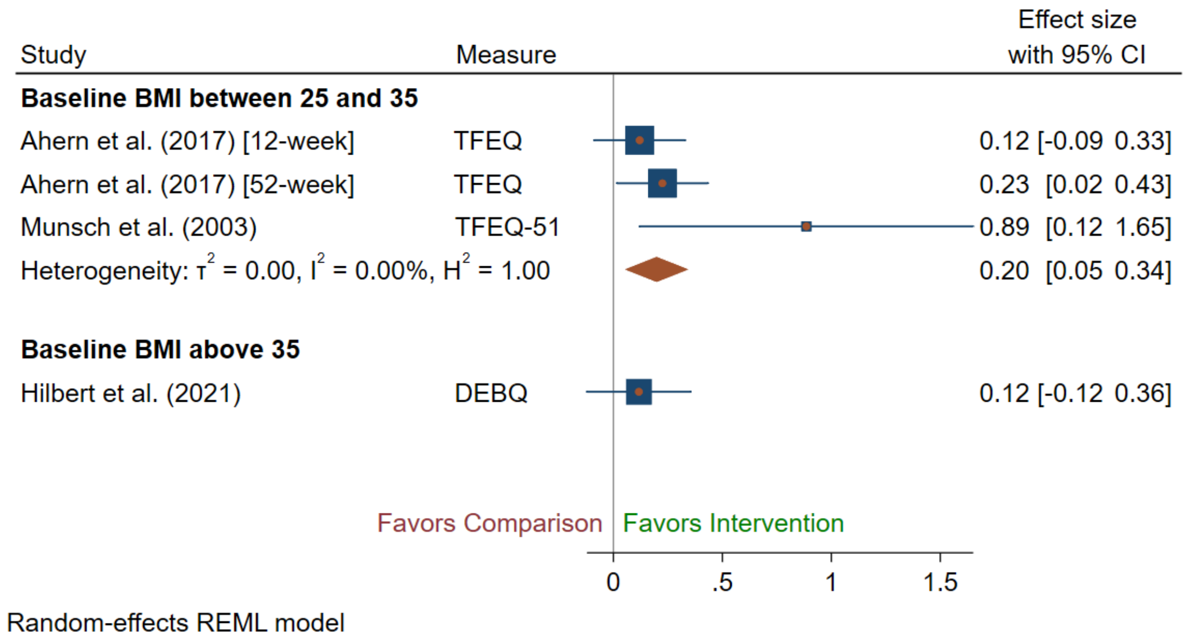

Figure S47 Post intervention restraint outcomes by baseline BMI at follow-up

6.8 Uncontrolled Eating

6.8.1 Impact on uncontrolled eating by subgroups at intervention end

Details on the impact of different subgroups on uncontrolled eating at intervention end are presented in Figures S48, S50, S52, S54, S56 and S58 for post intervention outcomes and in Figures S49, S51, S53, S55, S57, and S59 for change outcomes.

In subgroup analyses based on intervention type, there was evidence of an effect on uncontrolled eating in favour of the intervention in third wave cognitive behavioural therapy based interventions reporting post intervention outcomes (Post intervention: SMD -0.27 [95%-0.48, -0.07]; N = 4), but not in those reporting change outcomes (Change: SMD -0.29 [-0.68, 0.09], N = 4), and not in standard behavioural interventions (Post intervention: -0.17 [95%CI -0.39, 0.04], N =2; Change: -0.05 [95%CI -0.68, 0.09], N = 2).

Subgroup analyses by intervention duration found evidence of an effect on uncontrolled eating in favour of the intervention in interventions lasting 12 weeks or less and reporting post intervention outcomes (Post intervention: SMD -0.31 [95%CI -0.50, -0.12]; N = 5), but there was no evidence of an effect in intervention lasing 12 weeks or less that reported change outcomes (Change: SMD -0.13 [95%CI -0.54, 0.28], N = 4), nor in interventions of longer duration (Post intervention: SMD -0.17 [95%CI -0.39, 0.04], N = 2; Change: SMD -0.31 [95%CI -0.53, -0.10], N = 2).

In subgroup analyses based on intervention delivery format, there was evidence of an effect on uncontrolled eating in favour of the intervention in interventions delivered both on an individual (Post intervention: SMD -0.33 [95%CI -0.54, -0.11], N = 4) and group-based format for post intervention outcomes (Post intervention: SMD -0.18 [95%CI -0.34, -0.02], N = 3). For change outcomes, there was no evidence of an effect in both individual (Change: SMD -0.06 [95%CI -0.61, 0.49], N = 2) and group-based interventions (Change: SMD -0.30 [95%CI -0.87, 0.26], N = 3). Only one trial used a combination of both individual and mixed approaches and found evidence of an effect on uncontrolled eating in favour of the intervention for change outcomes (Change: SMD -0.28 [95%CI -0.52, -0.05]).

Similarly, in subgroup analyses based on intervention delivery mode, there was evidence of an effect on uncontrolled eating in favour of the intervention in interventions delivered both face-to-face (Post intervention: SMD -0.21 [95%CI -0.38, -0.04], N = 4) and remotely in trials reporting post intervention outcomes (Post intervention: SMD -0.31 [95%CI -0.54, -0.07], N = 3). For change outcomes, there was no evidence of an effect in both face-to-face (Change: SMD -0.28 [95%CI -0.67, 0.10], N = 4) and remote interventions (Change: SMD -0.06 [95%CI -0.61, 0.49], N = 2).

In subgroup analyses based on comparison intensity, there was evidence of an effect on uncontrolled eating in favour of the intervention in trials comparing interventions to inactive control groups (Post intervention: SMD -0.25 [95%CI -0.39, -0.10], N = 7; Change: SMD -0.30 [95%CI -0.43, -0.18], N = 2). There was no evidence of an effect in trial arms comparing interventions to active comparison groups (Change: SMD -0.16 [95%CI -0.63, 0.32], N = 4).

In subgroup analyses based on baseline BMI, there was evidence of an effect on uncontrolled eating in favour of the intervention in trials where participants had an average baseline BMI between 25 and 35 (Post intervention: SMD -0.25 [95%CI -0.39, -0.10], N = 7; Change: SMD -0.33 [95%CI -0.46, -0.21], N = 3). There was no evidence of an effect in trial arms where participants' baseline BMI was above 35 (Change: SMD 0.03 [95%CI -0.38, 0.45], N = 3).

6.8.1.1 Intervention type – Forest plots for impact on uncontrolled eating by intervention type at end of intervention

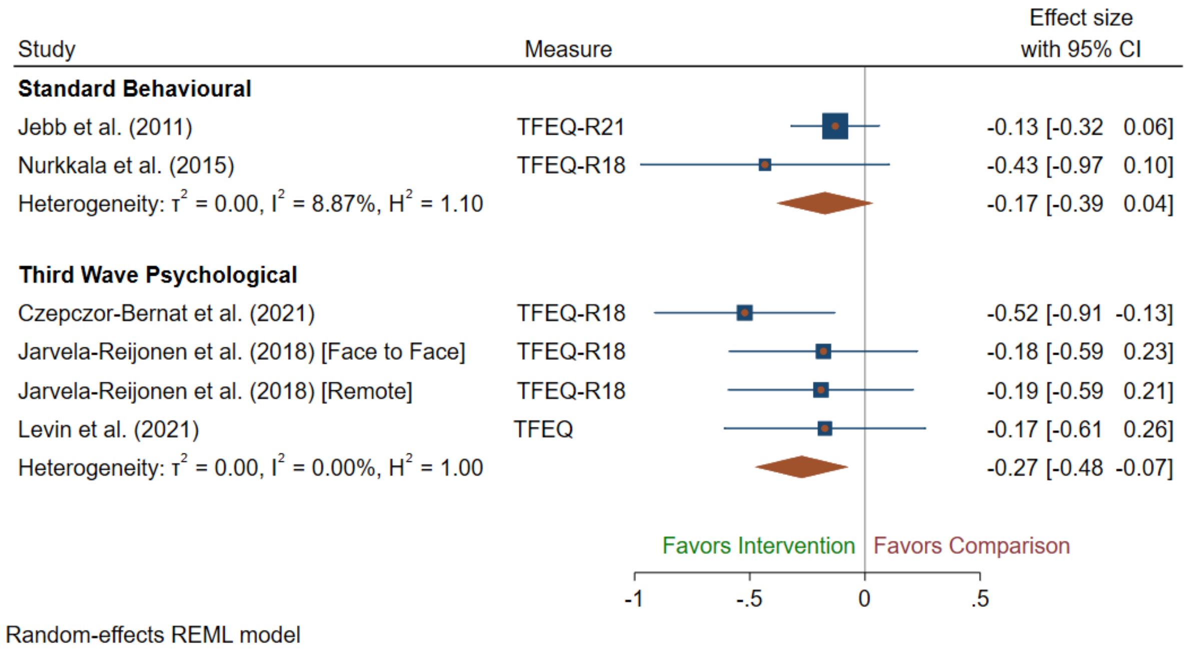

Figure S48 Post intervention uncontrolled eating outcomes by intervention type at intervention end

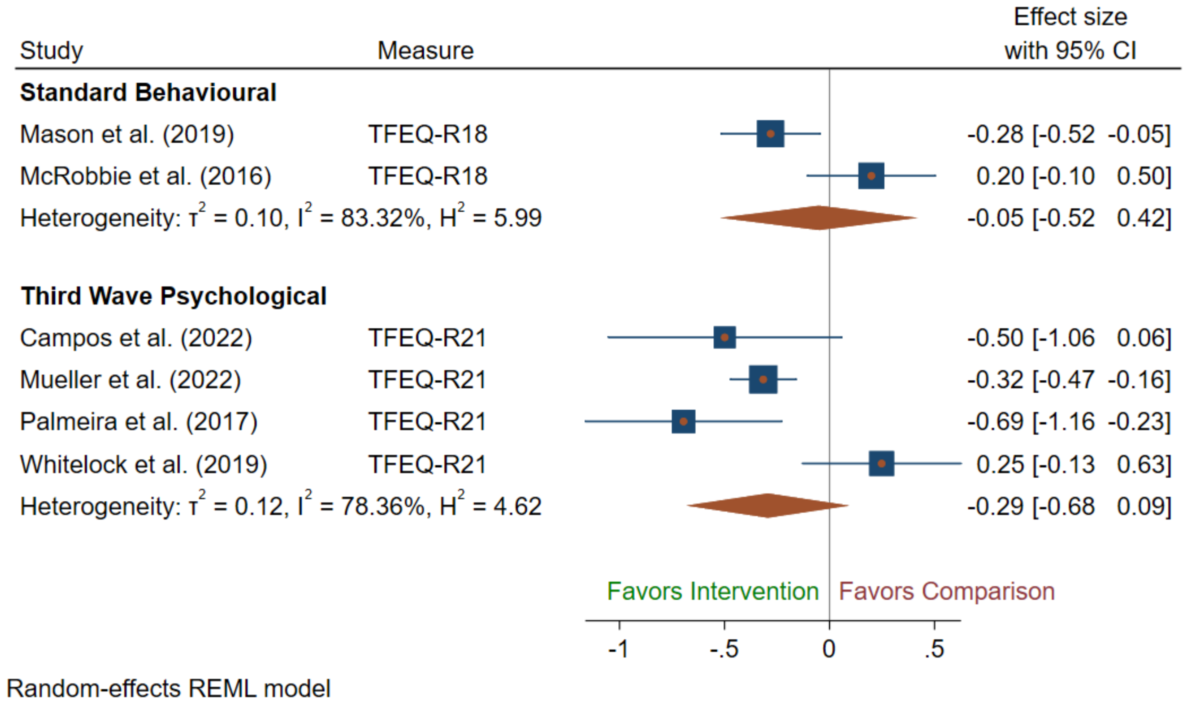

Figure S49 Change in uncontrolled eating outcomes by intervention type at intervention end

6.8.1.2 Intervention duration – Forest plots for impact on uncontrolled eating by intervention duration at end of intervention

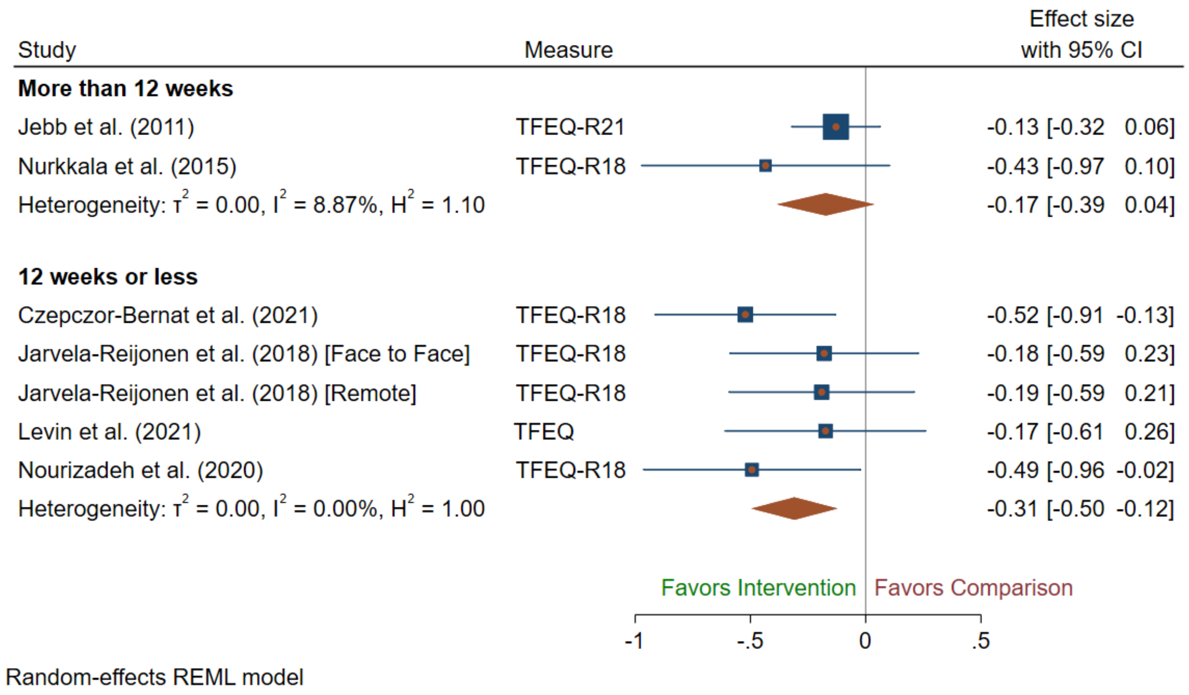

Figure S50 Post intervention uncontrolled eating outcomes by intervention duration at intervention end

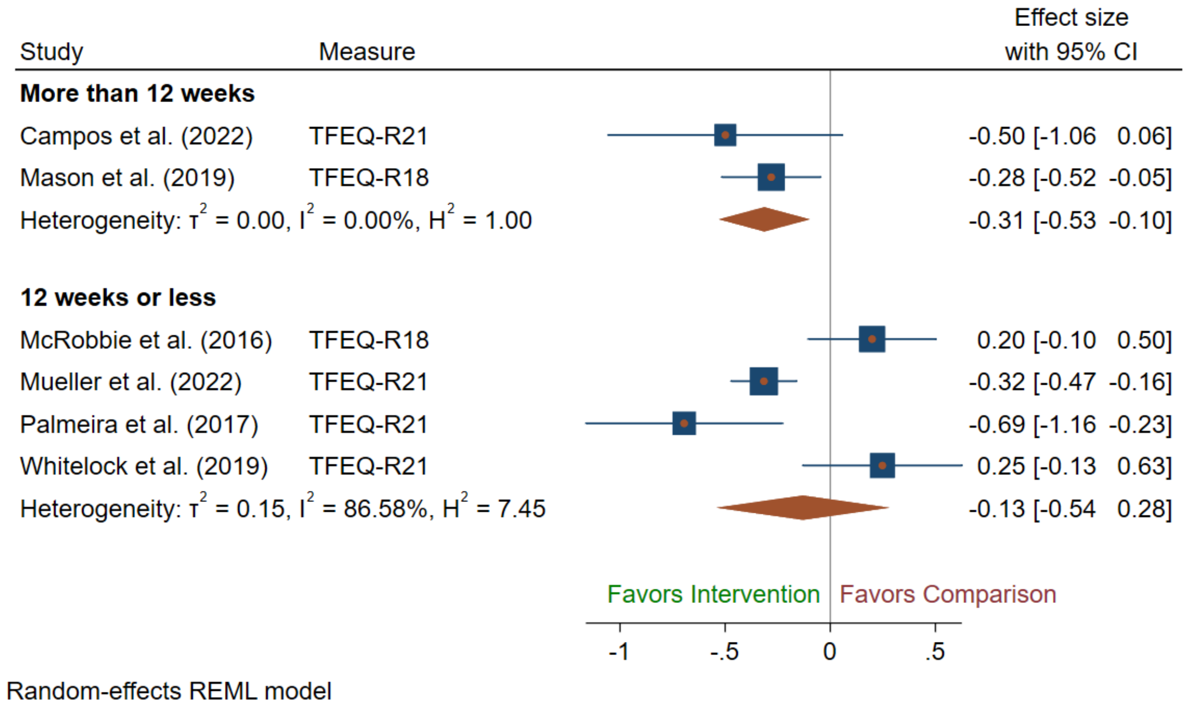

Figure S51 Change in uncontrolled eating outcomes by intervention duration at intervention end

6.8.1.3 Intervention delivery format – Forest plots for impact on uncontrolled eating by intervention delivery format at end of intervention

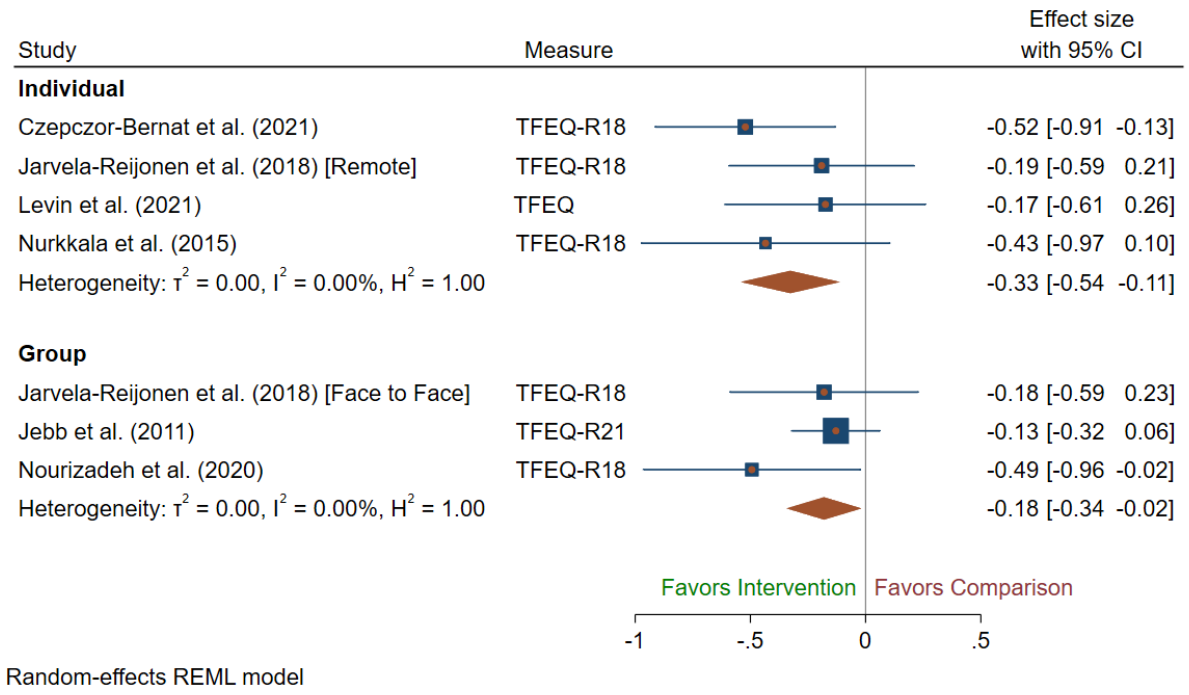

Figure S52 Post intervention uncontrolled eating outcomes by intervention delivery format at intervention end

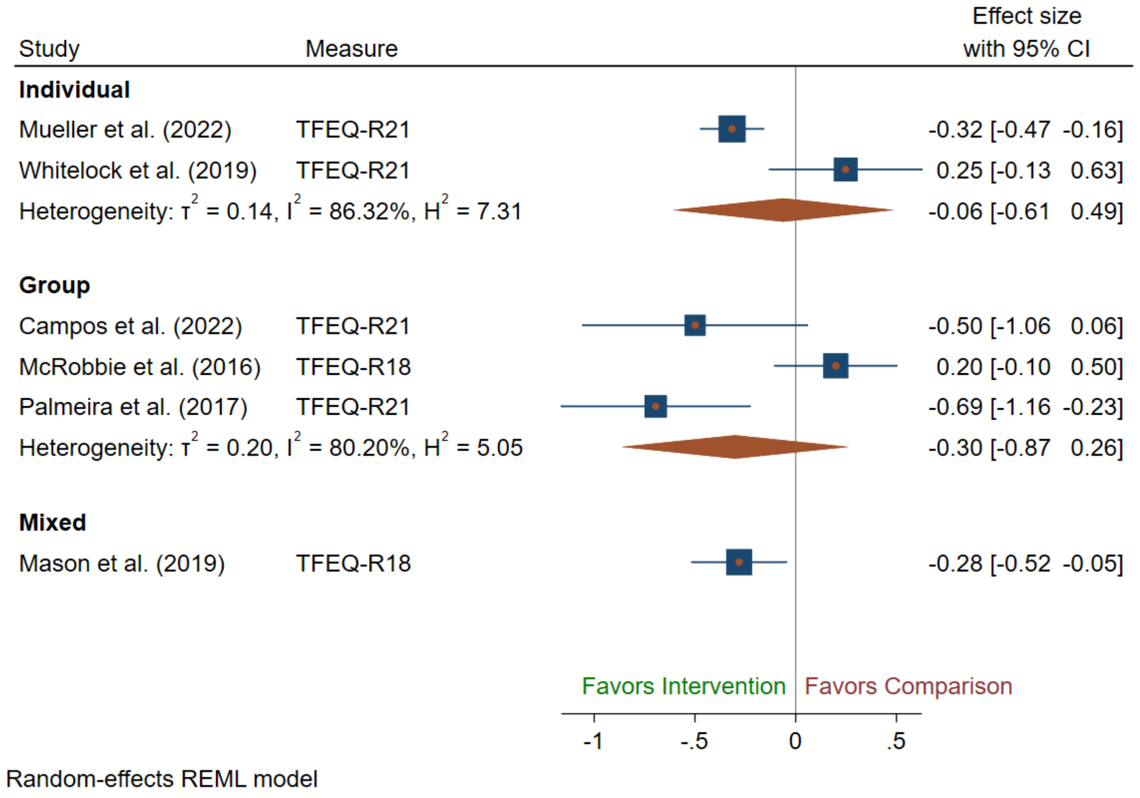

Figure S53 Change in uncontrolled eating outcomes by intervention delivery format at intervention end

6.7.1.4 Intervention delivery mode – Forest plots for impact on uncontrolled eating by intervention delivery mode at end of intervention

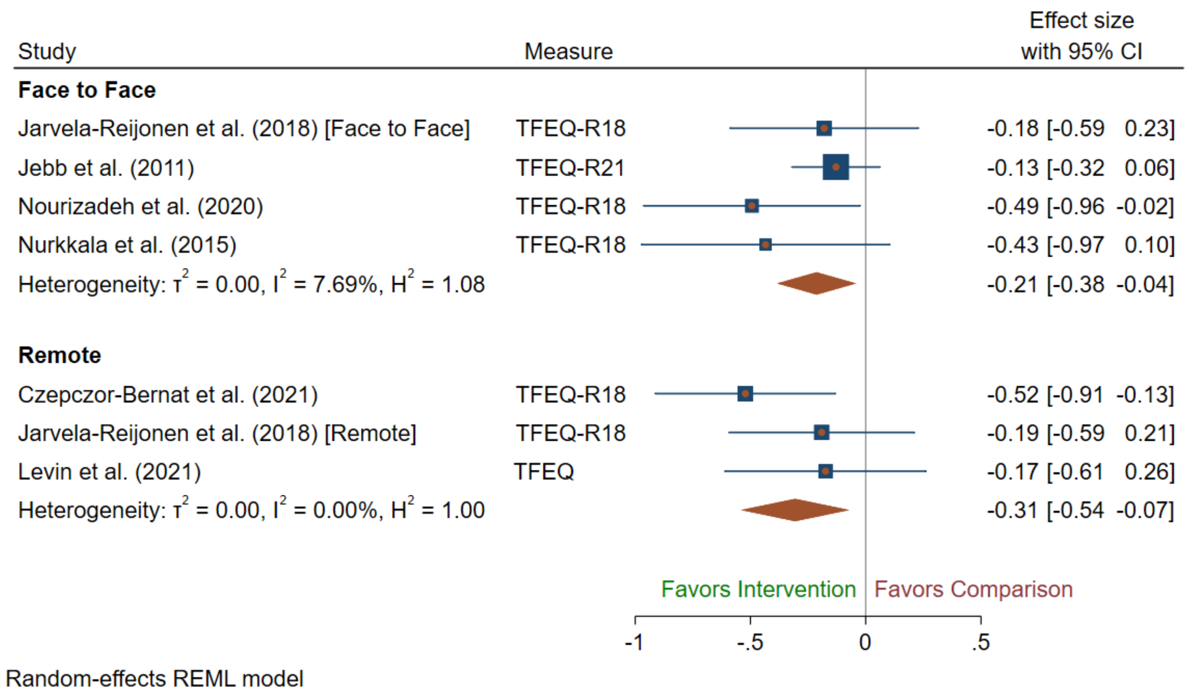

Figure S54 Post intervention uncontrolled eating outcomes by intervention delivery mode at intervention end

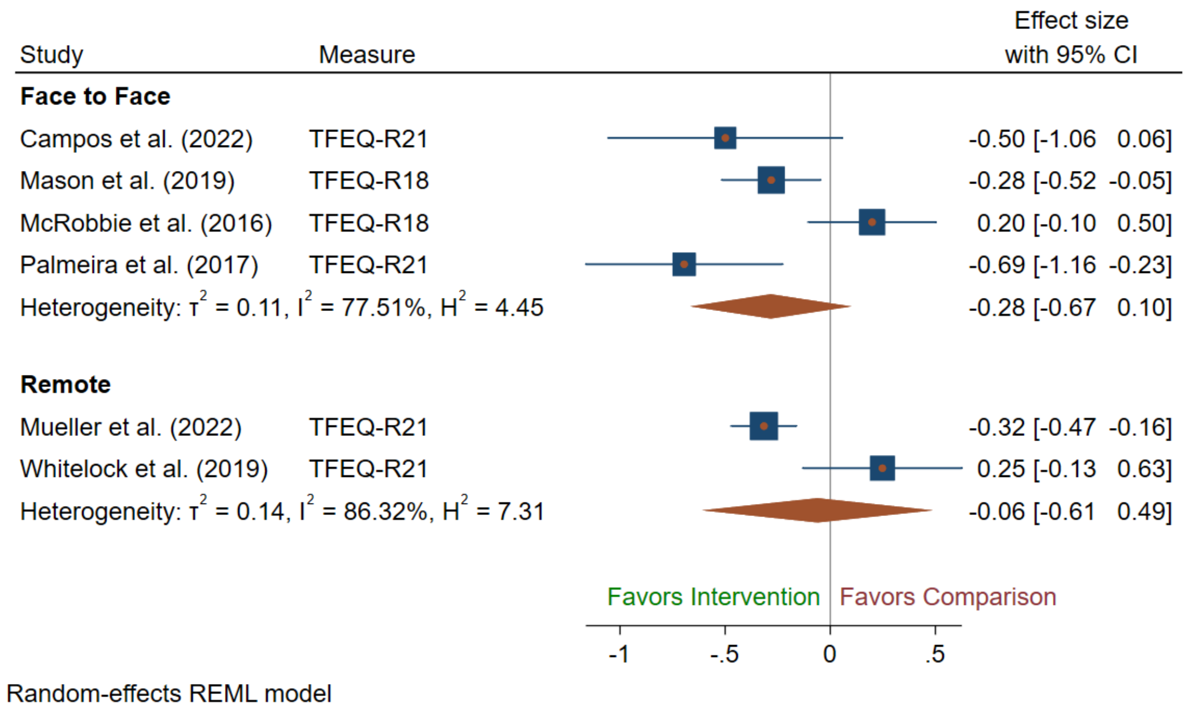

Figure S55 Change in uncontrolled eating outcomes by intervention delivery mode at intervention end

6.7.1.5 Comparison intensity – Forest plots for impact on uncontrolled eating by comparison intensity at intervention end

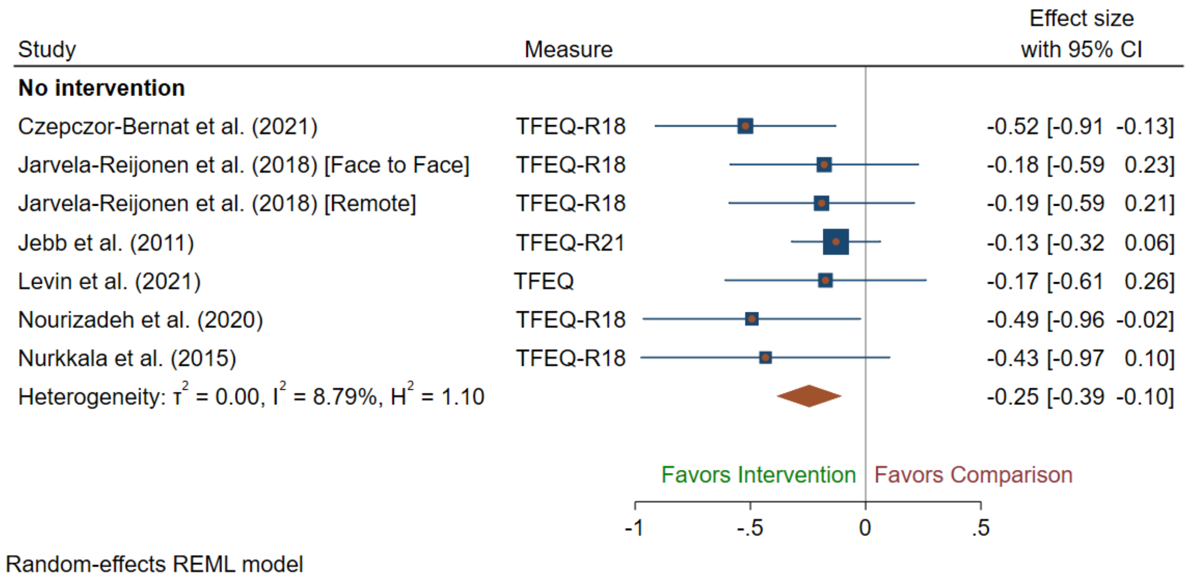

Figure S56 Post intervention uncontrolled eating outcomes by comparison intensity at intervention end

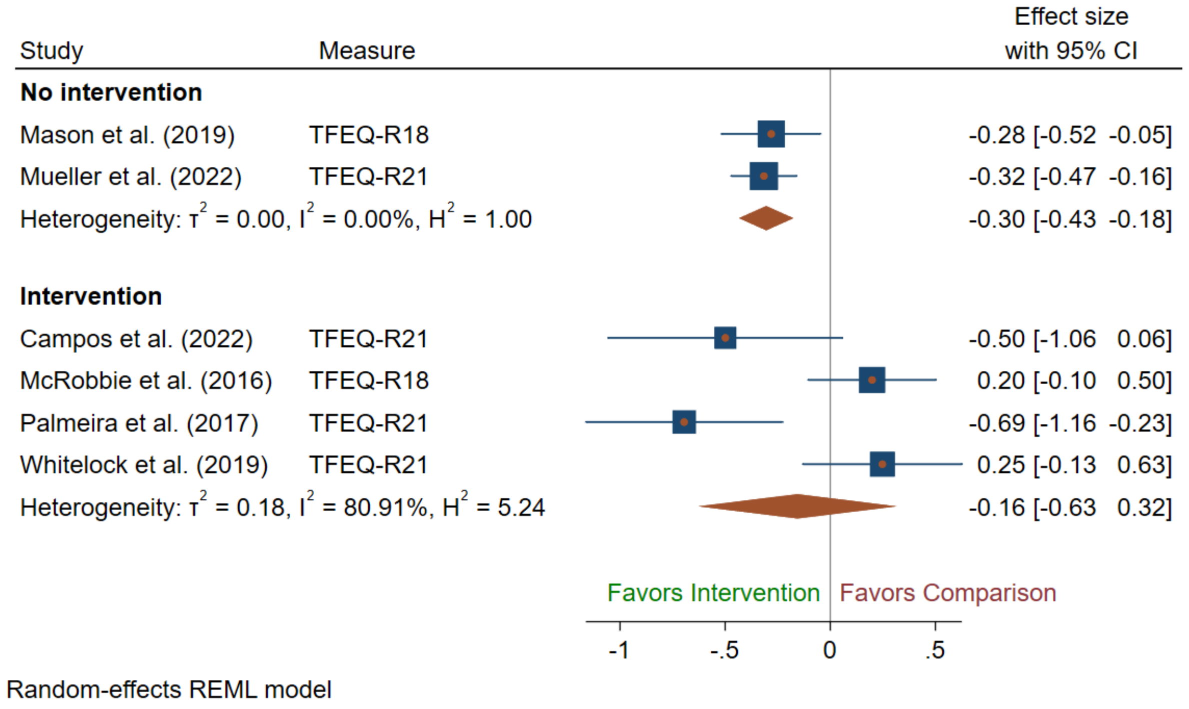

Figure S57 Change in uncontrolled eating outcomes by comparison intensity at intervention end

6.7.1.6 Baseline BMI – Forest plots for impact on uncontrolled eating by baseline BMI at end of intervention

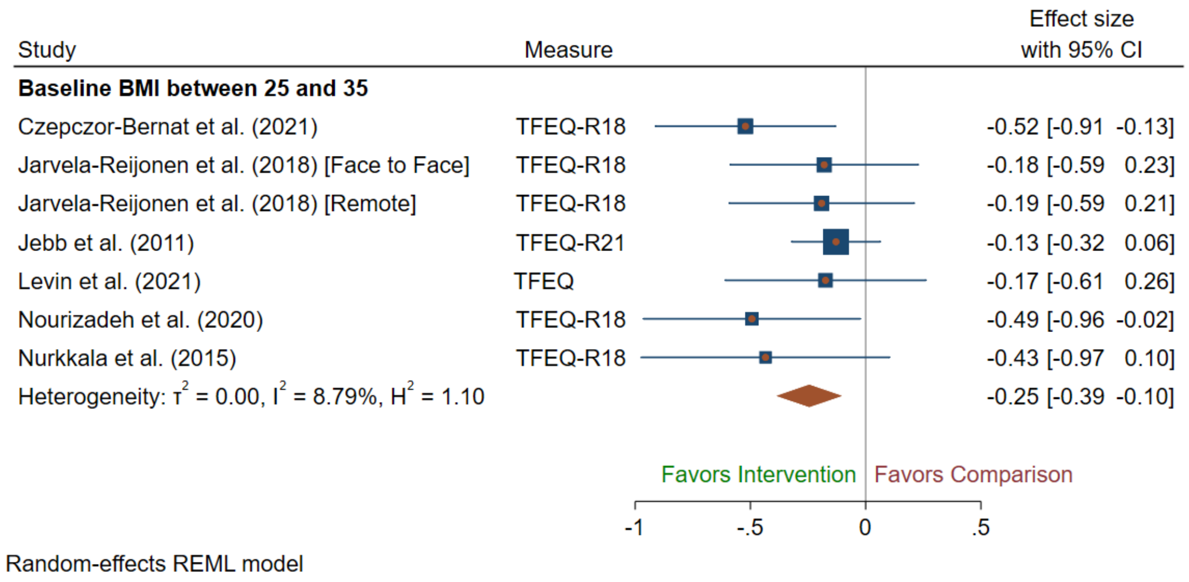

Figure S58 Post intervention uncontrolled eating outcomes by baseline BMI at intervention end

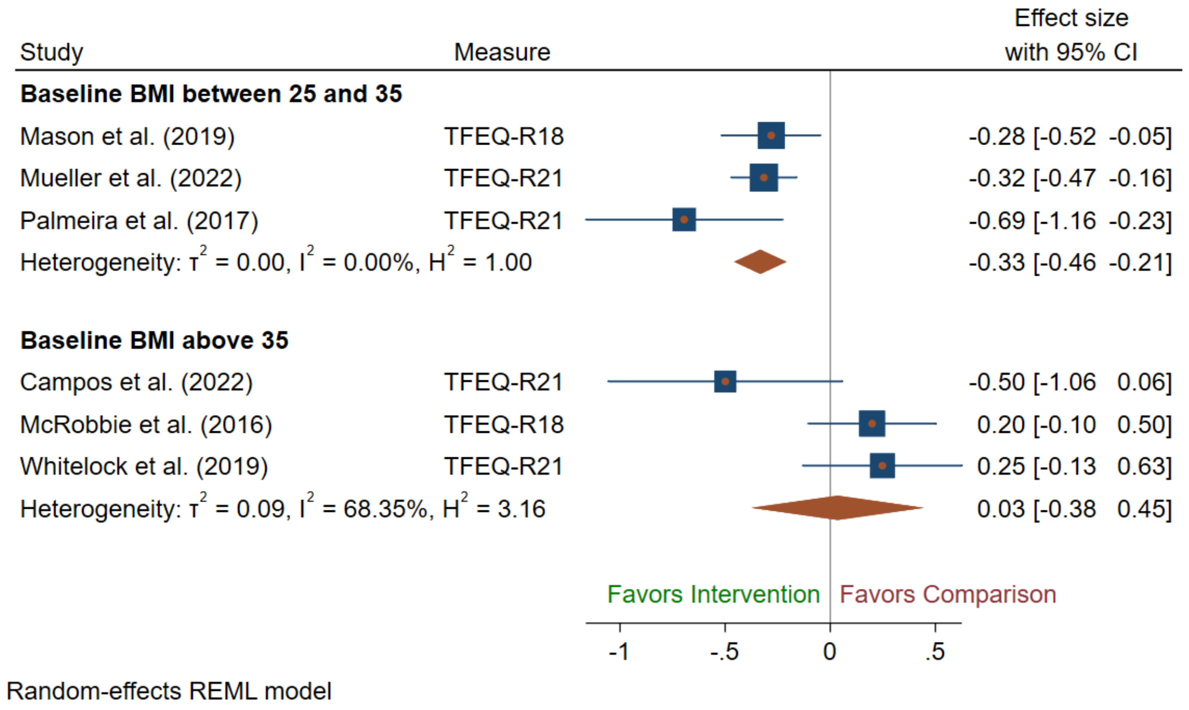

Figure S59 Change in uncontrolled eating outcomes by baseline BMI at intervention end

6.8.2 Impact on uncontrolled eating by subgroups at follow-up

No trials reported post intervention outcomes for uncontrolled eating at 12-months follow-up, and only two trials reported change outcomes. This was insufficient to conduct subgroup analyses.

6.9 Other EBTs

No subgroup analyses were performed for contextual skills, internal regulation, using food as a reward or pleasure of eating, since these outcomes were only reported in one trial.

7.0 Publication Bias

7.1 Disinhibition

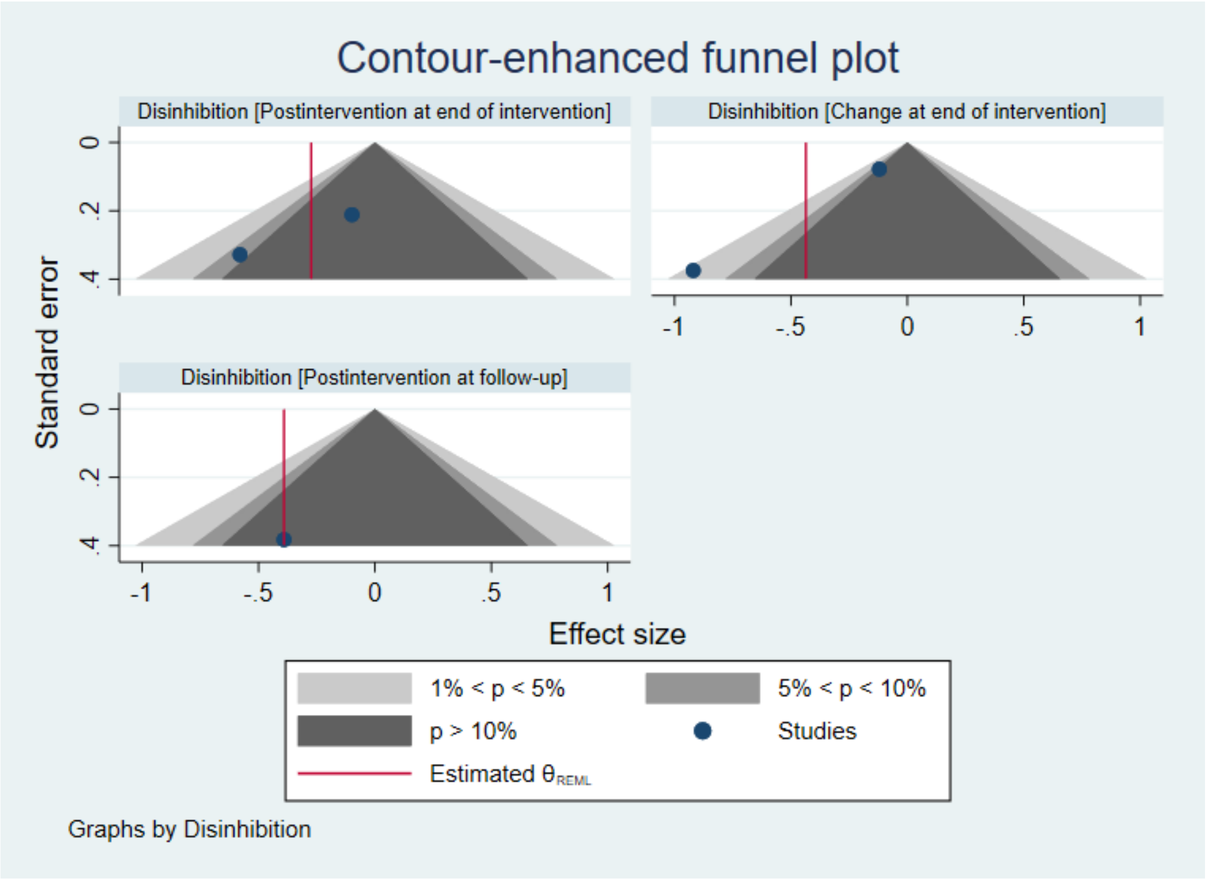

Figure S60 Contour enhanced funnel plots for disinhibition

7.2 Emotional Eating

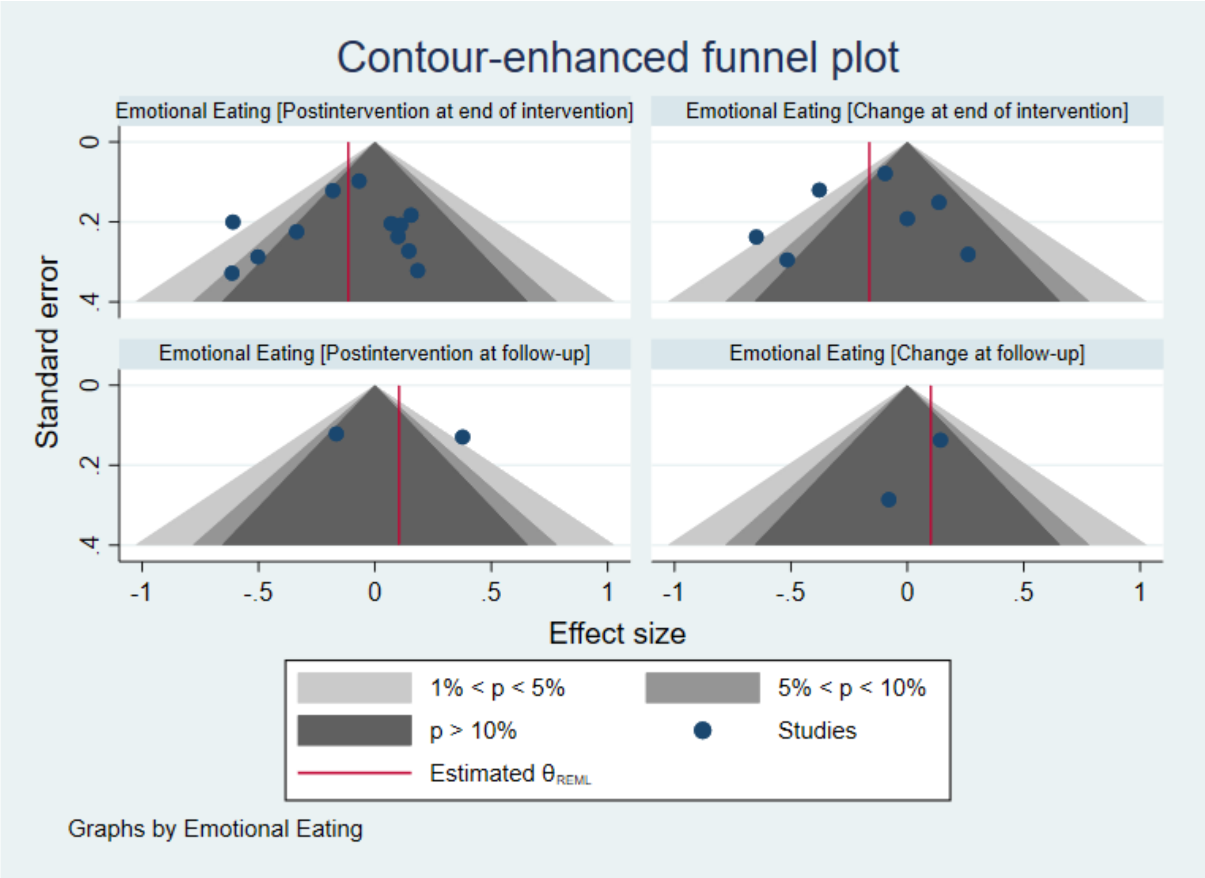

Figure S61 Contour enhanced funnel plots for emotional eating

7.3 External Eating

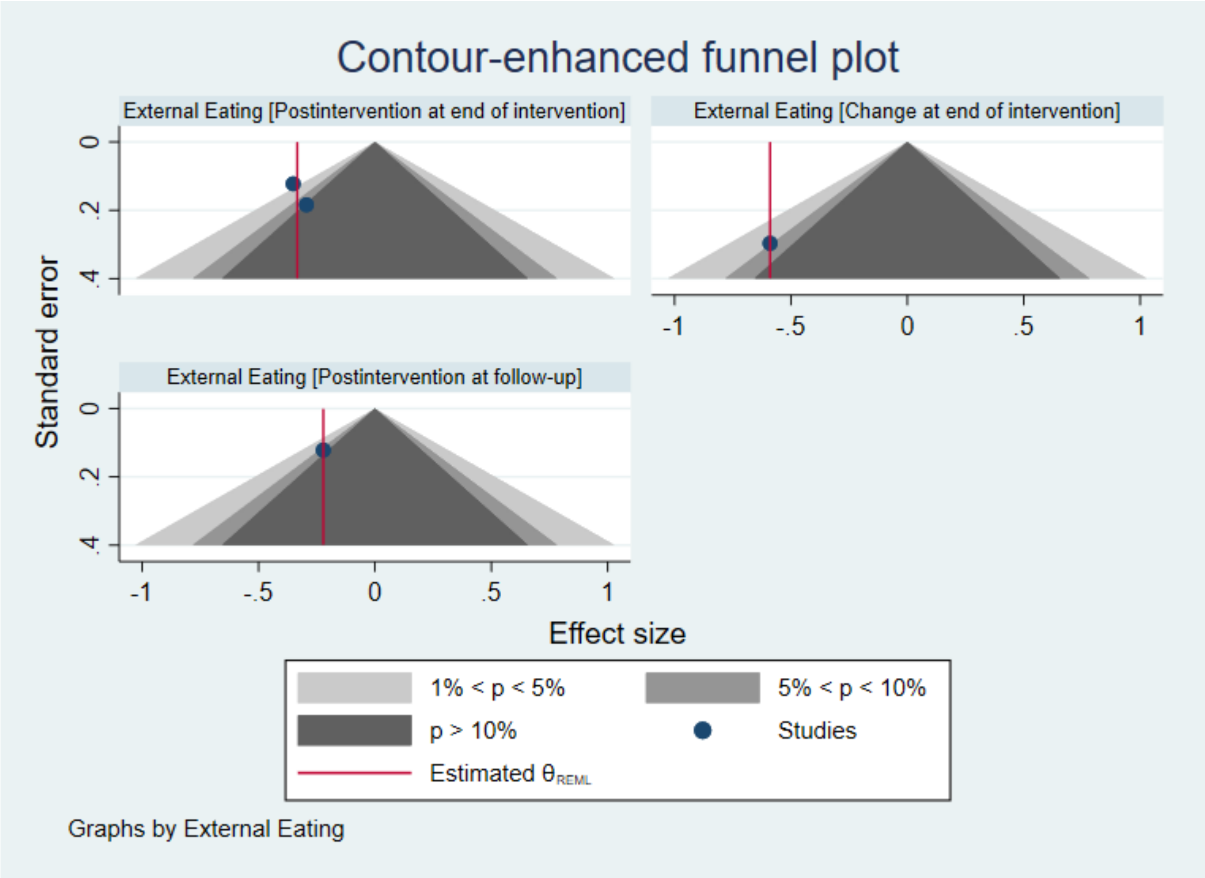

Figure S62 Contour enhanced funnel plots for external eating

7.4 Hunger, hedonic

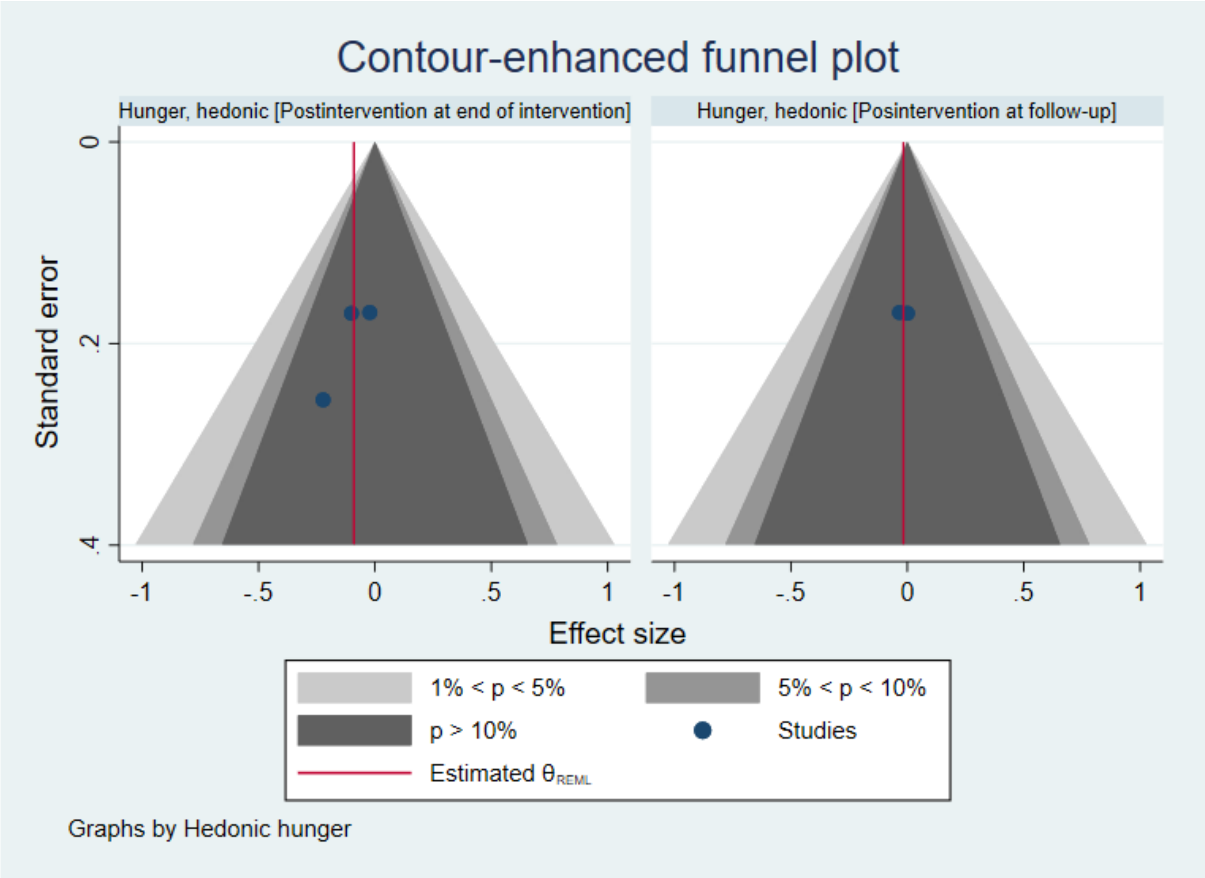

Figure S63 Contour enhanced funnel plots for hedonic hunger

7.5 Hunger, susceptibility

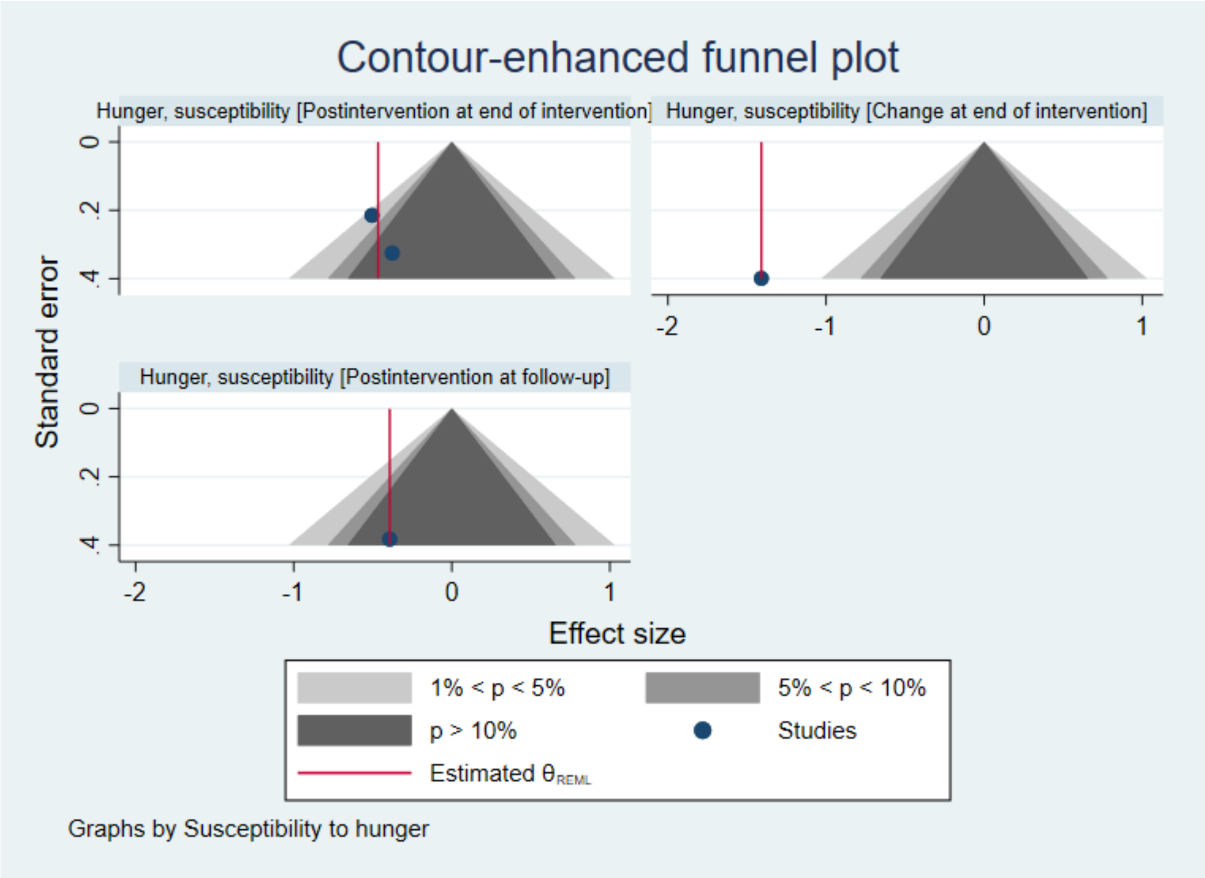

Figure S64 Contour enhanced funnel plots for susceptibility to hunger

7.6 Intuitive/ Mindful Eating

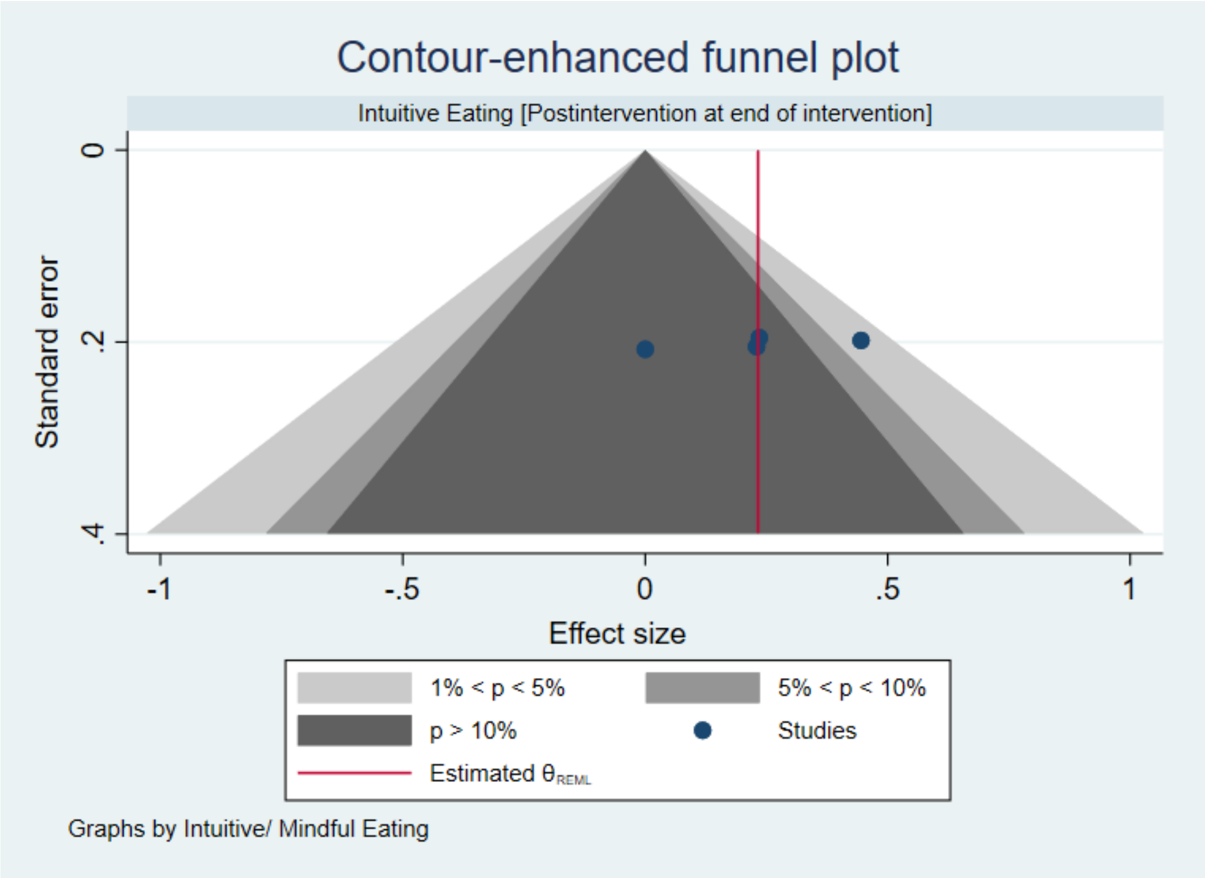

Figure S65 Contour enhanced funnel plots for intuitive/ mindful eating

7.7 Restraint

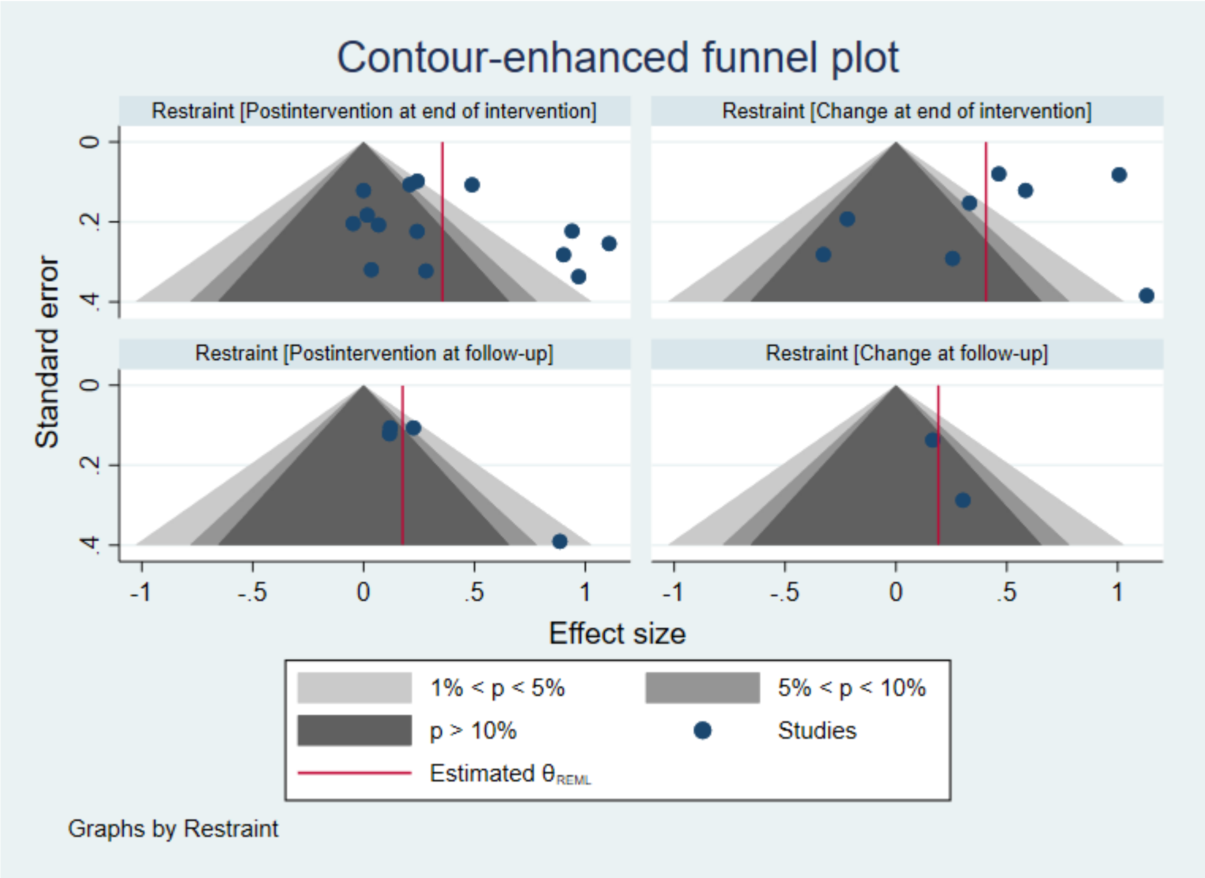

Figure S66 Contour enhanced funnel plots for restraint

7.8 Uncontrolled Eating

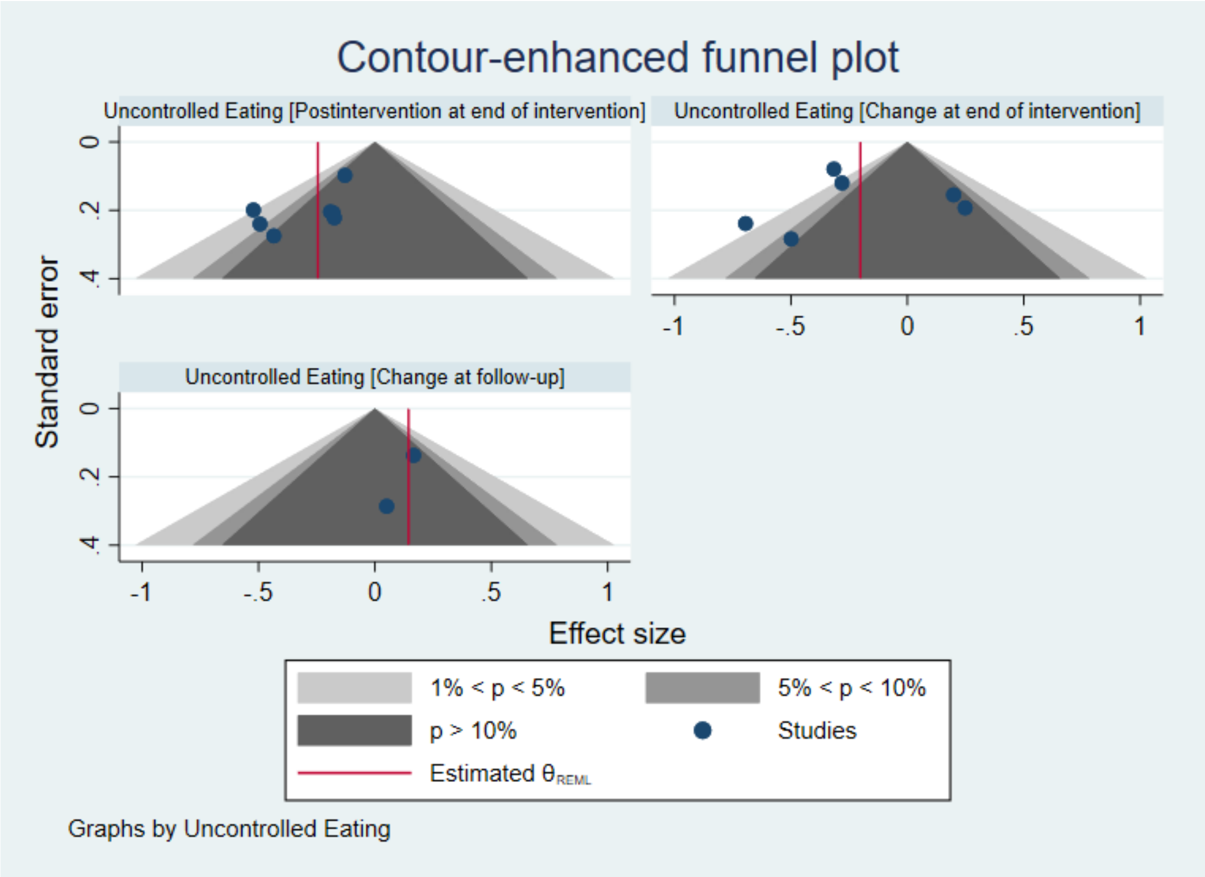

Figure S67 Contour enhanced funnel plots for uncontrolled eating

7.9 Other EBTs

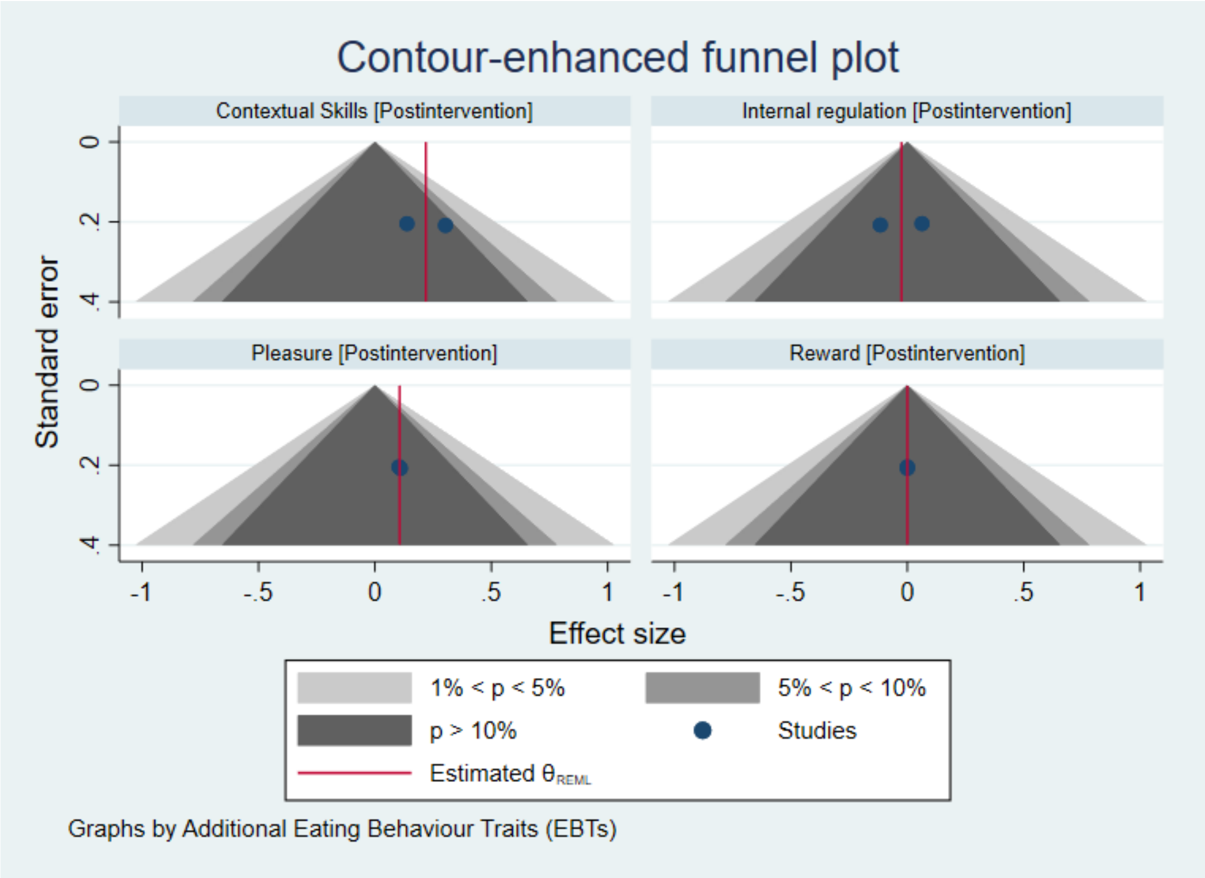

Figure S68 Contour enhanced funnel plots for contextual skills, internal regulation, pleasure of eating and eating as a reward at end of intervention

8.0 References

1. Bates S, Norman P, Breeze P, Brennan A, Ahern AL. Mechanisms of Action in a Behavioral Weight-Management Program: Latent Growth Curve Analysis. *Annals of Behavioral Medicine*. Published online 2021:1-14. doi:10.1093/abm/kaab019

2. Mead BR, Boyland EJ, Christiansen P, Halford JCG, Jebb SA, Ahern AL. Associations between hedonic hunger and bmi during a two-year behavioural weight loss trial. *PLoS One*. 2021;16(6 June). doi:10.1371/journal.pone.0252110

3. Ahern AL, Wheeler GM, Aveyard P, et al. Extended and standard duration weight-loss programme referrals for adults in primary care (WRAP): a randomised controlled trial. *The Lancet*. 2017;389(10085):2214-2225. doi:10.1016/S0140-6736(17)30647-5

4. Barnes RD, Ivezaj V, Martino S, Pittman BP, Grilo CM. Back to Basics? No Weight Loss from Motivational Interviewing Compared to Nutrition Psychoeducation at One-Year Follow-Up. *Obesity*. 2017;25(12):2074-2078. doi:10.1002/oby.21972

5. Camolas J, Santos O, Moreira P, do Carmo I. INDIVIDUO: Results from a patient-centered lifestyle intervention for obesity surgery candidates. *Obes Res Clin Pract*. 2017;11(4):475-488. doi:10.1016/j.orcp.2016.08.003

6. Campos MB, Menezes IHCF, Peixoto M do RG, Schincaglia RM. Intuitive eating in general aspects of eating behaviors in individuals with obesity: Randomized clinical trial. *Clin Nutr ESPEN*. 2022;50:24-32. doi:10.1016/j.clnesp.2022.06.002

7. Cassin SE, Sockalingam S, Du C, Wnuk S, Hawa R, Parikh S V. A pilot randomized controlled trial of telephone-based cognitive behavioural therapy for preoperative bariatric surgery patients. *Behaviour Research and Therapy*. 2016;80:17-22. doi:10.1016/j.brat.2016.03.001

8. Czepczor-Bernat K, Brytek-Matera A, Staniszewska A. The effect of a web-based psychoeducation on emotional functioning, eating behaviors, and body image among premenopausal women with excess body weight. *Arch Womens Ment Health*. Published online 2021. doi:10.1007/s00737-020-01077-1/Published

9. Daubenmier J, Kristeller J, Hecht FM, et al. Mindfulness intervention for stress eating to reduce cortisol and abdominal fat among overweight and obese women: An exploratory randomized controlled study. *J Obes*. 2011;2011. doi:10.1155/2011/651936

10. Hilbert A, Neuhaus P, Koehler N, Petroff D, Hay P, Huebner C. Group Cognitive Remediation Therapy Prior to Behavioral Weight Loss Treatment for Adults With Severe Obesity: A Randomized Clinical Trial (CRT Study). *J Consult Clin Psychol*. Published online 2021. doi:10.1037/ccp0000668.supp

11. Sairanen E, Tolvanen A, Karhunen L, et al. Psychological flexibility mediates change in intuitive eating regulation in acceptance and commitment therapy interventions. *Public Health Nutr*. 2017;20(9):1681-1691. doi:10.1017/S1368980017000441

12. Järvelä-Reijonen E, Karhunen L, Sairanen E, et al. The effects of acceptance and commitment therapy on eating behavior and diet delivered through face-to-face contact and a mobile app: A randomized controlled trial. *International Journal of Behavioral Nutrition and Physical Activity*. 2018;15(1). doi:10.1186/s12966-018-0654-8

13. Lappalainen R, Sairanen E, Järvelä E, et al. The effectiveness and applicability of different lifestyle interventions for enhancing wellbeing: The study design for a randomized controlled trial for persons with metabolic syndrome risk factors and psychological distress. *BMC Public Health*. 2014;14(1). doi:10.1186/1471-2458-14-310

14. Jebb SA, Ahern AL, Olson AD, et al. Primary care referral to a commercial provider for weight loss treatment versus standard care: A randomised controlled trial. *The Lancet*. 2011;378(9801):1485-1492. doi:10.1016/S0140-6736(11)61344-5

15. Katzmarzyk PT, Martin CK, Newton RL, et al. Weight Loss in Underserved Patients — A Cluster-Randomized Trial. *New England Journal of Medicine*. 2020;383(10):909-918. doi:10.1056/nejmoa2007448

16. Keränen AM, Savolainen MJ, Reponen AH, et al. The effect of eating behavior on weight loss and maintenance during a lifestyle intervention. *Prev Med (Baltim)*. 2009;49(1):32-38. doi:10.1016/j.ypmed.2009.04.011

17. Levin ME, Petersen JM, Durward C, et al. A randomized controlled trial of online acceptance and commitment therapy to improve diet and physical activity among adults who are overweight/obese. *Transl Behav Med*. 2021;11(6):1216-1225. doi:10.1093/tbm/ibaa123

18. Mason C, De Dieu Tapsoba J, Duggan C, Wang CY, Alfano CM, McTiernan A. Eating behaviors and weight loss outcomes in a 12-month randomized trial of diet and/or exercise intervention in postmenopausal women. *International Journal of Behavioral Nutrition and Physical Activity*. 2019;16(1). doi:10.1186/s12966-019-0887-1

19. McRobbie H, Hajek P, Peerbux S, et al. Tackling obesity in areas of high social deprivation: Clinical effectiveness and cost-effectiveness of a task-based weight management group programme – A randomised controlled trial and economic evaluation. *Health Technol Assess (Rockv)*. 2016;20(79):1-150. doi:10.3310/hta20790

20. Mueller J, Richards R, Jones RA, et al. Supporting Weight Management during COVID-19: A Randomized Controlled Trial of a Web-Based, ACT-Based, Guided Self-Help Intervention. *Obes Facts*. 2022;15(4):550-559. doi:10.1159/000524031

21. Munsch S, Biedert E, Keller U. *Evaluation of a Lifestyle Change Programme for the Treatment of Obesity in General Practice*.; 2003.

22. Nourizadeh R, Azami S, Farshbaf-Khalili A, Mehrabi E. The Effect of Motivational Interviewing on Women with Overweight and Obesity Before Conception. *J Nutr Educ Behav*. 2020;52(9):859-866. doi:10.1016/j.jneb.2020.04.219

23. Nurkkala M, Kaikkonen K, Vanhala ML, Karhunen L, Keränen AM, Korpelainen R. Lifestyle intervention has a beneficial effect on eating behavior and long-term weight loss in obese adults. *Eat Behav*. 2015;18:179-185. doi:10.1016/j.eatbeh.2015.05.009

24. Palmeira L, Pinto-Gouveia J, Cunha M. Exploring the efficacy of an acceptance, mindfulness & compassionate-based group intervention for women struggling with their weight (Kg-Free): A randomized controlled trial. *Appetite*. 2017;112:107-116. doi:10.1016/j.appet.2017.01.027

25. Paul L, Van Der Heiden C, Van Hoeken D, et al. Cognitive Behavioral Therapy Versus Usual Care Before Bariatric Surgery: One-Year Follow-Up Results of a Randomized Controlled Trial. *Obes Surg*. Published online 2021. doi:10.1007/s11695-020-05081-3/Published

26. Porca C, Rodriguez-Carnero G, Tejera C, et al. Effectiveness to promote weight loss maintenance and healthy lifestyle habits of a group educational intervention program in adults with obesity: IGOBE program. *Obes Res Clin Pract*. 2021;15(6):570-578. doi:10.1016/j.orcp.2021.10.003

27. Potts S, Krafft J, Levin ME. A Pilot Randomized Controlled Trial of Acceptance and Commitment Therapy Guided Self-Help for Overweight and Obese Adults High in Weight Self-Stigma. *Behav Modif*. 2022;46(1):178-201. doi:10.1177/0145445520975112

28. Roehling M, Martin K, Ellinger S, Schreiber M, Martin S, Kempf K. Weight Reduction by the Low-Insulin-Method- A Randomized Controlled Trial. *Nutrients*. 2019;68(Supplement_1). doi:10.2337/db19-2050-p

29. Salvo V, Curado DF, Sanudo A, et al. Comparative effectiveness of mindfulness and mindful eating programmes among low-income overweight women in primary health care: A randomised controlled pragmatic study with psychological, biochemical, and anthropometric outcomes. *Appetite*. 2022;177. doi:10.1016/j.appet.2022.106131

30. Steinberg DM, Tate DF, Bennett GG, Ennett S, Samuel-Hodge C, Ward DS. Daily self-weighing and adverse psychological outcomes: A randomized controlled trial. *Am J Prev Med*. 2014;46(1):24-29. doi:10.1016/j.amepre.2013.08.006

31. Whitelock V, Kersbergen I, Higgs S, Aveyard P, Halford JCG, Robinson E. A smartphone based attentive eating intervention for energy intake and weight loss: results from a randomised controlled trial. *BMC Public Health*. Published online 2019. doi:10.17605/osf.io/btzhw
